# Genetic variants influencing liver fat in normal-weight individuals of European ancestry

**DOI:** 10.1016/j.jhepr.2025.101453

**Published:** 2025-05-14

**Authors:** Ignazio S. Piras, Janith Don, Nicholas J. Schork, Johanna K. DiStefano

**Affiliations:** Translational Genomics Research Institute, Phoenix, AZ, USA

**Keywords:** GWAS, Hepatic steatosis, TWAS, Fine mapping, MASLD

## Abstract

**Background & Aims:**

Metabolic dysfunction-associated steatotic liver disease (MASLD) occurs across a wide spectrum of body weights, yet the genetic determinants underlying hepatic steatosis in individuals with normal BMI remain underexplored. This study aimed to identify genetic variants associated with liver fat fraction in normal-weight individuals.

**Methods:**

We performed a genome-wide association study (GWAS) using magnetic resonance imaging-proton density fat fraction (MRI-PDFF) data from 10,918 normal-weight participants (BMI <25 kg/m^2^) of European ancestry in the UK Biobank. Hepatic steatosis and liver fat content were assessed using both case–control (CC; 815 cases with MRI-PDFF ≥5% *vs*. 10,103 controls with MRI-PDFF <5%) and quantitative trait (QT; N = 10,918, with MRI-PDFF as a continuous outcome) designs. Fine mapping prioritized potential causal variants. Gene-level associations were evaluated using multi-marker analysis of genomic annotation (MAGMA), and liver-specific gene expression was imputed for transcriptome-wide association studies (TWAS).

**Results:**

We identified 241 genome-wide significant variants in the CC-GWAS and 418 in the QT-GWAS, with most located on chromosomes 19 and 22, including known loci such as *PNPLA3*, *TM6SF2*, and *SAMM50*. Fine-mapping analyses prioritized three candidate causal variants in *SUGP1*, *GATAD2A*, and *MAU2*. MAGMA identified eight genes in CC-GWAS and 19 in QT-GWAS, including a novel association with *RFXANK*. TWAS supported the involvement of *MBOAT7* and *SAMM50*, with fine mapping further implicating *SAMM50* as a likely causal gene.

**Conclusions:**

This study, one of the first to detect genome-wide associations for hepatic steatosis in normal-weight individuals, identified both novel and established genetic loci. These findings highlight the role of genetic susceptibility independent of obesity-related pathways and may inform targeted strategies for MASLD prevention and treatment in this understudied population.

**Impact and implications:**

This study provides new insights into the genetic risk factors underlying metabolic dysfunction-associated steatotic liver disease in individuals with a normal BMI, a group often under-represented in steatotic liver disease research. Leveraging large-scale genomic and imaging data from the UK Biobank, we identified both known and novel variants associated with liver fat accumulation, emphasizing that genetic predisposition can drive hepatic steatosis independently of excess adiposity. While the study is based on individuals of European ancestry, future research should assess the relevance of these findings in more diverse populations to ensure broader clinical applicability. These results may help inform future strategies for early risk stratification and targeted prevention in metabolically vulnerable, normal-weight individuals.

## Highlights

• GWAS identifies MASLD risk variants on chromosomes 19 and 22 in normal-weight individuals.
• Fine mapping nominates *SUGP1*, *GATAD2A*, and *MAU2* as novel loci for hepatic fat accumulation.
• TWAS highlights *SAMM50* as a key driver of hepatic fat accumulation independent of adiposity.
• MAGMA and TWAS reveal new gene associations, including *RFXANK* and *MBOAT7*.
• Findings underscore genetic factors influencing MASLD risk beyond excess adiposity.

## Graphical abstract

**Figure:**
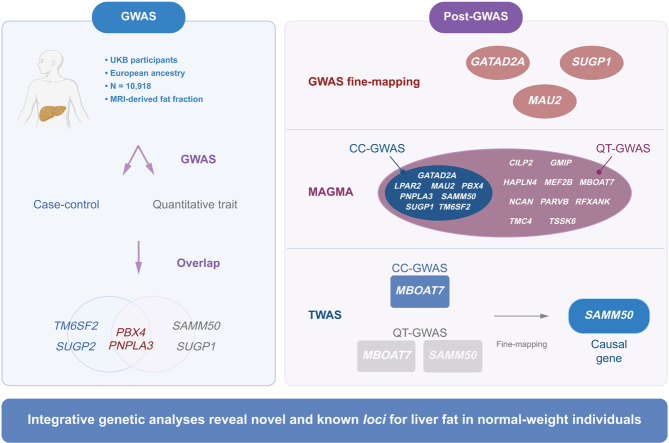

## Introduction

Obesity is an important risk factor for the development of metabolic dysfunction-associated steatotic liver disease (MASLD).^1^ Patients with MASLD with obesity are more likely to develop metabolic dysfunction-associated steatohepatitis (MASH), often accompanied by hepatic fibrosis, and experience greater liver-related mortality compared with patients with MASLD with overweight.^2^^,^^3^ While MASLD prevalence increases in parallel with BMI,^3^ the relationship between obesity and MASLD is complex, because many individuals with obesity maintain normal intrahepatic triglyceride (IHTG) content and metabolic function,^2^ even following moderate weight gain.^4^ Excessive IHTG content in individuals with obesity serves as a strong indicator of metabolic abnormalities, including hepatic, skeletal muscle, and adipose tissue insulin resistance, and dysfunctional free fatty acid metabolism, independent of BMI, percent body fat, and visceral fat mass.^2^ By contrast, normal IHTG content may be protective against the development of obesity-related metabolic complications.^2^ In individuals with normal weight, IHTG content is associated with metabolic dysfunction.^5^ However, in these individuals, hepatic steatosis can be present without insulin resistance, type 2 diabetes mellitus, or related metabolic comorbidities,^6^ indicating that excessive liver fat accumulation is not solely dependent on adiposity and can occur in individuals without accompanying metabolic abnormalities.

Numerous studies have compared the characteristics of MASLD among individuals belonging to obese (BMI ≥30 kg/m^2^/BMI ≥25 kg/m^2^ for Asians), non-obese (BMI <30 kg/m^2^/BMI <25 kg/m^2^ for Asians), and normal (BMI <25 kg/m^2^/BMI <23 kg/m^2^ for Asians) BMI categories.[7], [8], [9] Despite some overlap in clinical presentation among BMI groups, individuals with normal BMI generally exhibit a more favorable metabolic profile, less severe liver disease, and slower disease progression. The characteristics associated with patients with MASLD with a normal BMI differ from those observed in individuals with a BMI ≥25 kg/m^2^,^10^ suggesting a complex relationship between adiposity and disease presentation.

Genetic factors might mediate the development of hepatic steatosis in individuals with normal weight. The *PNPLA3* rs738409 variant has been extensively studied in normal-weight patients with MASLD.[11], [12], [13] Normal-weight carriers of the *PNPLA3* rs738409 GG genotype exhibited the highest MASLD risk when compared with individuals with overweight or obesity,^11^ and the impact of rs738409 on MASLD prevalence was more pronounced in individuals with normal weight compared with those with overweight.^14^ Some studies have reported no significant differences in *PNPLA3* genotypes among patients with MASLD with normal BMI and those in other BMI classes.[15], [16], [17] Variants in other genes have also been associated with MASLD in individuals within normal BMI.[18], [19], [20], [21], [22]

Despite the evidence linking specific variants to MASLD in individuals with normal weight, the investigation of genetic factors in this population remains sparse. Most genetic studies investigating this condition have focused on candidate genes, which are limited by small effect sizes when considered in a genome-wide context and often lack proper adjustment for population structure. To date, only two genome-wide association studies (GWAS) in normal-weight individuals have been reported, with only one demonstrating statistically significant results at the genome-wide level.^23^^,^^24^ Considering the disparities in MASLD prevalence and risk factors between individuals with normal weight and those with overweight or obesity, we hypothesized that distinct genetic variants might contribute to the susceptibility of MASLD in individuals with a normal BMI. Thus, we conducted a GWAS to identify genetic variants associated with liver fat specifically in individuals belonging to the normal BMI category. To achieve this, we analyzed magnetic resonance imaging-proton density hepatic fat fraction (MRI-PDFF) as both a discrete trait and quantitative trait (QT), in a cohort of 10,918 individuals with European ancestry in the normal BMI category from the UK Biobank (UKB). We also performed post-GWAS analyses, including fine mapping to identify potential causal variants, derivation of gene-level statistics (MAGMA), and a transcriptome-wide association study (TWAS) to identify associations between gene expression and hepatic fat levels. Through this multi-analysis approach, we sought to identify and characterize specific genetic variants that contribute to hepatic fat accumulation in individuals with normal weight.

## Patients and methods

A comprehensive description of the methods used in this study can be found in the supplementary data.

### Study sample

We used data from the UKB, which contains information from ∼500,000 participants aged between 40 and 69 years recruited from the UK between 2006 and 2010.^25^ The UKB includes health data, results from physical examinations, and biological samples for genetic analysis.

### Data quality control

**Fig. 1:**
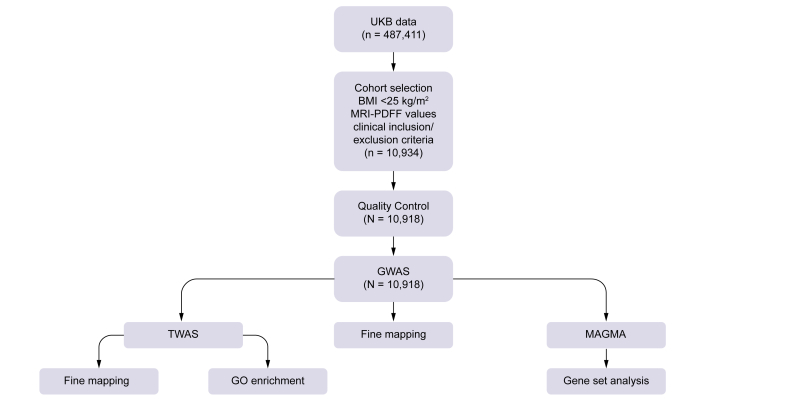
Analytical workflow and study design. The UKB imputed genotyped data version 3, encompassing 487,411 individuals and more than 96 million variants based on the GRCh37 genome build, was utilized for this study. Subsequent filtering and processing steps produced a final cohort comprising 10,918 individuals for the GWAS. The additional analyses following the GWAS were performed as described in the Patients and methods section. GWAS, genome-wide association study; UKB, UK Biobank.

An overview of the analytical workflow is shown in Fig. 1. The UKB Imputed Genotyped Data Version 3 was utilized for this study, and preprocessing, quality control, and genetic association analysis were performed using PLINK 2.0. Only variants with high imputation quality (imputed information score >0.8) and an autosomal location were included. Samples with: (1) heterozygosity outside of three SDs; (2) kinship value ≥0.125; and (3) discrepancies between UKB genetic and self-reported sex were excluded. To establish a homogenous ethnic group, we selected individuals with a self-reported ancestral background of ‘White British’, choosing ‘Caucasians’ from the genetic ethnic grouping. Outliers were removed using principal components, and only samples with unambiguous values for the phenotype and covariates used in the association analysis were included. We selected individuals with available MRI-PDFF values up until the date of the analysis and removed participants with conditions that might impact liver fat levels based on International Classification of Diseases Ninth and Tenth Revision (ICD-9 and ICD-10) codes (Table S1). After selecting participants within the normal BMI category (BMI <25 kg/m^2^), we achieved a sample size of 10,934 individuals.

We then excluded variants with a minor allele frequency ≤0.05, missingness per individual ≥0.05, missingness per marker ≥0.05, and Hardy–Weinberg equilibrium *p* ≤1x10^-6^, obtaining a final sample size of 10,918 individuals and a pool of 6,156,170 variants. The principal component analysis plot for this final dataset is shown in Fig. S1.

### GWAS

We conducted two distinct GWAS: one utilizing a case–control (CC) design, where MRI-PDFF values were used to define individuals with (≥5%) or without (<5%) hepatic steatosis, and the other, a QT design using the full range of MRI-PDFF values. Power analysis was conducted using the genpwr R-package, assuming a significance threshold of *p* <5.0x10^-^^8^, a logistic regression model, a minimum allele frequency (MAF) ≥5%, and odds ratio (OR) of 1.5 and 2. Both GWAS were adjusted for birth year, sex, BMI, alcohol intake frequency (UKB field 1,558), and the first 10 genetic principal components (UKB field 22,009). To account for multiple testing, we applied the genome-wide cutoff of *p* <5.0x10^-^^8^. Following both analyses, the ‘clump’ function was used to extract variants with a *p* <5.0x10^-^^8^ and variants in linkage disequilibrium (LD) with the most significant variants were removed. To investigate sex-specific effects, we conducted a GWAS using the genome-wide significant single nucleotide polymorphisms (SNPs), including sex as an interaction term in the additive model.

### GWAS fine mapping

Fine-mapping analysis was conducted on the significant SNPs using FINEMAP v.1.4, with the goal of identifying the causal variants. The algorithm (stochastic statistic search method) explores a set of the most probable causal configurations of the region. We considered a posterior inclusion probability (PIP) ≥80% as strong evidence of causality, a PIP ≥50% as moderate evidence, and a PIP <50% as weak evidence of causal association between variant and trait.

### MAGMA

We applied the multi-marker analysis of genomic annotation (MAGMA) method, which provides gene-level statistics using a multiple regression approach, to incorporate LD information between markers. The statistic was generated considering 10-kb regions surrounding the gene boundaries. Values of *p* were adjusted for multiple testing using the Bonferroni method, accounting for the number of genes tested. Results were investigated for Gene Ontology (GO) enrichment through the MAGMA gene-set analysis.

### TWAS

Liver expression data models from the Genotype-Tissue Expression Project version 8 (GTEx v8) were used to impute gene expression values, which quantify the relationship between individual genotypes and the corresponding gene expression levels, thereby capturing *cis*-acting genetic effects. TWAS *p* values were adjusted using the Bonferroni method based on the total number of genes included in the GTEx v8 reference (*p* <1.34x10^-05^; α = 0.05). Joint and conditional tests were conducted for all genes with suggestive adjusted *p* value (*p* <2.68x10^-05^; α = 0.10) to assess whether the signal in genome-wide significant genes was independent of variants located in nearby loci. To identify causal genes associated with the trait, we performed fine mapping using the FOCUS method.

## Results

### Genetic variants on chromosomes 19 and 22 are associated with hepatic steatosis in individuals with normal weight

Out of a total of 10,934 participants, 16 were excluded because of discrepancies between the filtered genetic data and associated covariates. The unfiltered dataset comprised 28,733,793 variants. From this dataset, 580,051 were excluded as a result of incomplete genotype data, 5,355 for Hardy–Weinberg disequilibrium, and 21,992,217 failed to meet the established MAF cut-off. In total, 6,156,170 common variants were retained for the GWAS analysis.

**Fig. 2:**
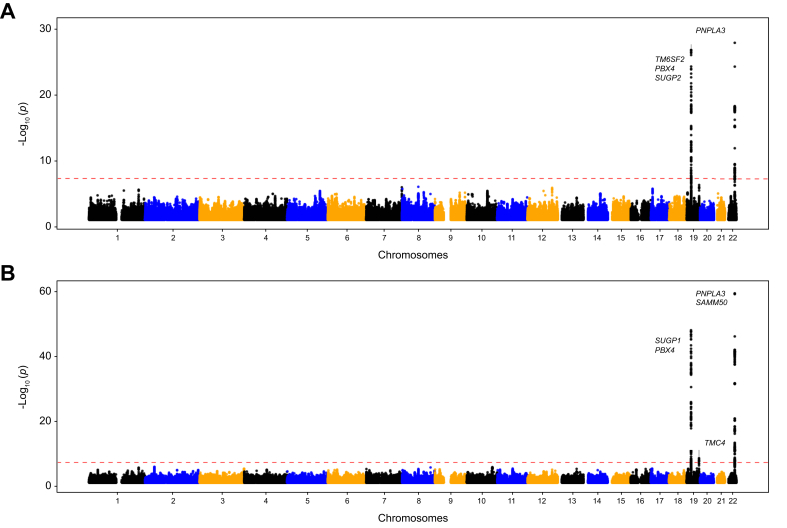
Manhattan plot showing the results for the CC-GWAS and QT-GWAS. The genes harboring variants associated with liver fat are shown for each analysis. *p* values were obtained using a logistic regression (CC-GWAS) and linear regression (QT-GWAS), adjusting for sex, birth year, and the top 10 principal components. The red line indicates the genome-wide significance level (*p* <5.0x10^-^^8^). CC case–control; GWAS, genome-wide association study; QT, quantitative trait.

CC-GWAS was conducted using 815 cases and 10,103 controls. Our power analysis supported the adequacy of the sample size for detecting significant associations at the genome-wide level (Fig. S2). Characteristics of the study participants are presented in Table 1. Individuals exhibiting elevated hepatic fat levels were significantly older, had a significantly higher BMI, and were more likely to be men when compared with those with normal liver fat levels. We identified 241 significant SNPs surpassing the genome-wide significance threshold of *p* <5.0x10^-^^8^ with minimal test statistics inflation (λ = 1.019) (Fig. S3A). These SNPs were clustered within two specific chromosomal regions on 19p12 and 22q13 (Fig. 2A and Table 2; Table S2) and distributed across 16 distinct genes within a range of genomic elements, including exons, introns, and untranslated regions (UTRs). On chromosome 19, the associated region spanned 689,559 bp ranging from 19,103,986 to 19,793,545, and harbored 15 genes. While the rs58542926 variant within the *TM6SF2* gene showed the strongest evidence for association in this locus, the regions exhibiting the highest significance were in closer proximity to the *SUGP1* and *HAPLN4* genes (Fig. S4A).

**Table 1:** Characteristics of study participants in the CC-GWAS.

Variable	Hepatic fat ≥5%	Hepatic <5%	*p* value
n	815	10,103	–
Men/Women	447/368	3,470/6,633	1.279E-31
Age, years	71.06 (7.37)	69.58 (7.53)	5.648E-08
Men	71.03 (7.78)	70.74 (7.75)	0.457
Women	71.09 (6.83)	68.98 (7.34)	1.927E-08
BMI, kg/m^2^	23.78 (1.07)	22.87 (1.55)	1.582E-92
Men	23.96 (0.91)	23.26 (1.34)	2.808E-41
Women	23.57 (1.20)	22.67 (1.62)	6.640E-36
MRI-PDFF, %	8.336 (3.974)	2.307 (0.803)	1.273E-213
Men	8.255 (3.864)	2.523 (0.824)	5.288E-115
Women	8.435 (4.107)	2.194 (0.768)	1.359E-97

All variables are expressed as mean values. Sex distribution was tested using chi-square, while the remaining parameters were assessed using *t* test. SD is shown in parentheses. CC, case–control; GWAS, genome-wide association study.

**Table 2:** Variants significantly associated with hepatic fat in the CC-GWAS∗.

Variant	Gene	Chromosome	Position	Effect allele	Case_Freq	Ctl_Freq	OR	SE	*p* value
rs738408	*PNPLA3*	22	44,324,730	T	0.335	0.212	1.92	0.057	5.35E-30
rs58542926	*TM6SF2*	19	19,379,549	T	0.145	0.069	2.37	0.079	1.48E-27
rs17217098	*PBX4*	19	19,702,384	A	0.126	0.063	2.22	0.084	1.22E-21
rs73006914	*SUGP2*	19	19,110,422	T	0.081	0.049	1.80	0.101	4.97E-09

∗ After linkage disequilibrium clumping. CC, case–control; GWAS, genome-wide association study; OR, odds ratio.

On chromosome 22, the region of association extended over 72,586 bp, from 44,324,558 to 44,397,144. This segment included genes such as *PNPLA3*, *SAMM50*, and *PARVB*. Of these, the SNP rs738408 in the *PNPLA3* gene exhibited the most significant association, with *p* = 5.4x10^-30^ (Fig. S4B). Sex-interaction analysis did not reveal any statistically significant SNPs (Table S3).

**Fig. 3:**
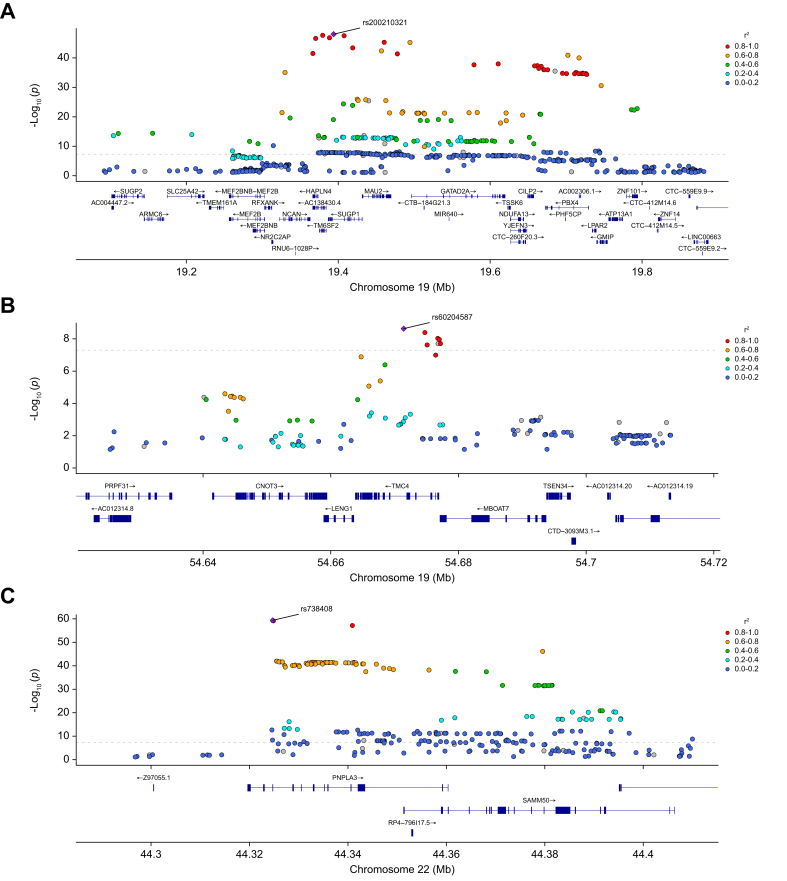
Regional plot showing the three significant loci from the QT-GWAS (*p* <5.0x10^-^^8^). (A) Region 1 on 19p13.11 (positions 19,103,986–19,793,545). (B) Region 2 on 19q13.4 (positions 54,671,421–54,677,189). (C) Region 3 on 22q13.31 (positions 44,324,558–44,409,993). For each region, the gene containing the most significant variant is depicted. *p* values were obtained using a linear regression (QT-GWAS), adjusting for sex, birth year, and the top 10 principal components. Linkage disequilibrium was estimated using PLINK2 (*—r* command). GWAS, genome-wide association study; QT, quantitative trait.

Characteristics of the study participants and summary values of the variables used in the QT-GWAS are shown in Table 3. We identified 418 significant SNPs predominantly clustered within three regions (two located on chromosome 19 and one on chromosome 22) and distributed across 24 distinct genes (Fig. 2B and Table 4; Table S4). Similar to the CC-GWAS, low inflation was observed in this analysis (λ = 1.046; Fig. S3B). The first region on chromosome 19 spanned 689,559 bp (positions 19,103,986–19,793,545; Fig. 3A) and was also detected in the CC-GWAS analysis. Within this region, rs200210321, located in the *SUGP1* gene, exhibited the strongest evidence of association. The second region on chromosome 19, which was not identified in the CC-GWAS, spanned 5,768 bp (positions 54,671,421–54,677,189; Fig. 3B), with the strongest association observed for rs60204587 in the *TMC4* (transmembrane channel like 4) gene.

**Table 3:** Characteristics of study participants in the QT-GWAS.

Variable	Value
n	10,918
Men/women	3,917/7,001
Age, years (SD)	69.69 (7.52)
Men	70.77 (7.75)
Women	69.09 (7.32)
BMI, kg/m^2^ (SD)	22.94 (1.54)
Men	23.34 (1.32)
Women	22.72 (1.61)
MRI-PDFF, % (SD)	2.757 (2.070)
Men	3.177 (2.372)
Women	2.522 (1.839)

All variables are expressed as mean values. SD is shown in parentheses. GWAS, genome-wide association study; QT, quantitative trait.

**Table 4:** Variants significantly associated with hepatic fat in the QT-GWAS∗.

Variant	Gene	Chromosome	Position	Effect allele	BETA	SE	*p* value
rs738408	*PNPLA3*	22	44,324,730	T	0.532	0.032	4.31E-60
rs200210321	*SUGP1*	19	19,393,890	AG	0.766	0.052	8.92E-49
rs17217098	*PBX4*	19	19,702,384	A	0.727	0.054	1.24E-41
rs73006914	*SUGP2*	19	19,110,422	T	0.487	0.062	4.64E-15
rs11912828	*PNPLA3; SAMM50*	22	44,348,116	A	-0.211	0.031	1.19E-11
rs12484530	*PARVB*	22	44,409,993	A	0.292	0.048	1.61E-09
rs60204587	*TMC4*	19	54,671,421	A	0.167	0.028	2.32E-09
rs12609436	*GMIP*	19	19,743,098	T	0.161	0.027	3.93E-09

∗ After linkage disequilibrium clumping. GWAS, genome-wide association study; QT, quantitative trait.

On chromosome 22, the region of association spanned 85,435 bp (positions 44,324,558–44,409,993; Fig. 3C), and the most significant SNP in this region was rs738408, located in the *PNPLA3* gene. This region was also identified in the CC-GWAS. Overall, 241 SNPs overlapped between the two analyses, with QT-GWAS capturing all those identified in the CC-GWAS and detecting an additional 177 variants (Table S5).

Using the genome-wide significant SNPs, we conducted a sex-interaction analysis. However, as in the CC- GWAS, no significant interactions with sex were detected after adjusting for multiple testing (Table S6).

### Fine-mapping analysis identifies three potential causal variants located in *GATAD2A*, *SUGP1*, and *MAU2*

We performed a fine-mapping analysis incorporating all genome-wide significant SNPs to identify potential causal variants within each locus. This analysis revealed three candidate variants: rs57009615 (*GATAD2A*, intron 11), rs2240117 (*SUGP1*, intron 3), and rs2285628 (*MAU2*, 3'-UTR). Each variant demonstrated strong evidence of a causal relationship with hepatic fat, as indicated by PIP = 1.000 (Table S7). All three SNPs exhibited combined annotation-dependent depletion (CADD) scores suggesting a low likelihood of being deleterious; specifically, the scores were 6.705 for rs57009615; 0.284 for rs2240117; and 5.211 for rs2285628. By contrast, the RegulomeDB scores, which are indicative of the potential regulatory nature of these variants, varied significantly: 73.5% for rs57009615; 18.4% for rs2240117; and 60.9% for rs2285628. The *GATAD2A* variant was classified with a high rank of ‘2b’ (supporting data includes transcription factor binding along with any motif presence, footprint evidence, and chromatin accessibility peaks), whereas the *SUGP1* and *MAU2* variants had low ranks of ‘7’ and ‘4’, respectively. Overall, only rs57009615 demonstrated a high probability of being a relevant regulatory variant.

### MAGMA identified *RFXANK,* a gene not detected in the GWAS

We performed MAGMA, utilizing summary statistics from both the CC-GWAS and QT-GWAS. CC-MAGMA identified eight genes (total genes tested = 18,042; adj *p* <2.77x10^-06^), all of which harbored variants with significant evidence of association in the CC-GWAS (Fig. S5A and Table S8A). In the QT-MAGMA, 19 genes were identified (total genes tested = 18,042; adj *p* <2.77x10^-06^). Of these, only one gene, *RFXANK*, represented a novel locus not previously detected in the QT-GWAS dataset (Fig. S5B and Table S8B).

MAGMA gene-set enrichment analysis did not reveal any significant GO functional classes (Fig. S6 and Table S9).

### TWAS and fine mapping identify *SAMM50* as causal gene for hepatic steatosis

**Fig. 4:**
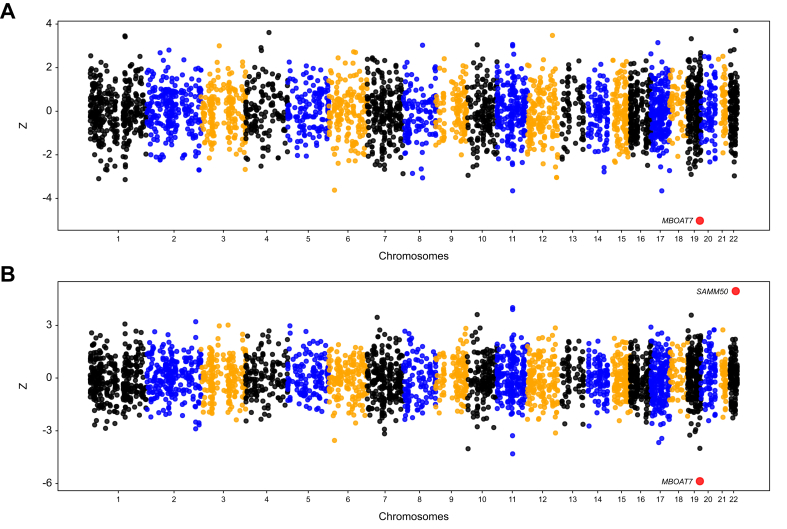
Miami plot illustrating the results of TWAS for the CC-GWAS and QT-GWAS. Genes exhibiting significant TWAS associations with liver fat, after applying Bonferroni correction accounting for the number of genes tested (n = 3,726; *p* <1.34x10^-05^) and passing the joint/conditional test, are represented by red dots. Fine-mapping analysis indicates strong evidence supporting *SAMM50* as a potential causal gene. CC case–control; GWAS, genome-wide association study; QT, quantitative trait; TWAS, transcriptome-wide association study.

The TWAS analysis, utilizing liver data models from GTEx v8 to investigate the relationship between genetic variants and gene expression levels, did not reveal any new associations beyond those identified in the GWAS or MAGMA. In the CC-TWAS, we identified one gene, *MBOAT7*, that remained significant after multiple testing correction (Z = -5.025; adj *p* = 1.9x10^-03^). This gene also passed joint and conditional testing, suggesting that its signal is independent of nearby SNPs (Fig. 4A; Table S10A). While significant genetic variants in *MBOAT7* were not identified in the CC-GWAS, they were detected in the QT-GWAS and further confirmed in the QT-MAGMA.

In the QT-TWAS, two genes, *MBOAT7* and *SAMM50,* emerged as significant, both passing the joint and conditional testing criteria (Fig. 4B; Table S10B)*.* Variants in the *SAMM50* gene were detected in the QT-GWAS (Table S4) and the QT-MAGMA, further substantiated the significance of this gene (Table S8B). TWAS fine mapping indicated a potential causal association with *SAMM50* (PIP = 1.000). By contrast, the evidence for *MBOAT7* was less definitive, with PIPs = 0.735 and 0.395 in the CC and QT analysis, respectively (Table S10).

Although we conducted GSEA using the TWAS-Z as the effect size, neither the CC nor the QT analyses identified significantly enriched GO functional classes (Table S11).

### Comparison with previous GWAS in ‘lean NAFLD’ identifies new variants

Two GWAS have previously investigated MASLD specifically in normal-weight individuals.^23^^,^^24^ One study, conducted in a Japanese population, did not achieve genome-wide significance for any SNPs after adjusting for sex and principal components.^23^ By contrast, the other study identified genome-wide significant signals at many loci.^24^ Our study overlapped with these findings, identifying 137 shared genetic variants from both the CC- and QT-GWAS (Tables S12 and S13). However, our CC-GWAS revealed 104 genetic variants not detected in the previous GWAS (Table S14), whereas the QT-GWAS identified 281 unique variants (Table S15). Some of these variants were found in *ARMC6* (armadillo repeat containing 6), *CILP2* (cartilage intermediate layer protein 2), *MAU2*, *MBOAT7*, *NR2C2AP* (nuclear receptor 2C2 associated protein), *PARVB* (parvin beta), *SLC25A42* (solute carrier family 25 member 42), *SUGP2*, *TM6SF2*, and *TMC4* (transmembrane channel like 4).

## Discussion

Our study identified significant genetic associations on chromosomes 19 and 22 linked to hepatic steatosis in individuals with normal BMI. These findings challenge the prevailing view that genetic risk factors for MASLD primarily manifest in individuals with excess weight. The identification of variants in well-established genes, such as *PNPLA3*, *SAMM50*, and *TM6SF2*, underscores the role of genetic predisposition in the development of MASLD in normal-weight individuals. These associations were robust across both CC-GWAS and QT-GWAS designs, further strengthening the relevance of these loci. In addition, GWAS fine mapping identified potential causal variants in *GATAD2A*, *SUGP1*, and *MAU2,* while TWAS fine mapping implicated *SAMM50* as a driver of steatosis in normal-weight individuals, possibly through changes in gene expression.

Our findings provide new insights into genetic determinants of steatotic liver in individuals outside the typical clinical MASLD profile, emphasizing the heterogeneity and complexity of the disease beyond its association with obesity. Notably, recent research has identified two distinct MASLD subtypes, supported by transcriptomic and metabolomic data: a liver-specific form and a cardiometabolic form.^26^ The cardiometabolic subtype is characterized by dysglycemia, elevated triglycerides, and heightened risks of cardiovascular disease and diabetes. These findings support the importance of developing tailored therapeutic strategies to address the diverse pathways underlying MASLD.

While interest in the genetic susceptibility to hepatic steatosis in non-obese individuals is not a new line of investigation, most previous studies focused primarily on candidate genes, often yielding associations with modest effect size and limited genome-wide significance (^27^ and references therein). To date, only two studies have conducted genome-wide association investigations in normal-weight populations. One of these studies, conducted in a Japanese population, did not find any variants reaching genome-wide significance.^23^ The sample size of 275 individuals with MASLD and 1,411 non-MASLD controls may have had limited the power to detect genetic association. By contrast, the other study, which also utilized the UKB resource, identified genome-wide significant associations, including some that overlapped with the results reported here.^24^ When comparing our results with those of the previous study,^24^ we observed association with variants in 11 genes, including *PNPLA3*, *SAMM50*, and *SUGP1*. However, we also identified new associations with genetic variants in *ARMC6*, *CILP2*, *MAU2*, *MBOAT7*, *NR2C2AP*, *PARVB*, *SLC25A42*, *SUGP2*, *TM6SF2*, and *TMC4* that were absent in the previous study. These novel associations could reflect differences in the specific populations studied, methodologies used, or the nature of hepatic steatosis in non-obese individuals. Surprisingly, we did not replicate the signal in *HFE*, which had been the primary finding from the earlier study.^24^ The lead variant in *HFE* identified in that study (rs1800562) was detected with *p* = 0.052 and *p* = 0.035 in our QT and CC-GWAS, respectively. The discrepancy in findings might be partially attributable to different sample selection criteria, because the previous GWAS utilized 12,804 controls, compared with 10,103 in the current study. Furthermore, we excluded individuals with disorders of mineral metabolism, which may have removed individuals carrying *HFE* variants. Unlike the prior work, we also assessed liver fat content as a QT, which strengthened the evidence of association, and performed several post-GWAS analyses that resulted in detection of potentially causal variants.

The *PNPLA3* SNP rs738409, the second strongest signal in our study, has been extensively linked to hepatic steatosis, steatohepatitis, fibrosis, cirrhosis, and hepatocellular carcinoma across diverse populations,[28], [29], [30] particularly in individuals with obesity.[31], [32], [33], [34], [35] The variant was also identified in the GWAS by Sun *et al.*^24^ (*p* = 8.6x10^-13^), suggesting a broader role in hepatic fat accumulation beyond obesity-related steatotic liver disease and independent of adiposity. The rs738409 variant is non-synonymous, leading to an isoleucine-to-methionine substitution at position 148 (I148M), which impairs triglyceride mobilization and promotes hepatic fat accumulation.^36^

Cherubini *et al.*^37^ recently reported a sex-specific association between the *PNPLA3* p.I148M variant and steatotic liver disease. While women are generally protected from MASLD during their reproductive years, some experience rapidly progressive disease following menopause.^38^ Their study highlighted a significant interaction between female sex and the p.I148M variant, markedly increasing the risk of steatosis, fibrosis, and advanced liver complications. In individuals with obesity, hepatic *PNPLA3* expression was notably higher in women than in men, and correlated with estrogen levels. Mechanistic investigations revealed that *PNPLA3* expression is upregulated by estrogen receptor-α (ER-α) agonists via an ER-α-binding site within a *PNPLA3* enhancer. CRISPR–Cas9 experiments further demonstrated that this interaction drives lipid accumulation and fibrosis, establishing a direct link between ER-α and *PNPLA3* p.I148M in the pathogenesis of fatty liver disease in women. Considering these findings, we assessed sex-specific effects. However, these analyses failed to detect significant interactions with sex in our dataset.

*SAMM50* (Sorting and Assembly Machinery Component 50 Homolog) encodes a mitochondrial protein involved in maintaining mitochondrial morphology and promoting mitophagy, both of which are essential for mitigating the effects of oxidative stress in liver cells.^39^^,^^40^ The identification of *SAMM50* as a significant gene through our GWAS and TWAS analyses, along with its designation as a primary driver based on fine mapping, supports the critical role of mitochondrial dysfunction and oxidative stress in the pathogenesis of liver fat accumulation in individuals with normal weight. Our findings are concordant with those of Li *et al.*,^41^ who reported increased MASLD susceptibility in Chinese individuals with overweight (BMI >23 kg/m^2^) carrying risk genotypes at rs738491 and rs2073082. Higher hepatic *SAMM50* transcript levels were observed in patients with MASLD and in lipid-loaded Hep3B cells.^41^ Interestingly, *in vitro* experiments demonstrated downregulation of *SAMM50* by rs738491 and rs2073082 variants, resulting in impaired fatty acid oxidation and subsequent accumulation of lipid.^41^ Conversely, *SAMM50* overexpression was found to enhance fatty acid oxidation and reduce lipid buildup, indicating that its deficiency directly contributes to the accumulation of lipids by limiting fatty acid breakdown. Our findings extend the association of *SAMM50* variants to normal-weight individuals and provide further evidence supporting a causal role for *SAMM50* in hepatic steatosis.

GWAS fine mapping revealed the presence of potential causal variants in *SUGP1*, *GATAD2A*, and *MAU2;* all these genes also emerged in the previous analysis.^24^ *SUGP1* contributes to the regulation of cholesterol metabolism.^42^ An extended haplotype including rs10401969, significant in our CC-GWAS and QT-GWAS, as well as the previous GWAS,^24^ has been linked with coronary artery disease, plasma LDL cholesterol levels, and diverse energy metabolism phenotypes (^42^ and references therein). In addition, we previously observed a nominal association between rs10401969 and hepatic fat in individuals belonging to the severely obese BMI category (BMI >40 kg/m^2^) (*p* = 3.1x10^−07^).^43^ Although the functional consequences of rs10401969, an intronic variant, are unknown, some evidence suggests that it impacts alternative splicing^42^ of the *SUGP1* transcript.

*GATAD2A* (GATA Zinc Finger Domain Containing 2A) encodes a protein that enables zinc ion binding. Low serum zinc levels have been observed in MASLD.^44^ Zinc, the homeostasis of which is primarily regulated in the liver, is associated with hepatic steatosis through various mechanisms, including antioxidant defense, insulin resistance, inflammation, and fibrogenesis.[45], [46], [47] While zinc supplementation may reduce fibrosis levels, it had little effect on IHTG in mouse models of MASH.^48^ The potentially causal variant identified in our analysis, rs57009615, although having an intronic location, is predicted by RegulomeDB analysis to have regulatory and functional effects. Further studies are warranted to explore the effect of this variant on *GATAD2A* RNA processing and expression.

*MAU2* (MAU2 Sister Chromatid Cohesion Factor), the remaining gene identified in our fine-mapping analysis, encodes a protein involved in sister chromatid cohesion during cell division. Currently, there is limited mechanistic evidence linking MAU2 to hepatic steatosis or metabolic dysfunction.

Although the present findings offer important insights, several limitations related to the study sample warrant consideration. First, although our sample size was substantial, the relatively small number of cases (n = 815) compared with controls might have limited our power to detect associations with smaller effect sizes. Nonetheless, this sample size is comparable to that used by Sun *et al.*,^24^ who reported significant associations at four loci, and exceeds that of Yoshida *et al.*^23^ Second, our analysis was restricted to individuals of European ancestry, which limits the generalizability of our findings to other populations. Although several of the identified variants have been reported across diverse ancestries, the prevalence and genetic architecture of MASLD differ globally, emphasizing the need for replication in more heterogenous cohorts. Furthermore, while we applied rigorous methods to control for population stratification, residual confounding cannot be entirely excluded. Finally, we acknowledge the absence of an independent replication cohort, a limitation driven by the current lack of large, well-phenotyped normal-weight MASLD datasets across ancestries, an essential resource for validating genetic findings in this understudied subgroup.

Beyond limitations related to the study sample, our findings also raise questions about the biological mechanisms underlying the identified associations, which remain unclear and require functional validation. Previous work by Li *et al.*^41^ suggests that *SAMM50* variants reduce its expression, leading to impaired fatty acid oxidation and lipid accumulation, mechanisms that could be relevant to our findings. In addition, MRI-PDFF was assessed at a single time point, limiting our ability to capture the dynamic nature of hepatic fat accumulation or distinguish between different causes of steatosis. This might introduce phenotypic heterogeneity and obscure associations specific to individuals with normal BMI. Given that this was a genetic analysis, environmental or lifestyle factors, such as diet and physical activity, were not considered, which might contribute to residual confounding.

Finally, although the UKB is an invaluable resource, its extensive use in MASLD studies raises the question of whether additional GWAS in this cohort are necessary. We believe that further GWAS using the UKB remain crucial for a few reasons. First, the large, well-phenotyped nature of the cohort allows for more precise identification of genetic variants, particularly those that might not have been fully elucidated in previous studies. Previous UKB-based GWAS[49], [50], [51], [52] have been instrumental in identifying loci that shape MASLD susceptibility across diverse contexts. However, these studies predominantly comprise individuals who are overweight or obese, potentially missing loci that are specific to hepatic steatosis in normal-weight individuals or independent of excess adiposity. By focusing on normal-weight individuals, our study identified novel loci, such as *ARMC6*, *TMC4*, and *SUGP2*, which were absent in the previous GWAS,^24^ while corroborating well-established signals at *PNPLA3* and *TM6SF2*. These findings suggest that unique genetic and environmental factors contribute to hepatic steatosis in normal-weight populations, underscoring the importance of targeted GWAS in refining our understanding of the MASLD heterogeneity.

The continued exploration of the genetic data in UKB will help to expand our understanding of MASLD, particularly in normal-weight individuals, and improve our ability to detect variants with smaller effects. Further analyses also have the potential to incorporate environmental or lifestyle factors, such as diet or exercise, which might influence hepatic steatosis. Thus, we believe that the UKB remains an important resource for exploring the complex genetic architecture underlying MASLD and its manifestations in non-obese individuals.

In conclusion, our study represents a significant step forward in understanding the genetic basis of hepatic steatosis in normal-weight individuals. The identification of novel genetic determinants, as well as the confirmation of previously detected associations, provides new insights into the pathophysiology of MASLD in this understudied population. As research into the genetic drivers of MASLD continues to evolve, our findings underscore the importance of considering genetic predisposition in the context of normal BMI, which might have distinct mechanisms and therapeutic needs compared with the broader MASLD population.

## Abbreviations

CC, case–control; ER estrogen receptor; IHTG, intrahepatic triglyceride; GO, Gene Ontology; GTEx v8, Genotype-Tissue Expression Project version 8; GWAS, genome-wide association study; LD, linkage disequilibrium; MAGMA, multi-marker analysis of genomic annotation; MASH, metabolic dysfunction-associated steatohepatitis; MASLD, metabolic-associated steatotic liver disease; MRI-PDFF, magnetic resonance imaging-proton density fat fraction; OR, odds ratio; PIP, posterior inclusion probability; QT, quantitative trait; SNP, single nucleotide polymorphism; TWAS, transcriptome-wide association study; UTR, untranslated region; UKB, UK Biobank.

## Financial support

This research was supported by the 10.13039/100000062NIDDK (R01DK127015).

## Authors’ contributions

Conceptualization of the work: JKD. Data collection: ISP, JD. Data analysis: ISP, JD. Data interpretation: ISP, JD, JKD. Project administration: JKD. Funding acquisition: JKD. Drafting of manuscript: ISP, JKD. Critical review of the manuscript: JD, NJS. Final approval of the version to be published: JKD.

## Data availability statement

GWAS summary statistics are available upon request from the corresponding author.

## Conflicts of interest

The authors did not receive any financial support to produce this manuscript.

Please refer to the accompanying ICMJE disclosure forms for further details.
