# Supplementary material for: Genetic variants influencing liver fat in normal-weight individuals of European ancestry
Source: JHEP Rep. 2025 May 14;7(8):101453. doi: 10.1016/j.jhepr.2025.101453 (PMC12270618; doi:10.1016/j.jhepr.2025.101453)
Supplement: Multimedia component 1 [file mmc1.pdf]

Potential causal links between genetic variants in *SAMM50*, *SUGP1*, *MAU2*, and *GATAD2A* and liver fat in individuals with normal weight

Ignazio S. Piras, Janith Don, Nicholas J. Schork, Johanna K. DiStefano

Table of contents

Supplementary methods……………………………………………………………………..2

Supplementary figures…………………………………………………………….............8

Supplementary tables………………………………………………………….............14

Supplementary references……………………………………………..........................76

Supplementary methods

Study sample. Data used in the following analyses was derived from the UKB database, which contains information from approximately 500,000 participants aged between 40 and 69 years recruited from the United Kingdom between 2006 and 2010 [1]. The UKB database includes comprehensive health data, results from physical examinations, and biological samples for genetic analysis.

Data preprocessing, filtering, and quality control. An overview of the analytical workflow and study design is shown in **Fig 1**. The UKB imputed genotyped data version 3, encompassing 487,411 individuals and more than 96 million variants based on the GRCh37 genome build, was utilized for this study. Preprocessing, quality control, and genetic association analysis were performed using PLINK 2.0. Initially, we converted UKB bgen format files to PLINK bed/bim/fam format. During this conversion, we filtered the dataset to include only variants with high imputation quality, specifically utilizing the criterion of an ‘imputed information score (defined by UKB)’ > 0.8. Variants were restricted to those found on autosomal chromosomes. Consequently, this preprocessing step resulted in a dataset of 28,733,793 variants and 487,409 samples. Next, we applied quality control filters. Samples with heterozygosity values that were outside of three standard deviations were excluded, resulting in a sample size of 474,828 individuals. We also removed samples with a kinship value ≥ 0.125 based on UKB genetic relatedness data. Individuals who had withdrawn their consent up until the last update (April 25, 2023) prior to the analysis were excluded. To ensure consistency, we cross-referenced the UKB

genetic sex (UKB data field 22001) and self-reported sex (UKB data field 31) and retained samples with consistent sex information across both fields. To establish a homogenous ethnic group and minimize confounding effects stemming from ancestry and environmental factors, we selected individuals with a self-reported ancestral background of ‘White British’ (UKB data field 21000) and refined the selection by choosing ‘Caucasians’ from the genetic ethnic grouping (UKB data field 22006), which constitutes the largest single ethnic group comprising > 80% of the samples. This step also involved removing outliers based on genetic principal components. In addition to these filters, we restricted the sample to individuals with unambiguous values for the phenotype and covariates used in the subsequent association analysis. For the current study, we selected individuals from the UKB database with available MRI-PDFF values (recorded in UKB field 40061-2.0) up until the date of the analysis, when 40,532 participants from the entire UKB cohort had these measurements. From this group, only participants who met the quality control criteria described above were selected. To exclude additional potential confounding factors, we identified participants with conditions that might impact liver fat levels using ICD9 and ICD 10 codes within the UKB diagnostic and death records (**Table S1**) and removed them from the selected group. When we limited selection to participants classified within the normal BMI category, defined as BMI < 25 kg/m^2, we achieved a sample size of 10,934 individuals.

For the association analyses described in the next section, we performed additional filtering using specific PLINK parameters. These included a minor allele frequency of 0.05, missingness per individual of 0.05, missingness per marker of 0.05, and Hardy-Weinberg equilibrium of 1E-6. The application of these filtering criteria resulted

in a reduction of the sample size to 10,918 individuals and a total of 6,156,170 variants. The principal component analysis plot utilizing this final dataset is shown in **Fig S1**.

Power analysis. Power analysis was conducted using the *genpwr R-package*, assuming a significance threshold of $p < 5.0 \times 10^{-08}$, a logistic regression model, a minimum allele frequency (MAF) ≥ 5%, and Odds Ratio (OR) of 1.5 and 2.

Genetic association analysis. We conducted two distinct GWAS. The first analysis utilized a case-control study design (CC), where MRI-PDFF values were used to define individuals with or without hepatic steatosis. The second analysis was a quantitative study (QT), directly using the range of MRI-PDFF values. Both studies employed the same filtering parameters, as described in the previous section, and utilized the same set of covariates, including birth year, sex, BMI, alcohol intake frequency (UKB field 1558), and the first 10 genetic principal components (UKB field 22009). To ensure consistency and comparability, we standardized each covariate using the 'covar-variance-standardize' function in PLINK [2]. For the association analyses, we employed the 'glm' function in PLINK, allowing us to assess the genetic associations while accounting for the specified covariates.

For the CC study (CC-GWAS), we defined hepatic steatosis as MRI-PDFF ≥5% (cases) and normal liver as MRI-PDFF <5% (controls). Using PLINK, we calculated odds ratios (OR) and P-values for each variant, adjusting for the specified covariates. To account for multiple testing, we applied the established genome-wide cutoff of p < 5.0E-08. To analyze MRI-PDFF in the QT analysis (QT-GWAS), we used the same 'glm' function in PLINK, but with continuous values of MRI-PDFF in the phenotype field. In this case, the linear model provided effect values (beta) and P-values for each variant. We

used the same parameters and covariates as in the CC-GWAS analysis and accounted for multiple testing using the same cutoff. Following both analyses, we employed the ‘clump’ function in PLINK 1.9 to extract variants with a P-value < 5.0E-08 and removed variants that were in linkage disequilibrium (LD) with the most significant variants. The clumping parameters used were clump-p1 = 5.0E-08, clump-r2 = 0.1, clump-kb = 250. SNPs were annotated with their corresponding genes utilizing the ANNOVAR software tool, referencing the human genome assembly version hg19. Finally, to investigate sex-specific effects, we conducted a GWAS using the genome-wide significant SNPs, including sex as an interaction term in the additive model. The results were adjusted using the Bonferroni method, accounting for the number of independent SNPs determined by eigenvalue decomposition of the Linkage Disequilibrium matrix, as described in Li et al. [3]

GWAS fine-mapping. We conducted a fine-mapping analysis with the goal of identifying the causal variants. The analysis was performed using the *FINEMAP v.1.4* software, which employs an algorithm that explores a set of the most probable causal configurations of the region. For both the CC and QT analyses, we selected all SNPs significant at the genome-wide level of p < 5.0E-08. The LD correlation matrix was estimated using *PLINK2* with the “--r square” function. Fine mapping was carried out using the *stochastic statistic search* method, considering the value of a posterior inclusion probability (PIP) > 80% as strong evidence of causality, a PIP ≥ 50% as moderate evidence of causality, and a PIP < 50% as weak evidence of causal association between the variant and the trait. We also utilized the Log_{10} Bayes Factor (LBF), which indicates the strength of association between a variant and the trait of interest without inferring causality. A LBF ≥ 2 implies strong evidence of association, while 1 < LBF ≤ 2 indicates moderate evidence for association,

0.5 < LBF ≤ 1 indicates suggestive evidence, and LBF ≤ 0.5 indicates weak evidence of association.

MAGMA. GWAS summary statistics were analyzed with the Multi-marker Analysis of GenoMic Annotation (MAGMA) method, which provides gene-level statistics using a multiple regression approach to incorporate LD information between markers and detect multi-marker effects. The statistic was generated considering 10 kb regions surrounding the gene boundaries (parameter *--annotate window=10,10*). P-values were adjusted for multiple testing using the Bonferroni method, accounting for the number of genes tested. Results were investigated for Gene Ontology enrichment through the MAGMA gene-set analysis.

TWAS. We utilized liver expression data models from the Genotype-Tissue Expression Project version 8 (GTEx v8) –European reference data – to impute gene expression values, as implemented in the FUSION software. These values quantify the relationship between individual genotype and corresponding gene expression levels, capturing *cis*-acting genetic effects. We matched genetic variants using “rsID” rather than genomic position to avoid issues arising from differences in genomic coordinates between GTEx v8 (GrCh38) and the GWAS dataset. Using GTEx v8 data, we computed gene weights to estimate the effects of individual SNPs on gene expression, which were then used to evaluate whether the predicted levels were associated with the phenotype. TWAS p-values were adjusted using the Bonferroni method based on the total number of genes included in the GTEx v8 liver reference (n = 3,726; $p < 1.34e-05$; alpha = 0.05). Joint and conditional tests were conducted for all genes with suggestive adjusted p-value (p <

2.68e-05; alpha = 0.10), to assess whether the signal in genome-wide significant genes was independent of variants located in nearby loci. Genes that exhibit joint significance are independent of variants located in adjacent loci, while genes classified as conditionally non-significant lose significance upon adjustment for the contributions of nearby genes. To identify causal genes associated with the trait, we performed TWAS fine mapping using the FOCUS method, which allows derivation of the covariance structure among TWAS statistics by integrating the LD and eQTL weights utilized in the prediction. The TWAS association statistics for all genes within a risk region were modeled using a multivariate Gaussian distribution, which is parameterized by effect sizes at causal genes, residual SNP effects, and the correlation structure induced by inferred expression weights in conjunction with LD. To mitigate potential bias arising from pleiotropic effects of SNPs, we incorporated an intercept term that quantifies the average SNP effect sizes associated with predicted expression.

Supplementary Figures

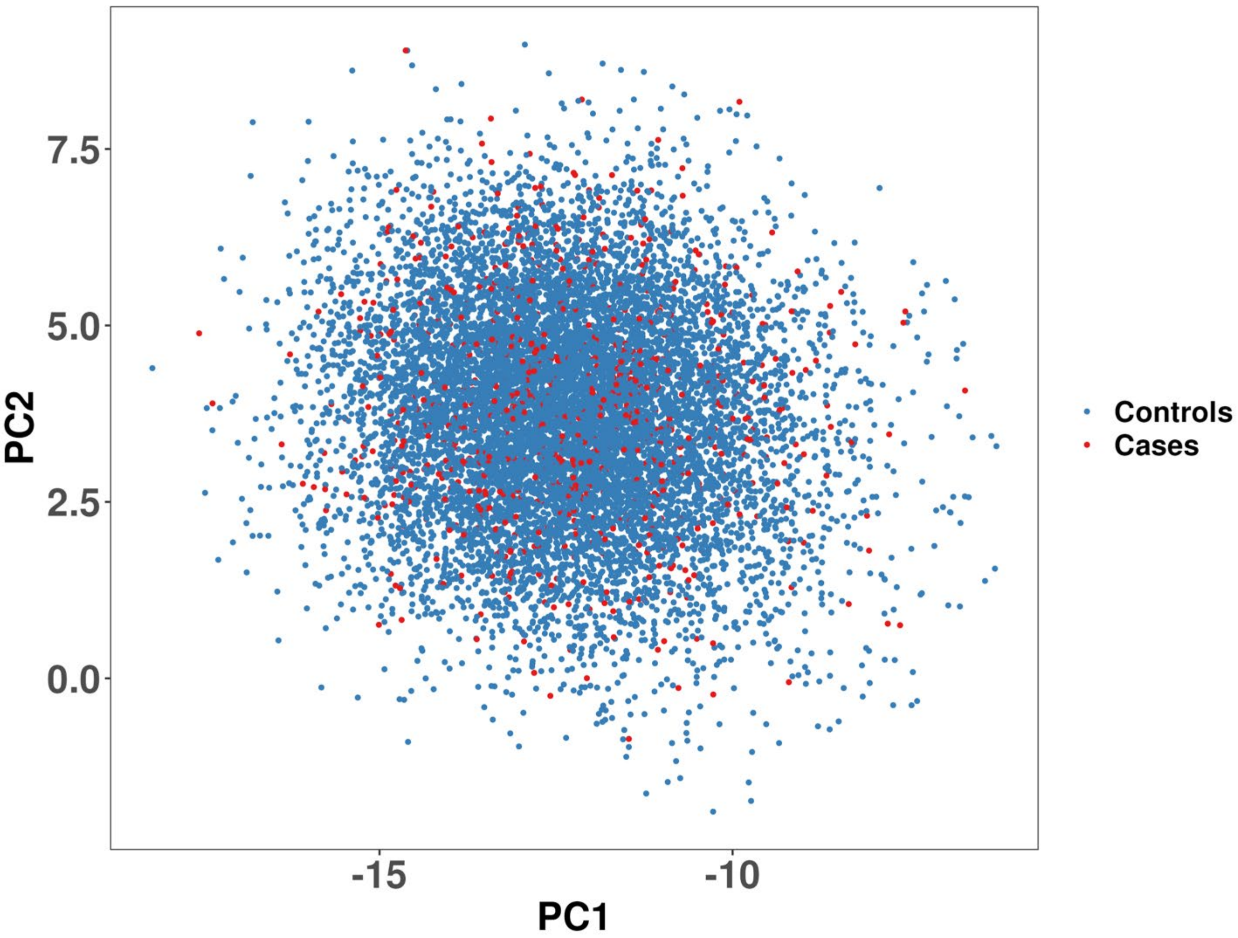

Fig S1. Principal Component Analysis conducted using the final dataset (6,156,170 SNPs; 815 cases and 10,103 controls). We used the pre-computed Principal Components available in the dataset (UKB field 22009).

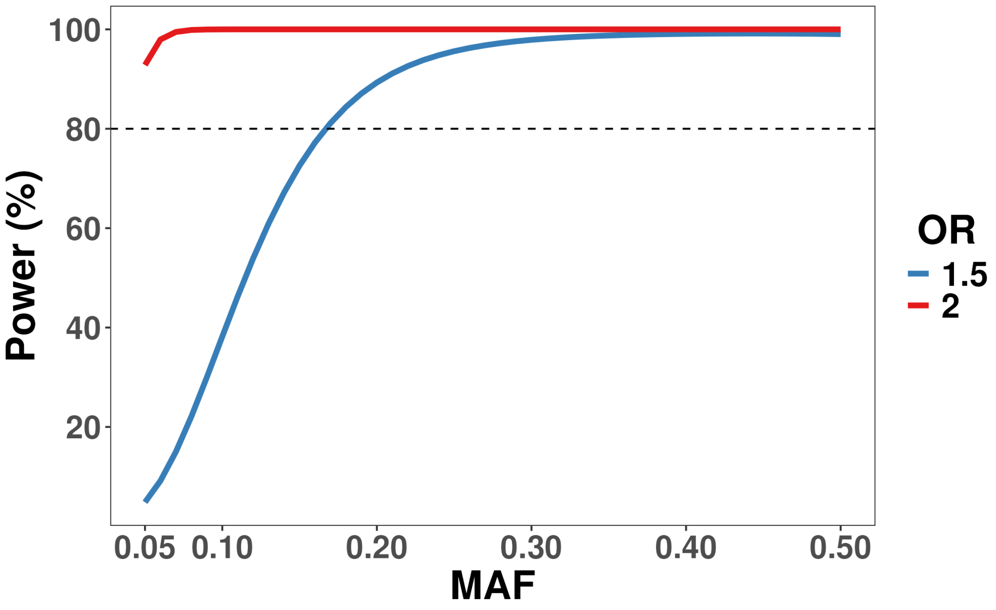

Fig S2. Power analysis was conducted using the *genpwr* R-package assuming 815 patients and 10,103 controls, a significance threshold of p < 5.0 x 10-08, a logistic regression model, and a minimum allele frequency (MAF) ≥ 5%. For OR = 1.5, we achieved 80% power to detect variants with a MAF ≥ 0.17, and for OR = 2, 92% power to detect variants with a MAF ≥ 0.05.

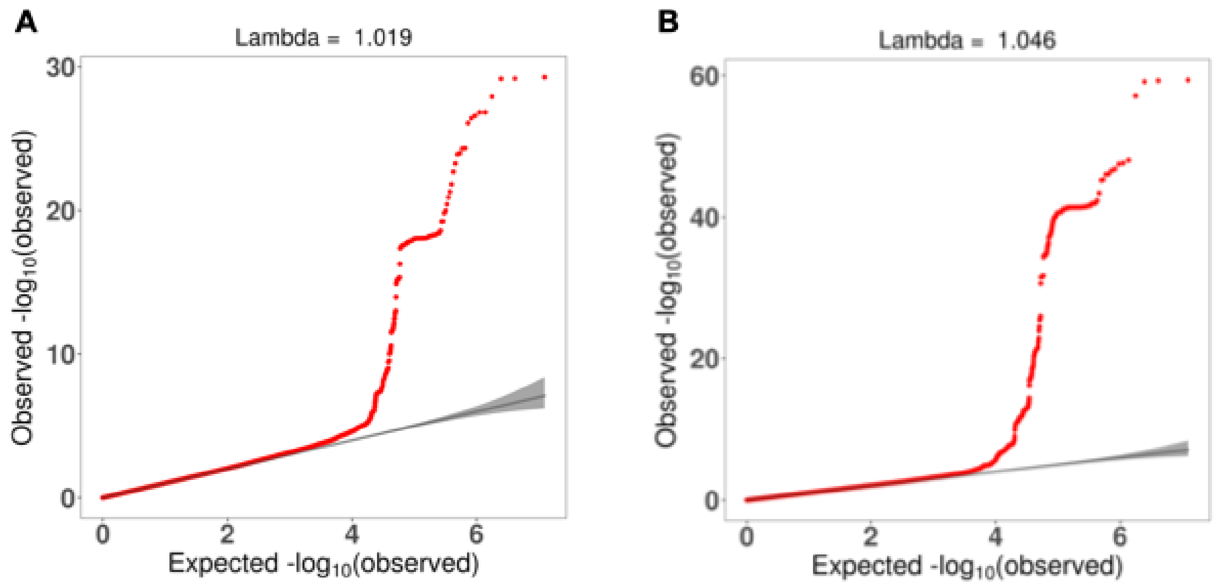

Fig S3. Quantile-Quantile plot showing the p-value distribution in the (A) case-control GWAS and (B) quantitative GWAS.

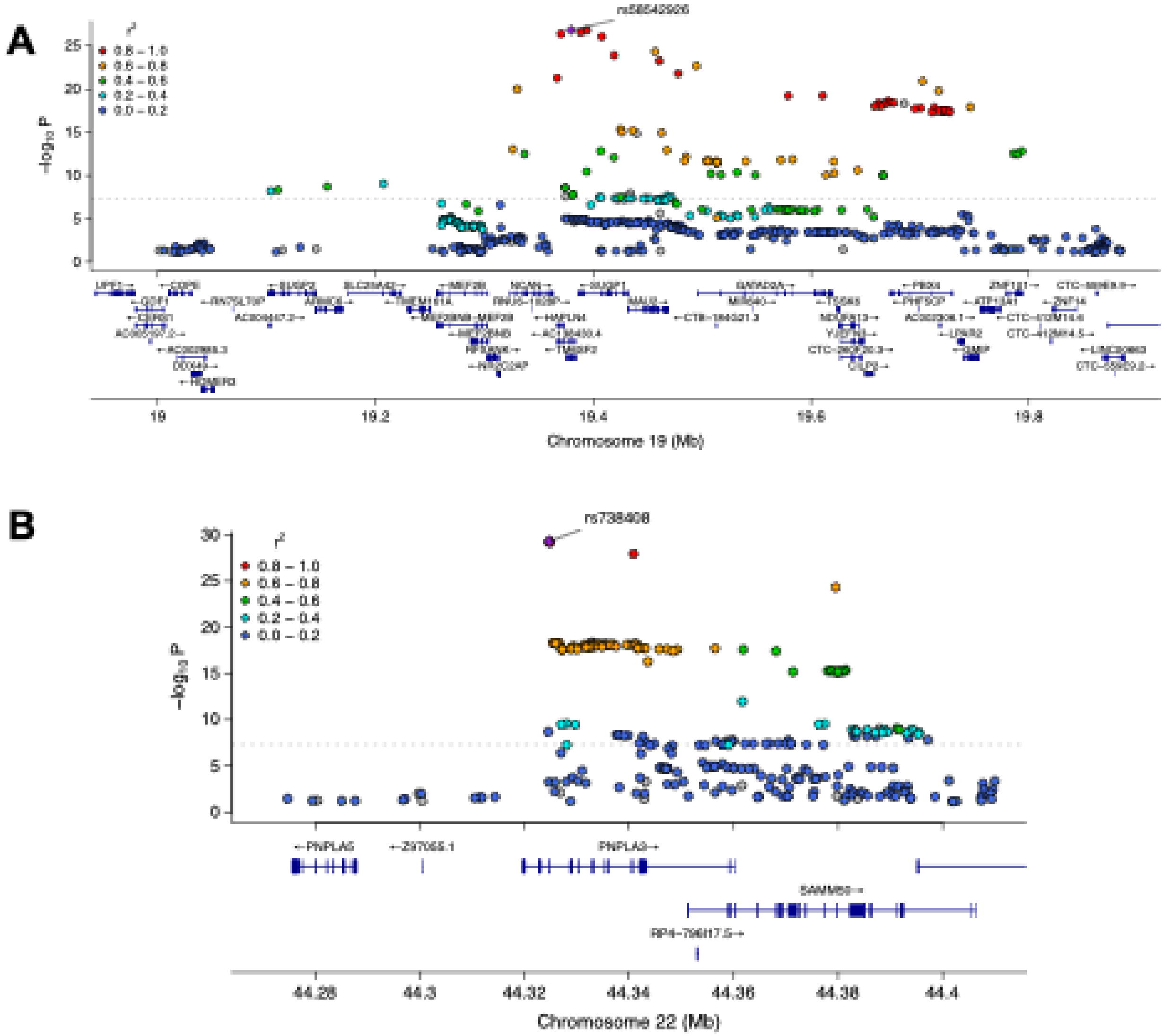

Fig. S4. Regional plot showing the significant regions identified in the case-control GWAS. LD information was estimated from the data using PLINK2.

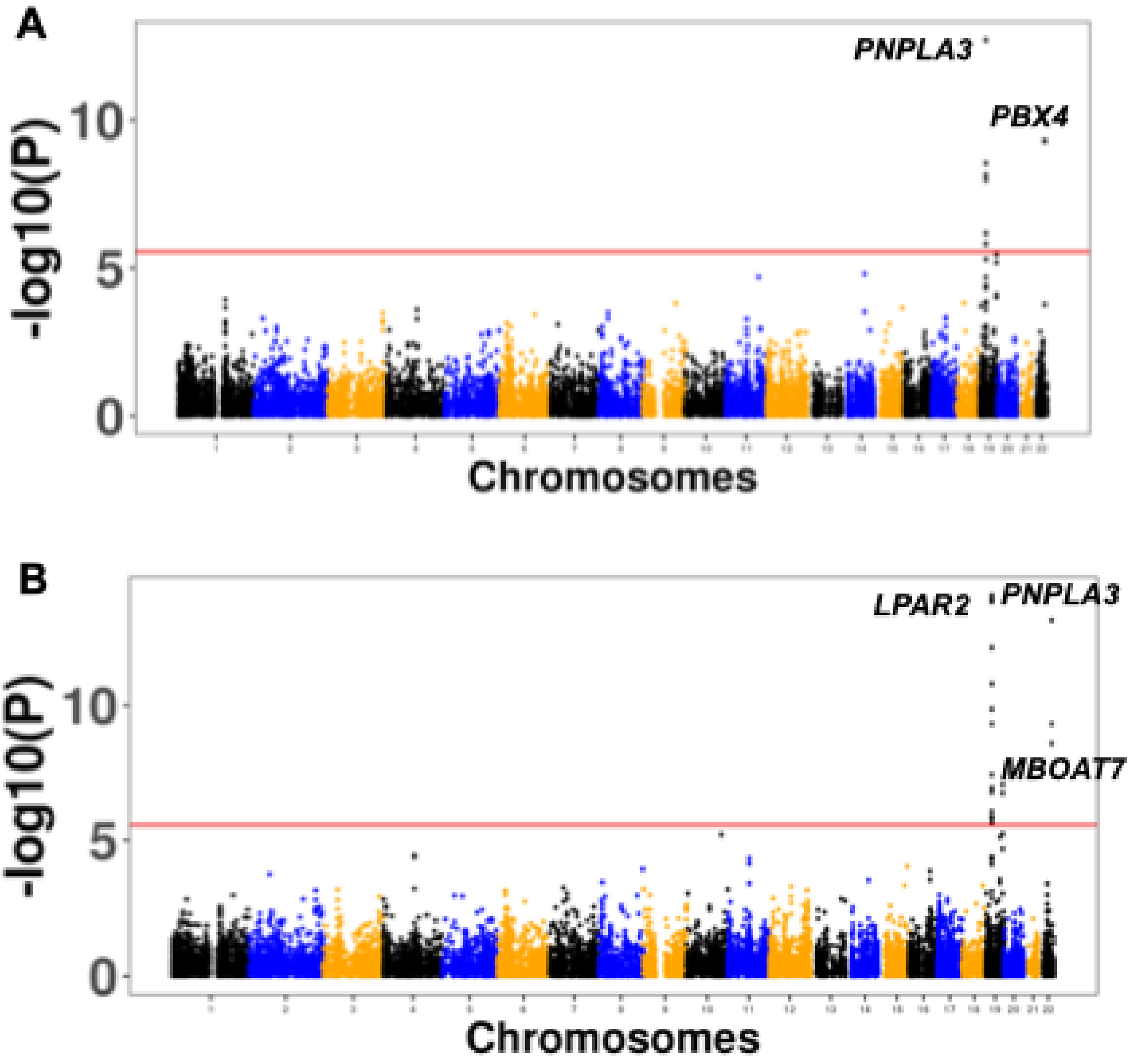

Fig S5. Results from the MAGMA analysis using the (A) CC-GWAS and (B) QT-GWAS. Only the top genes for each associated region are shown.

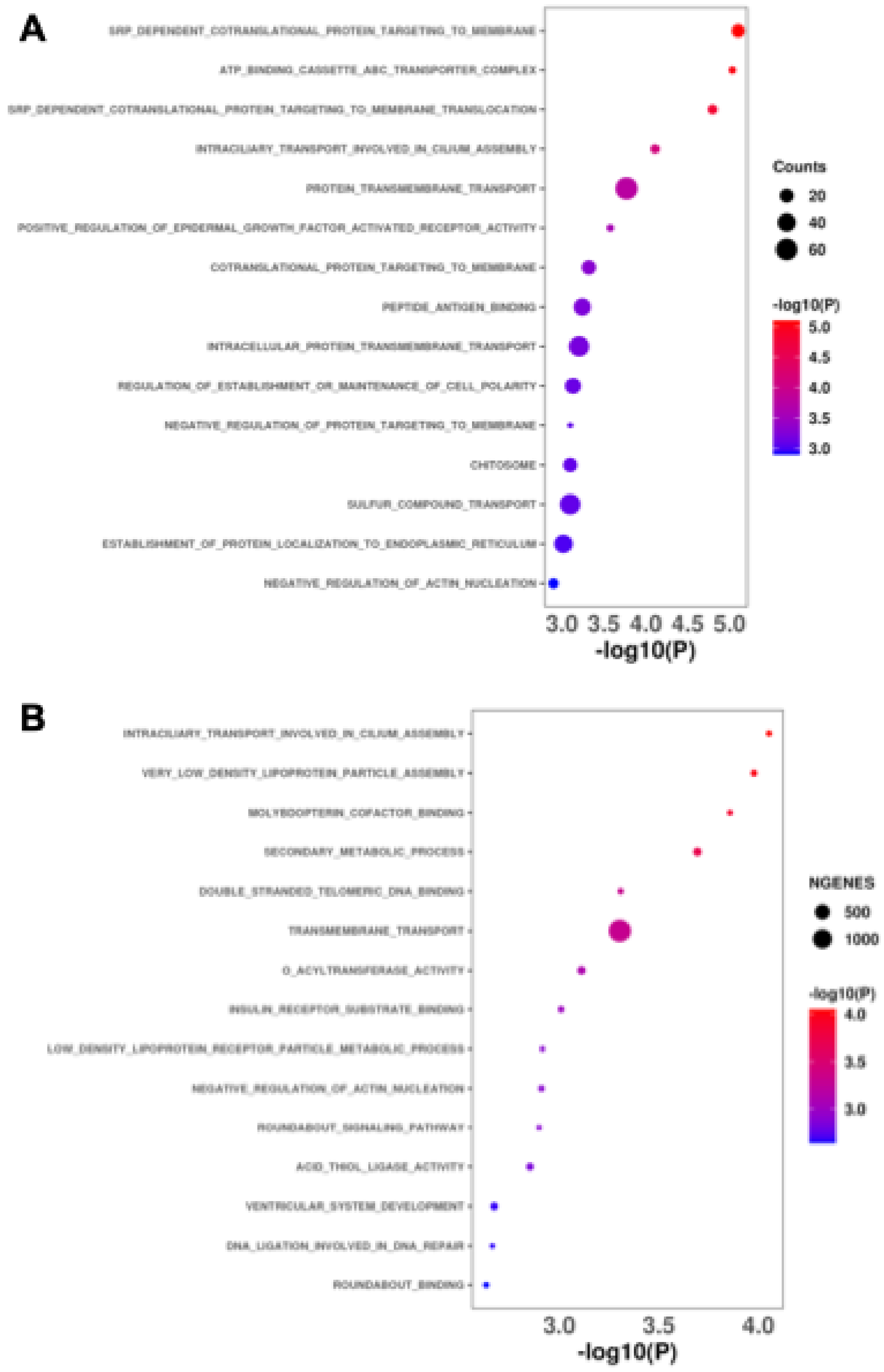

Fig. S6. MAGMA gene set analysis using the CC-MAGMA (A) and QT-MAGMA (B) results.

Supplementary Tables

Table S1A. ICD9 codes used to exclude participants from the study.

ICD9	UKB description
**5710**	5710 Alcoholic fatty liver
**5711**	5711 Acute alcoholic hepatitis
**5712**	5712 Alcoholic cirrhosis of liver
**5713**	5713 Alcoholic liver damage, unspecified
**70**	070 Viral hepatitis
**700**	0700 Viral hepatitis a with hepatic coma
**701**	0701 Viral hepatitis a without mention of hepatic coma
**702**	0702 Viral hepatitis b with hepatic coma
**703**	0703 Viral hepatitis b without mention of hepatic coma
**704**	0704 Other specified viral hepatitis with hepatic coma
**705**	0705 Other specified viral hepatitis without mention of hepatic coma
**706**	0706 Unspecified viral hepatitis with hepatic coma
**709**	0709 Unspecified viral hepatitis without mention of hepatic coma
**5716**	5716 Biliary cirrhosis
**5761**	5761 Cholangitis
**275**	275 Disorders of mineral metabolism
**2750**	2750 Disorders of iron metabolism
**27500**	27500 Haemosiderosis, primary
**27501**	27501 Haemosiderosis, acquired
**27502**	27502 Haemochromatosis
**27509**	27509 Disorders of iron metabolism (other and unspecified)
**2751**	2751 Disorders of copper metabolism

**2776**	2776 Other deficiencies of circulating enzymes
**27760**	27760 Other deficiencies of circulating enzymes (angio-oedema)
**27761**	27761 Other deficiencies of circulating enzymes (alpha-1-antitrypsin hepatitis)
**27762**	27762 Other alpha-1-antitrypsin deficiency
**27769**	27769 Other deficiencies of circulating enzymes (other and unspecified)
**4530**	4530 Budd-chiari syndrome
**5714**	5714 Chronic hepatitis
**303**	303 Alcohol dependence syndrome
**3039**	3039 Alcohol dependence syndrome
**3050**	3050 Nondependent abuse of alcohol
**291**	291 Alcoholic psychoses
**2910**	2910 Delirium tremens
**2911**	2911 Korsakov's psychosis, alcoholic
**2912**	2912 Other alcoholic dementia
**2913**	2913 Other alcoholic hallucinosis
**2914**	2914 Pathological drunkenness
**2915**	2915 Alcoholic jealousy
**2918**	2918 Other specified alcoholic psychoses
**2919**	2919 Alcoholic psychoses, unspecified
**3575**	3575 Alcoholic polyneuropathy
**4255**	4255 Alcoholic cardiomyopathy
**5353**	5353 Alcoholic gastritis
**9801**	9801 Toxic effect of methyl alcohol
**9809**	9809 Toxic effect of unspecified alcohol

Table S1B. ICD10 codes used to exclude participants from the study.

ICD10	UKB description
K70	K70 Alcoholic liver disease
K700	K70.0 Alcoholic fatty liver
K701	K70.1 Alcoholic hepatitis
K702	K70.2 Alcoholic fibrosis and sclerosis of liver
K703	K70.3 Alcoholic cirrhosis of liver
K704	K70.4 Alcoholic hepatic failure
K709	K70.9 Alcoholic liver disease, unspecified
B16	B16 Acute hepatitis B
B160	B16.0 Acute hepatitis B with delta-agent (coinfection) with hepatic coma
B161	B16.1 Acute hepatitis B with delta-agent (coinfection) without hepatic coma
B162	B16.2 Acute hepatitis B without delta-agent with hepatic coma
B169	B16.9 Acute hepatitis B without delta-agent and without hepatic coma
B17	B17 Other acute viral hepatitis
B170	B17.0 Acute delta-(super) infection of hepatitis B carrier
B171	B17.1 Acute hepatitis C
B172	B17.2 Acute hepatitis E
B178	B17.8 Other specified acute viral hepatitis
B179	B17.9 Acute viral hepatitis, unspecified
B18	B18 Chronic viral hepatitis
B180	B18.0 Chronic viral hepatitis B with delta-agent
B181	B18.1 Chronic viral hepatitis B without delta-agent
B182	B18.2 Chronic viral hepatitis C

B188	B18.8 Other chronic viral hepatitis
B189	B18.9 Chronic viral hepatitis, unspecified
B19	B19 Unspecified viral hepatitis
B190	B19.0 Unspecified viral hepatitis with coma
B199	B19.9 Unspecified viral hepatitis without coma
K743	K74.3 Primary biliary cirrhosis
K744	K74.4 Secondary biliary cirrhosis
K745	K74.5 Biliary cirrhosis, unspecified
K754	K75.4 Autoimmune hepatitis
K83	K83 Other diseases of biliary tract
K830	K83.0 Cholangitis
K831	K83.1 Obstruction of bile duct
K832	K83.2 Perforation of bile duct
K833	K83.3 Fistula of bile duct
K834	K83.4 Spasm of sphincter of Oddi
K835	K83.5 Biliary cyst
K838	K83.8 Other specified diseases of biliary tract
K839	K83.9 Disease of biliary tract, unspecified
E831	E83.1 Disorders of iron metabolism
P270	P27.0 Wilson-Mikity syndrome
E880	E88.0 Disorders of plasma-protein metabolism, not elsewhere classified
I820	I82.0 Budd-Chiari syndrome
K765	K76.5 Hepatic veno-occlusive disease
K73	K73 Chronic hepatitis, not elsewhere classified
K730	K73.0 Chronic persistent hepatitis, not elsewhere classified
K731	K73.1 Chronic lobular hepatitis, not elsewhere classified

K732	K73.2 Chronic active hepatitis, not elsewhere classified
K738	K73.8 Other chronic hepatitis, not elsewhere classified
K739	K73.9 Chronic hepatitis, unspecified
F10	F10 Mental and behavioural disorders due to use of alcohol
F100	F10.0 Acute intoxication
F101	F10.1 Harmful use
F102	F10.2 Dependence syndrome
F103	F10.3 Withdrawal state
F104	F10.4 Withdrawal state with delirium
F105	F10.5 Psychotic disorder
F106	F10.6 Amnesic syndrome
F107	F10.7 Residual and late-onset psychotic disorder
F108	F10.8 Other mental and behavioural disorders
F109	F10.9 Unspecified mental and behavioural disorder
E244	E24.4 Alcohol-induced pseudo-Cushing's syndrome
G621	G62.1 Alcoholic polyneuropathy
I426	I42.6 Alcoholic cardiomyopathy
K292	K29.2 Alcoholic gastritis
G312	G31.2 Degeneration of nervous system due to alcohol
G721	G72.1 Alcoholic myopathy
K852	K85.2 Alcohol-induced acute pancreatitis
K860	K86.0 Alcohol-induced chronic pancreatitis
T510	T51.0 Ethanol
T519	T51.9 Alcohol, unspecified
Y573	Y57.3 Alcohol deterrents
X65	X65 Intentional self-poisoning by and exposure to alcohol

X650	X65.0 Home
X6509	X65.09 Intentional self-poisoning by and exposure to alcohol, Home, During unspecified activity
X651	X65.1 Residential institution
X652	X65.2 School, other institution and public administrative area
X653	X65.3 Sports and athletics area
X654	X65.4 Street and highway
X655	X65.5 Trade and service area
X656	X65.6 Industrial and construction area
X657	X65.7 Farm
X658	X65.8 Other specified place
X659	X65.9 Unspecified place
X6598	X65.98 Intentional self-poisoning by and exposure to alcohol; Unspecified place; While engaged in other specified activities
X6599	X65.99 Intentional self-poisoning by and exposure to alcohol, Unspecified place, During unspecified activity
Z502	Z50.2 Alcohol rehabilitation
Z714	Z71.4 Alcohol abuse counselling and surveillance
Z721	Z72.1 Alcohol use

Table S2. List of associated variants in the case control analysis.

CHROM	POS	ID	REF	ALT	ALT_CASE_FREQ	ALT_CTRL_FREQ	OR	SE	Z_STAT	P	Func.refGene	Gene.refGene	GeneDetail.refGene	ExonicFunc.refGene	AAChange.refGene
22	44324730	rs738408	C	T	0.335	0.212	1.918	0.057	11.379	5.4E-30	exonic	PNPLA3	.	synonymous SNV	PNPLA3:NM_025225:exon3:c.C447T:p.P149P
22	44324727	rs738409	C	G	0.335	0.212	1.917	0.057	11.360	6.6E-30	exonic	PNPLA3	.	nonsynonymous SNV	PNPLA3:NM_025225:exon3:c.C444G:p.I148M
22	44324855	rs3747207	G	A	0.333	0.210	1.917	0.057	11.356	6.9E-30	intronic	PNPLA3	.	.	.
22	44340904	rs2294915	C	T	0.349	0.226	1.872	0.056	11.104	1.2E-28	intronic	PNPLA3	.	.	.
19	19379549	rs58542926	C	T	0.145	0.069	2.368	0.079	10.877	1.5E-27	exonic	TM6SF2	.	nonsynonymous SNV	TM6SF2:NM_001001524:exon6:c.G499A:p.E167K
19	19393890	rs200210321	A	AG	0.143	0.067	2.386	0.080	10.876	1.5E-27	intronic	SUGP1	.	.	.
19	19388500	rs8107974	A	T	0.147	0.070	2.352	0.079	10.828	2.5E-27	intronic	SUGP1	.	.	.
19	19370340	rs756350040	TGACA	T	0.140	0.066	2.393	0.081	10.791	3.8E-27	.	.	.	.	.
19	19407718	rs10401969	T	C	0.146	0.070	2.330	0.079	10.718	8.4E-27	intronic	SUGP1	.	.	.
19	19456917	rs58489806	C	T	0.157	0.080	2.197	0.076	10.342	4.5E-25	intronic	MAU2	.	.	.
22	44379565	rs2294922	G	C	0.312	0.204	1.831	0.059	10.337	4.8E-25	intronic	SAMM50	.	.	.
19	19432290	19:19432290_AG_A	AG	A	0.132	0.064	2.343	0.083	10.264	1.0E-24	.	.	.	.	.
19	19419071	rs739846	G	A	0.142	0.070	2.276	0.080	10.246	1.2E-24	intronic	SUGP1	.	.	.
19	19460541	rs73001065	G	C	0.132	0.065	2.299	0.082	10.103	5.3E-24	intronic	MAU2	.	.	.
19	19494483	rs150268548	G	A	0.128	0.062	2.303	0.084	9.975	2.0E-23	intergenic	MAU2;GATAD2A	dist=24920;dist=2169	.	.
19	19477877	rs56255430	A	C	0.148	0.077	2.150	0.078	9.767	1.6E-22	intergenic	MAU2;GATAD2A	dist=8314;dist=18775	.	.
19	19366632	rs72999033	C	T	0.120	0.058	2.298	0.086	9.645	5.1E-22	UTR3	HAPLN4	NM_023002:c.*1994G>A	.	.
19	19702384	rs17217098	G	A	0.126	0.063	2.222	0.084	9.557	1.2E-21	intronic	PBX4	.	.	.
19	19699398	19:19699398_GA_G	GA	G	0.125	0.062	2.214	0.084	9.442	3.7E-21	.	.	.	.	.
19	19329924	rs2228603	C	T	0.135	0.069	2.119	0.080	9.334	1.0E-20	exonic	NCAN	.	nonsynonymous SNV	NCAN:NM_004386:exon3:c.C274T:p.P92S
19	19717056	rs73004967	A	G	0.125	0.063	2.181	0.084	9.290	1.5E-20	intronic	PBX4	.	.	.
19	19610596	rs3794991	C	T	0.145	0.078	2.055	0.079	9.145	6.0E-20	intronic	GATAD2A	.	.	.
19	19578743	rs73002956	A	G	0.145	0.078	2.057	0.079	9.143	6.1E-20	intronic	GATAD2A	.	.	.
19	19670610	rs150824230	G	A	0.141	0.076	2.042	0.079	8.987	2.5E-19	intergenic	CILP2;PBX4	dist=13142;dist=1912	.	.
19	19671266	rs73004926	C	T	0.140	0.075	2.041	0.080	8.946	3.7E-19	intergenic	CILP2;PBX4	dist=13798;dist=1256	.	.
19	19675696	rs73004933	C	T	0.140	0.075	2.040	0.080	8.943	3.8E-19	intronic	PBX4	.	.	.
19	19662220	rs17216525	C	T	0.139	0.075	2.039	0.080	8.936	4.0E-19	intergenic	CILP2;PBX4	dist=4752;dist=10302	.	.
22	44332888	rs36038527	T	TC	0.248	0.162	1.742	0.062	8.920	4.7E-19	intronic	PNPLA3	.	.	.
19	19685470	rs141756246	G	GT	0.139	0.075	2.038	0.080	8.913	4.9E-19	intronic	PBX4	.	.	.
22	44325516	rs12485100	G	T	0.240	0.155	1.758	0.063	8.909	5.2E-19	intronic	PNPLA3	.	.	.
22	44325631	rs12484809	C	T	0.240	0.155	1.758	0.063	8.909	5.2E-19	intronic	PNPLA3	.	.	.
22	44325565	rs12484801	C	T	0.240	0.155	1.758	0.063	8.906	5.3E-19	intronic	PNPLA3	.	.	.
19	19667254	rs143988316	C	T	0.140	0.076	2.028	0.079	8.895	5.8E-19	intergenic	CILP2;PBX4	dist=9786;dist=5268	.	.
22	44326700	rs11090617	C	T	0.240	0.155	1.755	0.063	8.890	6.1E-19	intronic	PNPLA3	.	.	.
22	44326272	rs9625962	T	C	0.240	0.155	1.755	0.063	8.889	6.2E-19	intronic	PNPLA3	.	.	.
22	44325996	rs12483959	G	A	0.240	0.155	1.755	0.063	8.888	6.2E-19	intronic	PNPLA3	.	.	.
22	44341193	rs4823179	T	C	0.248	0.162	1.739	0.062	8.875	7.0E-19	intronic	PNPLA3	.	.	.
22	44336957	rs73176497	G	A	0.248	0.162	1.737	0.062	8.869	7.4E-19	intronic	PNPLA3	.	.	.
22	44332570	rs2281135	G	A	0.248	0.162	1.736	0.062	8.861	8.0E-19	intronic	PNPLA3	.	.	.
22	44331513	rs1997693	C	G	0.248	0.162	1.740	0.063	8.856	8.3E-19	intronic	PNPLA3	.	.	.
22	44339526	rs13056555	C	G	0.248	0.162	1.737	0.062	8.855	8.4E-19	intronic	PNPLA3	.	.	.
22	44340086	rs36069781	C	T	0.248	0.162	1.737	0.062	8.855	8.4E-19	intronic	PNPLA3	.	.	.
22	44341606	rs4823181	T	C	0.248	0.162	1.736	0.062	8.853	8.5E-19	intronic	PNPLA3	.	.	.
22	44333172	rs2072906	A	G	0.248	0.162	1.735	0.062	8.853	8.5E-19	intronic	PNPLA3	.	.	.
22	44334486	rs4823177	T	C	0.248	0.162	1.735	0.062	8.853	8.6E-19	intronic	PNPLA3	.	.	.
22	44334529	rs4823178	T	C	0.248	0.162	1.735	0.062	8.853	8.6E-19	intronic	PNPLA3	.	.	.
22	44334476	rs4823176	T	C	0.248	0.162	1.735	0.062	8.851	8.7E-19	intronic	PNPLA3	.	.	.
22	44340922	rs2294916	T	G	0.248	0.162	1.736	0.062	8.850	8.7E-19	intronic	PNPLA3	.	.	.
22	44341298	rs4823180	G	A	0.248	0.162	1.736	0.062	8.850	8.7E-19	intronic	PNPLA3	.	.	.
22	44333479	rs2072905	C	G	0.248	0.162	1.735	0.062	8.850	8.7E-19	intronic	PNPLA3	.	.	.
22	44333694	rs2896019	T	G	0.248	0.162	1.735	0.062	8.850	8.7E-19	intronic	PNPLA3	.	.	.
22	44333945	rs2401512	C	G	0.248	0.162	1.735	0.062	8.850	8.7E-19	intronic	PNPLA3	.	.	.
22	44333968	rs2896020	T	C	0.248	0.162	1.735	0.062	8.850	8.7E-19	intronic	PNPLA3	.	.	.
22	44332878	rs34879941	C	T	0.248	0.162	1.735	0.062	8.850	8.7E-19	intronic	PNPLA3	.	.	.
22	44335331	rs16991175	T	C	0.248	0.162	1.735	0.062	8.849	8.8E-19	intronic	PNPLA3	.	.	.
22	44335406	rs35621602	C	A	0.248	0.162	1.735	0.062	8.849	8.8E-19	intronic	PNPLA3	.	.	.
22	44335416	rs34352134	C	T	0.248	0.162	1.735	0.062	8.849	8.8E-19	intronic	PNPLA3	.	.	.
22	44335453	rs34376930	G	T	0.248	0.162	1.735	0.062	8.849	8.8E-19	intronic	PNPLA3	.	.	.
22	44335744	rs2073081	T	C	0.248	0.162	1.735	0.062	8.847	9.0E-19	intronic	PNPLA3	.	.	.
22	44336098	rs1010023	T	C	0.248	0.162	1.734	0.062	8.843	9.3E-19	intronic	PNPLA3	.	.	.

Table continues on next page.

CHROM	POS	ID	REF	ALT	ALT_CASE_FREQ	ALT_CTRL_FREQ	OR	SE	Z_STAT	P	Func.refGene	Gene.refGene	GeneDetail.refGene	ExonicFunc.refGene	AAChange.refGene
22	44336310	rs1010022	A	G	0.248	0.162	1.734	0.062	8.843	9.3E-19	intronic	PNPLA3	.	.	.
22	44336496	rs8142145	T	C	0.248	0.162	1.734	0.062	8.843	9.3E-19	intronic	PNPLA3	.	.	.
19	19664077	rs17216588	C	T	0.140	0.076	2.020	0.080	8.838	9.7E-19	intergenic	CILP2;PBX4	dist=6609;dist=8445	.	.
19	19658472	rs16996148	G	T	0.140	0.076	2.019	0.080	8.837	9.8E-19	intergenic	CILP2;PBX4	dist=1004;dist=14050	.	.
22	44331943	rs1883349	G	A	0.247	0.161	1.735	0.062	8.826	1.1E-18	intronic	PNPLA3	.	.	.
22	44329275	rs2294433	G	A	0.239	0.155	1.752	0.064	8.816	1.2E-18	intronic	PNPLA3	.	.	.
19	19746151	rs2304128	G	T	0.133	0.071	2.061	0.082	8.815	1.2E-18	intronic	GMIP	.	.	.
22	44337533	rs926633	G	A	0.248	0.162	1.731	0.062	8.808	1.3E-18	intronic	PNPLA3	.	.	.
22	44331778	rs13056638	C	G	0.248	0.162	1.734	0.063	8.804	1.3E-18	intronic	PNPLA3	.	.	.
19	19713069	rs73004962	A	T	0.141	0.077	2.005	0.079	8.802	1.3E-18	intronic	PBX4	.	.	.
22	44332653	rs2072907	C	G	0.249	0.163	1.730	0.062	8.800	1.4E-18	intronic	PNPLA3	.	.	.
22	44333370	rs2076207	A	G	0.249	0.163	1.730	0.062	8.799	1.4E-18	intronic	PNPLA3	.	.	.
22	44332477	rs2281138	T	C	0.249	0.163	1.730	0.062	8.799	1.4E-18	intronic	PNPLA3	.	.	.
22	44334842	rs2281293	T	C	0.249	0.163	1.730	0.062	8.798	1.4E-18	intronic	PNPLA3	.	.	.
22	44332493	rs2281137	T	C	0.249	0.163	1.730	0.062	8.798	1.4E-18	intronic	PNPLA3	.	.	.
19	19700552	rs12608729	C	T	0.142	0.078	2.001	0.079	8.788	1.5E-18	intronic	PBX4	.	.	.
22	44331815	rs1883348	C	G	0.248	0.163	1.732	0.063	8.788	1.5E-18	intronic	PNPLA3	.	.	.
22	44330031	rs1977080	C	T	0.241	0.157	1.746	0.063	8.787	1.5E-18	intronic	PNPLA3	.	.	.
19	19695228	rs73004951	C	T	0.141	0.077	2.003	0.079	8.766	1.8E-18	intronic	PBX4	.	.	.
22	44342969	rs2008451	T	C	0.247	0.162	1.728	0.062	8.761	1.9E-18	UTR3	PNPLA3	NM_025225:c.*707T>C	.	.
22	44343151	rs1810508	A	G	0.247	0.162	1.728	0.062	8.761	1.9E-18	UTR3	PNPLA3	NM_025225:c.*889A>G	.	.
22	44341666	rs13055900	A	G	0.247	0.162	1.728	0.062	8.760	2.0E-18	intronic	PNPLA3	.	.	.
22	44341672	rs13055874	T	C	0.247	0.162	1.728	0.062	8.760	2.0E-18	intronic	PNPLA3	.	.	.
22	44356468	rs56373884	G	A	0.234	0.151	1.747	0.064	8.757	2.0E-18	intronic	SAMM50	.	.	.
22	44328730	rs4823173	G	A	0.238	0.155	1.743	0.064	8.746	2.2E-18	intronic	PNPLA3	.	.	.
22	44329078	rs2076211	C	T	0.238	0.155	1.743	0.064	8.745	2.2E-18	intronic	PNPLA3	.	.	.
22	44345771	rs13054885	G	A	0.247	0.162	1.727	0.063	8.737	2.4E-18	intergenic	PNPLA3;SAMM50	dist=2309;dist=5551	.	.
22	44347251	rs2092501	G	A	0.232	0.150	1.750	0.064	8.736	2.4E-18	intergenic	PNPLA3;SAMM50	dist=3789;dist=4071	.	.
22	44327179	rs16991158	G	A	0.238	0.155	1.742	0.064	8.735	2.4E-18	intronic	PNPLA3	.	.	.
22	44327192	rs36055245	A	G	0.238	0.155	1.741	0.064	8.733	2.5E-18	intronic	PNPLA3	.	.	.
22	44330128	rs1977081	T	C	0.242	0.158	1.742	0.064	8.731	2.5E-18	intronic	PNPLA3	.	.	.
22	44327273	rs12484700	A	G	0.240	0.157	1.741	0.064	8.727	2.6E-18	intronic	PNPLA3	.	.	.
22	44361842	rs2294921	C	T	0.236	0.153	1.739	0.063	8.727	2.6E-18	intronic	SAMM50	.	.	.
22	44349236	rs1474745	T	C	0.234	0.151	1.745	0.064	8.724	2.7E-18	intergenic	PNPLA3;SAMM50	dist=5774;dist=2086	.	.
19	19721722	rs12610185	G	A	0.141	0.077	1.994	0.079	8.722	2.7E-18	intronic	PBX4	.	.	.
19	19721976	rs12610191	C	T	0.141	0.077	1.994	0.079	8.722	2.7E-18	intronic	PBX4	.	.	.
19	19726022	rs58847337	G	A	0.141	0.077	1.992	0.079	8.713	3.0E-18	intronic	PBX4	.	.	.
19	19720399	rs57504626	C	T	0.141	0.077	1.991	0.079	8.708	3.1E-18	intronic	PBX4	.	.	.
19	19720788	rs16996185	T	G	0.141	0.077	1.991	0.079	8.708	3.1E-18	intronic	PBX4	.	.	.
19	19723215	rs10500212	C	T	0.141	0.077	1.991	0.079	8.708	3.1E-18	intronic	PBX4	.	.	.
19	19716558	rs73004966	C	T	0.141	0.077	1.989	0.079	8.695	3.5E-18	intronic	PBX4	.	.	.
22	44348446	rs34912062	G	T	0.232	0.150	1.743	0.064	8.688	3.7E-18	intergenic	PNPLA3;SAMM50	dist=4984;dist=2876	.	.
22	44368122	rs3761472	A	G	0.236	0.154	1.734	0.063	8.681	3.9E-18	exonic	SAMM50	.	nonsynonymous SNV	SAMM50:NM_015380:exon5:c.A329G:p.D110G
19	19727152	rs73004975	A	G	0.141	0.078	1.986	0.079	8.671	4.3E-18	intronic	PBX4	.	.	.
19	19711139	rs73004959	C	T	0.140	0.077	1.989	0.079	8.666	4.5E-18	intronic	PBX4	.	.	.
22	44343626	rs12484795	A	C	0.253	0.169	1.685	0.062	8.377	5.4E-17	downstream	PNPLA3	dist=164	.	.
22	44381482	rs4823109	C	T	0.211	0.138	1.713	0.066	8.125	4.5E-16	intronic	SAMM50	.	.	.
19	19425025	rs57962361	C	T	0.175	0.107	1.793	0.072	8.125	4.5E-16	intronic	SUGP1	.	.	.
22	44381340	rs4823108	T	C	0.211	0.138	1.711	0.066	8.111	5.0E-16	intronic	SAMM50	.	.	.
22	44377999	rs2235776	C	T	0.211	0.138	1.709	0.066	8.106	5.2E-16	intronic	SAMM50	.	.	.
22	44378672	rs4823183	C	A	0.211	0.138	1.709	0.066	8.106	5.2E-16	intronic	SAMM50	.	.	.
22	44378809	rs2235777	C	T	0.211	0.138	1.709	0.066	8.106	5.2E-16	intronic	SAMM50	.	.	.
22	44380170	rs71313378	G	GCTTC	0.211	0.138	1.708	0.066	8.097	5.6E-16	intronic	SAMM50	.	.	.
22	44380767	rs12167845	T	C	0.211	0.138	1.707	0.066	8.081	6.4E-16	intronic	SAMM50	.	.	.
19	19436229	rs111234557	C	G	0.175	0.107	1.788	0.072	8.080	6.5E-16	intronic	MAU2	.	.	.
22	44371406	rs61473277	A	G	0.211	0.138	1.706	0.066	8.077	6.6E-16	intronic	SAMM50	.	.	.
22	44379740	rs2294923	C	A	0.211	0.138	1.706	0.066	8.072	6.9E-16	intronic	SAMM50	.	.	.
22	44380009	rs9626079	A	G	0.211	0.138	1.706	0.066	8.072	6.9E-16	intronic	SAMM50	.	.	.
19	19426181	rs11668104	G	A	0.175	0.107	1.782	0.072	8.038	9.1E-16	intronic	SUGP1	.	.	.
19	19462702	rs11672355	G	C	0.175	0.107	1.778	0.072	8.011	1.1E-15	intronic	MAU2	.	.	.

Table continues on next page.

CHROM	POS	ID	REF	ALT	ALT_CASE_FREQ	ALT_CTRL_FREQ	OR	SE	Z_STAT	P	Func.refGene	Gene.refGene	GeneDetail.refGene	ExonicFunc.refGene	AAChange.refGene
19	19440064	rs11411903	T	TA	0.175	0.107	1.777	0.072	7.990	1.3E-15	intronic	MAU2	.	.	.
19	19450254	19:19450254_CA_C	CA	C	0.182	0.114	1.738	0.071	7.731	1.1E-14	.	.	.	.	.
19	19325963	rs3761077	G	T	0.162	0.103	1.723	0.073	7.445	9.7E-14	intronic	NCAN	.	.	.
19	19756073	19:19756073_AGCC_A	AGCC	A	0.112	0.063	1.912	0.087	7.426	1.1E-13	.	.	.	.	.
19	19467545	rs2285626	C	T	0.184	0.119	1.681	0.070	7.417	1.2E-13	UTR3	MAU2	NM_015329:c.*954C>T	.	.
19	19406869	rs12979148	T	C	0.189	0.123	1.662	0.069	7.385	1.5E-13	intronic	SUGP1	.	.	.
19	19793545	rs56408111	T	C	0.132	0.077	1.819	0.081	7.378	1.6E-13	UTR3	ZNF101	NM_001300949:c.*2436T>C;NM_033204:c.*2436T>C	.	.
19	19786099	rs58434384	A	G	0.135	0.081	1.783	0.079	7.290	3.1E-13	intronic	ZNF101	.	.	.
19	19336608	rs2238675	C	T	0.184	0.121	1.666	0.070	7.281	3.3E-13	intronic	NCAN	.	.	.
19	19789528	rs2304130	A	G	0.135	0.081	1.779	0.079	7.273	3.5E-13	intronic	ZNF101	.	.	.
19	19485105	rs12052117	C	T	0.179	0.116	1.667	0.071	7.182	6.9E-13	intergenic	MAU2;GATAD2A	dist=15542;dist=11547	.	.
19	19418916	rs2240117	C	T	0.186	0.123	1.640	0.069	7.152	8.5E-13	intronic	SUGP1	.	.	.
22	44361713	rs12165526	T	A	0.147	0.093	1.736	0.077	7.116	1.1E-12	intronic	SAMM50	.	.	.
19	19582992	rs73002960	C	T	0.177	0.116	1.656	0.071	7.081	1.4E-12	intronic	GATAD2A	.	.	.
19	19572220	rs28720066	G	T	0.177	0.116	1.654	0.071	7.063	1.6E-12	intronic	GATAD2A	.	.	.
19	19484008	rs59148799	A	G	0.178	0.116	1.651	0.071	7.037	2.0E-12	intergenic	MAU2;GATAD2A	dist=14445;dist=12644	.	.
19	19506092	rs56241616	C	T	0.177	0.116	1.649	0.071	7.028	2.1E-12	intronic	GATAD2A	.	.	.
19	19503573	rs10408875	T	C	0.177	0.116	1.649	0.071	7.027	2.1E-12	intronic	GATAD2A	.	.	.
19	19512657	rs10408596	A	T	0.177	0.116	1.649	0.071	7.024	2.2E-12	intronic	GATAD2A	.	.	.
19	19539891	rs8182472	T	C	0.177	0.116	1.648	0.071	7.018	2.2E-12	intronic	GATAD2A	.	.	.
19	19505087	rs10415849	C	T	0.177	0.116	1.647	0.071	7.017	2.3E-12	intronic	GATAD2A	.	.	.
19	19621004	rs56273306	T	C	0.177	0.116	1.647	0.071	7.012	2.3E-12	intergenic	GATAD2A;TSSK6	dist=1263;dist=4024	.	.
19	19513568	rs34324111	T	G	0.177	0.116	1.642	0.071	6.973	3.1E-12	intronic	GATAD2A	.	.	.
19	19513572	rs35629458	T	G	0.177	0.116	1.642	0.071	6.973	3.1E-12	intronic	GATAD2A	.	.	.
19	19513580	rs113460678	T	G	0.177	0.116	1.642	0.071	6.973	3.1E-12	intronic	GATAD2A	.	.	.
19	19642795	rs56397647	C	T	0.170	0.112	1.619	0.072	6.672	2.5E-11	intronic	YJEFN3	.	.	.
19	19393677	rs35431065	A	G	0.211	0.148	1.547	0.066	6.623	3.5E-11	intronic	SUGP1	.	.	.
19	19531910	rs11668386	A	G	0.180	0.121	1.589	0.070	6.590	4.4E-11	intronic	GATAD2A	.	.	.
19	19621197	rs113365218	G	A	0.170	0.113	1.607	0.072	6.553	5.6E-11	intergenic	GATAD2A;TSSK6	dist=1456;dist=3831	.	.
19	19508013	rs10424702	A	G	0.180	0.121	1.582	0.070	6.531	6.5E-11	intronic	GATAD2A	.	.	.
19	19517169	rs188552254	A	G	0.180	0.121	1.578	0.070	6.495	8.3E-11	intronic	GATAD2A	.	.	.
19	19548643	rs79954596	T	G	0.180	0.121	1.577	0.070	6.483	9.0E-11	intronic	GATAD2A	.	.	.
19	19613622	rs57009615	A	G	0.185	0.126	1.567	0.069	6.479	9.2E-11	intronic	GATAD2A	.	.	.
19	19665581	rs34755166	G	A	0.178	0.120	1.575	0.070	6.472	9.7E-11	intergenic	CILP2;PBX4	dist=8113;dist=6941	.	.
19	19666574	rs17216693	T	C	0.180	0.121	1.572	0.070	6.470	9.8E-11	intergenic	CILP2;PBX4	dist=9106;dist=5948	.	.
22	44328075	rs9306471	A	G	0.095	0.056	1.795	0.093	6.308	2.8E-10	intronic	PNPLA3	.	.	.
22	44377442	rs4823182	A	G	0.404	0.327	1.406	0.054	6.298	3.0E-10	intronic	SAMM50	.	.	.
19	19668338	19:19668338_GC_G	GC	G	0.177	0.121	1.556	0.070	6.285	3.3E-10	.	.	.	.	.
22	44376335	rs67450864	C	T	0.404	0.327	1.405	0.054	6.280	3.4E-10	intronic	SAMM50	.	.	.
22	44327075	rs9626056	C	T	0.095	0.056	1.790	0.093	6.277	3.4E-10	intronic	PNPLA3	.	.	.
22	44329719	rs111393709	C	T	0.095	0.056	1.791	0.093	6.270	3.6E-10	intronic	PNPLA3	.	.	.
19	19207229	rs80341032	G	A	0.089	0.053	1.805	0.097	6.117	9.6E-10	intronic	SLC25A42	.	.	.
22	44391234	rs2281298	G	A	0.241	0.179	1.463	0.062	6.114	9.7E-10	intronic	SAMM50	.	.	.
22	44387932	rs10656207	C	CTA	0.462	0.383	1.381	0.053	6.098	1.1E-09	intronic	SAMM50	.	.	.
22	44388817	rs3827385	T	C	0.243	0.181	1.456	0.062	6.073	1.3E-09	intronic	SAMM50	.	.	.
22	44391686	rs2143571	G	A	0.240	0.179	1.460	0.062	6.071	1.3E-09	intronic	SAMM50	.	.	.
22	44394019	rs2401514	T	A	0.242	0.180	1.457	0.062	6.067	1.3E-09	intergenic	SAMM50;PARVB	dist=1610;dist=1072	.	.
22	44382684	rs2294927	T	C	0.460	0.382	1.378	0.053	6.067	1.3E-09	intronic	SAMM50	.	.	.
22	44385594	rs2073079	A	G	0.242	0.180	1.457	0.062	6.065	1.3E-09	intronic	SAMM50	.	.	.
22	44394402	rs2073080	C	T	0.242	0.180	1.455	0.062	6.042	1.5E-09	upstream	PARVB	dist=689	.	.
22	44383400	rs6006602	C	T	0.459	0.382	1.375	0.053	6.017	1.8E-09	intronic	SAMM50	.	.	.
22	44383432	rs6006468	G	C	0.459	0.382	1.375	0.053	6.017	1.8E-09	intronic	SAMM50	.	.	.
19	19155672	rs73008942	G	A	0.083	0.049	1.820	0.100	6.004	1.9E-09	intronic	ARMC6	.	.	.
22	44383617	rs6006469	C	G	0.459	0.382	1.373	0.053	5.991	2.1E-09	intronic	SAMM50	.	.	.
22	44324558	rs55768287	C	T	0.090	0.054	1.765	0.095	5.983	2.2E-09	intronic	PNPLA3	.	.	.
22	44389514	rs2235778	T	C	0.459	0.382	1.372	0.053	5.980	2.2E-09	intronic	SAMM50	.	.	.
22	44335670	22:44335670_TGG_T	TGG	T	0.541	0.467	1.372	0.053	5.975	2.3E-09	.	.	.	.	.
22	44388417	rs3788604	A	G	0.459	0.382	1.370	0.053	5.960	2.5E-09	intronic	SAMM50	.	.	.
22	44395451	rs1007863	T	C	0.460	0.383	1.370	0.053	5.959	2.5E-09	exonic	PARVB	.	nonsynonymous SNV	PARVB:NM_001003828:exon2:c.T109C:p.W37R
22	44387108	rs1986095	A	G	0.459	0.383	1.369	0.053	5.952	2.6E-09	intronic	SAMM50	.	.	.

Table continues on next page.

CHROM	POS	ID	REF	ALT	ALT_CASE_FREQ	ALT_CTRL_FREQ	OR	SE	Z_STAT	P	Func.refGene	Gene.refGene	GeneDetail.refGene	ExonicFunc.refGene	AAChange.refGene
19	19374061	rs8105094	C	T	0.223	0.162	1.467	0.064	5.948	2.7E-09	upstream	HAPLN4	dist=448	.	.
19	19374068	rs8105984	T	C	0.223	0.162	1.467	0.064	5.948	2.7E-09	upstream	HAPLN4	dist=455	.	.
22	44393075	rs6006473	C	T	0.459	0.382	1.369	0.053	5.936	2.9E-09	downstream	SAMM50	dist=666	.	.
22	44395389	rs2281292	A	C	0.459	0.383	1.365	0.053	5.889	3.9E-09	intronic	PARVB	.	.	.
22	44337610	rs9625964	G	A	0.101	0.064	1.691	0.090	5.870	4.4E-09	intronic	PNPLA3	.	.	.
22	44338105	rs9625966	C	T	0.101	0.064	1.691	0.090	5.869	4.4E-09	intronic	PNPLA3	.	.	.
22	44338049	rs9625965	T	C	0.101	0.064	1.691	0.090	5.865	4.5E-09	intronic	PNPLA3	.	.	.
19	19110422	rs73006914	C	T	0.081	0.049	1.801	0.101	5.848	5.0E-09	intronic	SUGP2	.	.	.
22	44339791	rs9626057	C	G	0.101	0.064	1.687	0.090	5.841	5.2E-09	intronic	PNPLA3	.	.	.
22	44339055	rs117772800	A	G	0.101	0.064	1.685	0.090	5.826	5.7E-09	intronic	PNPLA3	.	.	.
22	44383070	rs12166587	T	C	0.104	0.067	1.672	0.088	5.817	6.0E-09	intronic	SAMM50	.	.	.
22	44383502	rs9625970	T	C	0.104	0.067	1.671	0.088	5.816	6.0E-09	intronic	SAMM50	.	.	.
22	44385583	rs112902984	T	C	0.104	0.067	1.670	0.088	5.810	6.3E-09	intronic	SAMM50	.	.	.
22	44387298	rs117472787	C	T	0.104	0.067	1.670	0.088	5.810	6.3E-09	intronic	SAMM50	.	.	.
19	19103986	rs3810444	T	A	0.089	0.055	1.748	0.096	5.803	6.5E-09	UTR3	SUGP2	NM_001321699:c.*564A>T;NM_001352071:c.*564A>T;NM_001017392:c.*564A>T	.	.
22	44342691	rs41278873	T	C	0.104	0.066	1.666	0.088	5.783	7.3E-09	UTR3	PNPLA3	NM_025225:c.*429T>C	.	.
22	44393476	rs8141950	C	T	0.103	0.067	1.662	0.089	5.725	1.0E-08	intergenic	SAMM50;PARVB	dist=1067;dist=1615	.	.
19	19434042	rs58833986	T	TCACCA	0.226	0.167	1.437	0.064	5.697	1.2E-08	intronic	MAU2	.	.	.
22	44397144	rs8141994	A	G	0.102	0.066	1.655	0.089	5.643	1.7E-08	intronic	PARVB	.	.	.
19	19381715	rs2074301	G	A	0.226	0.168	1.433	0.064	5.643	1.7E-08	intronic	TM6SF2	.	.	.
19	19380996	rs2074300	G	T	0.226	0.168	1.432	0.064	5.631	1.8E-08	exonic	TM6SF2	.	synonymous SNV	TM6SF2:NM_001001524:exon4:c.C387A:p.G129G
22	44370439	rs738494	A	C	0.101	0.065	1.654	0.089	5.625	1.9E-08	intronic	SAMM50	.	.	.
22	44359938	rs9626071	C	A	0.101	0.065	1.652	0.089	5.612	2.0E-08	intronic	SAMM50	.	.	.
22	44356562	rs28421169	T	C	0.101	0.065	1.650	0.089	5.600	2.1E-08	intronic	SAMM50	.	.	.
22	44358819	rs28754570	G	C	0.101	0.065	1.650	0.089	5.597	2.2E-08	intronic	SAMM50	.	.	.
22	44359729	rs12170274	C	T	0.101	0.065	1.650	0.089	5.595	2.2E-08	intronic	SAMM50	.	.	.
22	44359651	rs12168138	T	C	0.101	0.065	1.649	0.089	5.594	2.2E-08	intronic	SAMM50	.	.	.
19	19467996	rs2285628	T	A	0.227	0.169	1.427	0.064	5.577	2.4E-08	UTR3	MAU2	NM_015329:c.*1405T>A	.	.
19	19374546	rs563530889	A	AAG	0.226	0.168	1.427	0.064	5.566	2.6E-08	upstream;downstream	HAPLN4;TM6SF2	dist=933;dist=630	.	.
19	19471241	rs10419672	T	C	0.228	0.169	1.423	0.064	5.540	3.0E-08	intergenic	MAU2;GATAD2A	dist=1678;dist=25411	.	.
22	44343352	rs9626058	A	G	0.100	0.064	1.645	0.090	5.523	3.3E-08	UTR3	PNPLA3	NM_025225:c.*1090A>G	.	.
19	19425141	rs12459676	A	T	0.226	0.169	1.421	0.064	5.515	3.5E-08	intronic	SUGP1	.	.	.
19	19393106	19:19393106_CAAGA_C	CAAGA	C	0.223	0.166	1.424	0.064	5.512	3.5E-08	.	.	.	.	.
19	19405480	rs2023883	G	A	0.225	0.168	1.421	0.064	5.506	3.7E-08	intronic	SUGP1	.	.	.
22	44341986	rs2294917	T	C	0.271	0.339	0.724	0.059	-5.503	3.7E-08	intronic	PNPLA3	.	.	.
19	19407171	rs11666553	C	A	0.225	0.168	1.420	0.064	5.495	3.9E-08	intronic	SUGP1	.	.	.
22	44368584	rs9626076	G	A	0.100	0.065	1.639	0.090	5.494	3.9E-08	intronic	SAMM50	.	.	.
22	44366874	rs9626075	G	A	0.100	0.065	1.639	0.090	5.493	3.9E-08	intronic	SAMM50	.	.	.
22	44344872	rs9626061	C	T	0.099	0.064	1.645	0.091	5.491	4.0E-08	intergenic	PNPLA3;SAMM50	dist=1410;dist=6450	.	.
22	44363736	rs9626074	C	T	0.100	0.065	1.638	0.090	5.487	4.1E-08	intronic	SAMM50	.	.	.
22	44369927	rs12330016	C	T	0.100	0.065	1.638	0.090	5.485	4.1E-08	intronic	SAMM50	.	.	.
22	44371030	rs117130990	G	A	0.100	0.065	1.638	0.090	5.485	4.1E-08	intronic	SAMM50	.	.	.
22	44362178	rs9626073	G	A	0.100	0.065	1.638	0.090	5.485	4.1E-08	intronic	SAMM50	.	.	.
22	44370955	rs73434655	A	G	0.100	0.065	1.637	0.090	5.482	4.2E-08	intronic	SAMM50	.	.	.
22	44366135	rs12168183	G	A	0.100	0.065	1.637	0.090	5.482	4.2E-08	intronic	SAMM50	.	.	.
19	19422152	19:19422152_CA_C	CA	C	0.224	0.167	1.420	0.064	5.479	4.3E-08	.	.	.	.	.
22	44358360	rs9626068	C	G	0.100	0.065	1.636	0.090	5.471	4.5E-08	intronic	SAMM50	.	.	.
19	19469296	rs10403731	G	A	0.226	0.169	1.417	0.064	5.470	4.5E-08	UTR3	MAU2	NM_015329:c.*2705G>A	.	.
19	19429220	rs12976025	C	T	0.226	0.169	1.416	0.064	5.468	4.5E-08	intronic	SUGP1	.	.	.
19	19435680	rs4808194	T	G	0.226	0.169	1.416	0.064	5.468	4.5E-08	intronic	MAU2	.	.	.
19	19419810	rs12983137	G	A	0.226	0.169	1.416	0.064	5.461	4.7E-08	intronic	SUGP1	.	.	.
19	19422187	rs1859287	T	C	0.226	0.169	1.416	0.064	5.461	4.7E-08	intronic	SUGP1	.	.	.
19	19428805	rs7259434	A	T	0.226	0.169	1.416	0.064	5.461	4.7E-08	intronic	SUGP1	.	.	.
19	19432959	rs10402661	A	G	0.226	0.169	1.416	0.064	5.461	4.7E-08	intronic	MAU2	.	.	.
22	44358030	rs9626067	C	T	0.100	0.065	1.634	0.090	5.460	4.8E-08	intronic	SAMM50	.	.	.
22	44357940	rs9626066	T	A	0.100	0.065	1.634	0.090	5.457	4.8E-08	intronic	SAMM50	.	.	.
22	44357928	rs9626065	A	C	0.100	0.065	1.633	0.090	5.455	4.9E-08	intronic	SAMM50	.	.	.
19	19448301	rs757000	A	G	0.226	0.169	1.415	0.064	5.452	5.0E-08	intronic	MAU2	.	.	.
19	19448808	rs757001	G	A	0.226	0.169	1.415	0.064	5.452	5.0E-08	intronic	MAU2	.	.	.
19	19452249	rs2301668	G	A	0.226	0.169	1.415	0.064	5.452	5.0E-08	intronic	MAU2	.	.	.
22	44373947	rs9626078	G	A	0.100	0.065	1.633	0.090	5.451	5.0E-08	intronic	SAMM50	.	.	.

Table continues on next page.

Table S3. Sex interaction analysis results in the Genome-wide significant SNPs from the CC GWAS. P-values were adjusted accounting for the number of independent SNPs (n = 19) according to the LD pattern.

ID	OR	P	ADJ-P
19:19393106_CAAGA_C	0.920	0.178	1.000
19:19422152_CA_C	0.927	0.221	1.000
19:19432290_AG_A	0.959	0.599	1.000
19:19450254_CA_C	0.952	0.474	1.000
19:19668338_GC_G	0.922	0.233	1.000
19:19699398_GA_G	0.941	0.459	1.000
19:19756073_AGCC_A	0.958	0.609	1.000
22:44335670_TGG_T	0.951	0.325	1.000
rs1007863	0.915	0.080	1.000
rs1010022	0.952	0.416	1.000
rs1010023	0.952	0.416	1.000
rs10401969	0.973	0.722	1.000
rs10402661	0.928	0.222	1.000
rs10403731	0.927	0.218	1.000
rs10408596	0.911	0.175	1.000
rs10408875	0.911	0.174	1.000
rs10415849	0.912	0.178	1.000
rs10419672	0.924	0.199	1.000
rs10424702	0.908	0.156	1.000
rs10500212	0.969	0.682	1.000
rs10656207	0.914	0.079	1.000
rs11090617	0.947	0.371	1.000
rs111234557	0.929	0.291	1.000
rs111393709	0.951	0.575	1.000
rs112902984	0.908	0.255	1.000
rs113365218	0.892	0.102	1.000
rs113460678	0.912	0.181	1.000
rs11411903	0.936	0.342	1.000
rs11666553	0.924	0.200	1.000

Table continues on next page.

ID	OR	P	ADJ-P
rs11668104	0.932	0.307	1.000
rs11668386	0.908	0.155	1.000
rs11672355	0.932	0.310	1.000
rs117130990	0.918	0.323	1.000
rs117472787	0.908	0.255	1.000
rs117772800	0.931	0.410	1.000
rs12052117	0.916	0.199	1.000
rs12165526	0.909	0.203	1.000
rs12166587	0.907	0.251	1.000
rs12167845	0.908	0.128	1.000
rs12168138	0.923	0.352	1.000
rs12168183	0.917	0.316	1.000
rs12170274	0.923	0.352	1.000
rs12330016	0.918	0.323	1.000
rs12459676	0.926	0.209	1.000
rs12483959	0.946	0.364	1.000
rs12484700	0.957	0.471	1.000
rs12484795	0.958	0.472	1.000
rs12484801	0.947	0.376	1.000
rs12484809	0.948	0.378	1.000
rs12485100	0.948	0.378	1.000
rs12608729	0.958	0.570	1.000
rs12610185	0.968	0.674	1.000
rs12610191	0.968	0.674	1.000
rs12976025	0.928	0.219	1.000
rs12979148	0.957	0.503	1.000
rs12983137	0.928	0.222	1.000
rs13054885	0.951	0.405	1.000
rs13055874	0.958	0.472	1.000
rs13055900	0.958	0.472	1.000

Table continues on next page.

ID	OR	P	ADJ-P
rs13056555	0.952	0.415	1.000
rs13056638	0.962	0.515	1.000
rs141756246	0.951	0.516	1.000
rs143988316	0.941	0.429	1.000
rs1474745	0.925	0.204	1.000
rs150268548	0.948	0.509	1.000
rs150824230	0.949	0.498	1.000
rs16991158	0.948	0.381	1.000
rs16991175	0.952	0.416	1.000
rs16996148	0.934	0.377	1.000
rs16996185	0.969	0.682	1.000
rs17216525	0.929	0.340	1.000
rs17216588	0.933	0.369	1.000
rs17216693	0.935	0.317	1.000
rs17217098	0.972	0.724	1.000
rs1810508	0.949	0.385	1.000
rs1859287	0.928	0.222	1.000
rs1883348	0.962	0.524	1.000
rs1883349	0.955	0.442	1.000
rs188552254	0.910	0.164	1.000
rs1977080	0.960	0.506	1.000
rs1977081	0.962	0.524	1.000
rs1986095	0.919	0.097	1.000
rs1997693	0.965	0.555	1.000
rs200210321	0.953	0.535	1.000
rs2008451	0.949	0.385	1.000
rs2023883	0.924	0.197	1.000
rs2072905	0.952	0.415	1.000
rs2072906	0.952	0.415	1.000
rs2072907	0.961	0.508	1.000

Table continues on next page.

ID	OR	P	ADJ-P
rs2073079	0.919	0.154	1.000
rs2073080	0.917	0.147	1.000
rs2073081	0.953	0.417	1.000
rs2074300	0.918	0.162	1.000
rs2074301	0.918	0.163	1.000
rs2076207	0.960	0.501	1.000
rs2076211	0.947	0.375	1.000
rs2092501	0.917	0.161	1.000
rs2143571	0.915	0.137	1.000
rs2228603	0.927	0.329	1.000
rs2235776	0.907	0.125	1.000
rs2235777	0.907	0.124	1.000
rs2235778	0.918	0.093	1.000
rs2238675	0.922	0.227	1.000
rs2240117	0.949	0.437	1.000
rs2281135	0.952	0.416	1.000
rs2281137	0.961	0.507	1.000
rs2281138	0.961	0.506	1.000
rs2281292	0.912	0.072	1.000
rs2281293	0.961	0.502	1.000
rs2281298	0.912	0.124	1.000
rs2285626	0.932	0.296	1.000
rs2285628	0.932	0.252	1.000
rs2294433	0.945	0.354	1.000
rs2294915	0.939	0.249	1.000
rs2294916	0.952	0.415	1.000
rs2294917	1.015	0.795	1.000
rs2294921	0.925	0.198	1.000
rs2294922	0.900	0.062	1.000
rs2294923	0.906	0.121	1.000

Table continues on next page.

ID	OR	P	ADJ-P
rs2294927	0.916	0.085	1.000
rs2301668	0.928	0.224	1.000
rs2304128	0.986	0.859	1.000
rs2304130	0.980	0.795	1.000
rs2401512	0.952	0.415	1.000
rs2401514	0.919	0.157	1.000
rs28421169	0.924	0.358	1.000
rs28720066	0.914	0.191	1.000
rs28754570	0.923	0.352	1.000
rs2896019	0.952	0.415	1.000
rs2896020	0.952	0.415	1.000
rs34324111	0.912	0.181	1.000
rs34352134	0.952	0.416	1.000
rs34376930	0.952	0.416	1.000
rs34755166	0.934	0.313	1.000
rs34879941	0.952	0.414	1.000
rs34912062	0.919	0.168	1.000
rs35431065	0.938	0.316	1.000
rs35621602	0.952	0.416	1.000
rs35629458	0.912	0.181	1.000
rs36038527	0.949	0.381	1.000
rs36055245	0.948	0.379	1.000
rs36069781	0.952	0.415	1.000
rs3747207	0.939	0.257	1.000
rs3761077	0.904	0.153	1.000
rs3761472	0.925	0.200	1.000
rs3788604	0.919	0.096	1.000
rs3794991	0.955	0.546	1.000
rs3810444	1.029	0.756	1.000
rs3827385	0.927	0.202	1.000

Table continues on next page.

ID	OR	P	ADJ-P
rs41278873	0.940	0.466	1.000
rs4808194	0.928	0.219	1.000
rs4823108	0.908	0.128	1.000
rs4823109	0.908	0.130	1.000
rs4823173	0.947	0.374	1.000
rs4823176	0.952	0.415	1.000
rs4823177	0.952	0.415	1.000
rs4823178	0.952	0.415	1.000
rs4823179	0.951	0.399	1.000
rs4823180	0.952	0.415	1.000
rs4823181	0.952	0.415	1.000
rs4823182	0.922	0.119	1.000
rs4823183	0.907	0.124	1.000
rs55768287	0.907	0.288	1.000
rs56241616	0.911	0.174	1.000
rs56255430	0.965	0.634	1.000
rs56273306	0.911	0.175	1.000
rs563530889	0.917	0.160	1.000
rs56373884	0.915	0.149	1.000
rs56397647	0.888	0.088	1.000
rs56408111	0.984	0.841	1.000
rs57009615	0.912	0.168	1.000
rs57504626	0.969	0.682	1.000
rs57962361	0.932	0.306	1.000
rs58434384	0.980	0.796	1.000
rs58489806	0.979	0.776	1.000
rs58542926	0.963	0.617	1.000
rs58833986	0.925	0.204	1.000
rs58847337	0.969	0.678	1.000
rs59148799	0.911	0.177	1.000

Table continues on next page.

ID	OR	P	ADJ-P
rs6006468	0.919	0.096	1.000
rs6006469	0.919	0.098	1.000
rs6006473	0.919	0.096	1.000
rs6006602	0.919	0.096	1.000
rs61473277	0.904	0.113	1.000
rs67450864	0.925	0.134	1.000
rs71313378	0.904	0.114	1.000
rs7259434	0.928	0.222	1.000
rs72999033	0.929	0.378	1.000
rs73001065	0.963	0.633	1.000
rs73002956	0.963	0.617	1.000
rs73002960	0.912	0.179	1.000
rs73004926	0.955	0.549	1.000
rs73004933	0.955	0.550	1.000
rs73004951	0.962	0.612	1.000
rs73004959	0.965	0.647	1.000
rs73004962	0.970	0.688	1.000
rs73004966	0.970	0.689	1.000
rs73004967	0.976	0.764	1.000
rs73004975	0.967	0.658	1.000
rs73006914	1.024	0.809	1.000
rs73008942	1.016	0.869	1.000
rs73176497	0.953	0.426	1.000
rs73434655	0.918	0.325	1.000
rs738408	0.940	0.262	1.000
rs738409	0.940	0.258	1.000
rs738494	0.926	0.371	1.000
rs739846	0.983	0.829	1.000
rs756350040	0.986	0.856	1.000
rs757000	0.928	0.224	1.000

Table continues on next page.

ID	OR	P	ADJ-P
rs757001	0.928	0.224	1.000
rs79954596	0.913	0.178	1.000
rs80341032	1.004	0.967	1.000
rs8105094	0.914	0.146	1.000
rs8105984	0.914	0.146	1.000
rs8107974	0.956	0.557	1.000
rs8141950	0.903	0.231	1.000
rs8141994	0.913	0.287	1.000
rs8142145	0.952	0.416	1.000
rs8182472	0.914	0.188	1.000
rs926633	0.954	0.432	1.000
rs9306471	0.951	0.570	1.000
rs9625962	0.947	0.372	1.000
rs9625964	0.932	0.411	1.000
rs9625965	0.932	0.414	1.000
rs9625966	0.932	0.412	1.000
rs9625970	0.907	0.253	1.000
rs9626056	0.951	0.572	1.000
rs9626057	0.932	0.412	1.000
rs9626058	0.929	0.397	1.000
rs9626061	0.922	0.352	1.000
rs9626065	0.915	0.307	1.000
rs9626066	0.915	0.306	1.000
rs9626067	0.915	0.306	1.000
rs9626068	0.915	0.302	1.000
rs9626071	0.923	0.350	1.000
rs9626073	0.916	0.309	1.000
rs9626074	0.916	0.310	1.000
rs9626075	0.916	0.312	1.000
rs9626076	0.916	0.311	1.000
rs9626078	0.917	0.315	1.000
rs9626079	0.906	0.121	1.000

Table S4. List of associated variants in the quantitative analysis.

CHROM	POS	ID	REF	ALT	BETA	SE	T_STAT	P	Func.refGene	Gene.refGene	GeneDetail.refGene	ExonicFunc.refGene	AAChange.refGene
22	44324730	rs738408	C	T	0.532	0.032	16.452	4.3E-60	exonic	*PNPLA3*	.	synonymous SNV	PNPLA3:NM_025225:exon3:c.C447T:p.P149P
22	44324727	rs738409	C	G	0.531	0.032	16.439	5.3E-60	exonic	*PNPLA3*	.	nonsynonymous SNV	PNPLA3:NM_025225:exon3:c.C444G:p.I148M
22	44324855	rs3747207	G	A	0.533	0.032	16.418	7.4E-60	intronic	*PNPLA3*	.	.	.
22	44340904	rs2294915	C	T	0.510	0.032	16.132	7.2E-58	intronic	*PNPLA3*	.	.	.
19	19393890	rs200210321	A	AG	0.766	0.052	14.751	8.9E-49	intronic	*SUGP1*	.	.	.
19	19379549	rs58542926	C	T	0.752	0.051	14.689	2.2E-48	exonic	*TM6SF2*	.	nonsynonymous SNV	TM6SF2:NM_001001524:exon6:c.G499A:p.E167K
19	19407718	rs10401969	T	C	0.748	0.051	14.668	3.0E-48	intronic	*SUGP1*	.	.	.
19	19388500	rs8107974	A	T	0.743	0.051	14.556	1.5E-47	intronic	*SUGP1*	.	.	.
19	19370340	rs756350040	TGACA	T	0.761	0.052	14.522	2.5E-47	.	.	.	.	.
22	44379565	rs2294922	G	C	0.478	0.033	14.443	7.6E-47	intronic	*SAMM50*	.	.	.
19	19432290	19:19432290_AG_A	AG	A	0.769	0.053	14.436	8.3E-47	.	.	.	.	.
19	19460541	rs73001065	G	C	0.755	0.053	14.309	5.0E-46	intronic	*MAU2*	.	.	.
19	19494483	rs150268548	G	A	0.768	0.054	14.298	5.9E-46	intergenic	*MAU2;GATAD2A*	dist=24920;dist=2169	.	.
19	19419071	rs739846	G	A	0.707	0.051	13.996	4.0E-44	intronic	*SUGP1*	.	.	.
19	19456917	rs58489806	C	T	0.667	0.048	13.823	4.3E-43	intronic	*MAU2*	.	.	.
22	44325516	rs12485100	G	T	0.507	0.037	13.756	1.1E-42	intronic	*PNPLA3*	.	.	.
22	44325631	rs12484809	C	T	0.507	0.037	13.756	1.1E-42	intronic	*PNPLA3*	.	.	.
22	44325565	rs12484801	C	T	0.507	0.037	13.753	1.1E-42	intronic	*PNPLA3*	.	.	.
22	44326272	rs9625962	T	C	0.504	0.037	13.701	2.3E-42	intronic	*PNPLA3*	.	.	.
22	44325996	rs12483959	G	A	0.504	0.037	13.701	2.3E-42	intronic	*PNPLA3*	.	.	.
22	44326700	rs11090617	C	T	0.504	0.037	13.695	2.4E-42	intronic	*PNPLA3*	.	.	.
19	19366632	rs72999033	C	T	0.760	0.056	13.677	3.1E-42	UTR3	*HAPLN4*	NM_023002:c.*1994G>A	.	.
22	44332888	rs36038527	T	TC	0.493	0.036	13.670	3.4E-42	intronic	*PNPLA3*	.	.	.
22	44336957	rs73176497	G	A	0.493	0.036	13.670	3.4E-42	intronic	*PNPLA3*	.	.	.
22	44332570	rs2281135	G	A	0.493	0.036	13.669	3.4E-42	intronic	*PNPLA3*	.	.	.
22	44341193	rs4823179	T	C	0.493	0.036	13.663	3.7E-42	intronic	*PNPLA3*	.	.	.
22	44332878	rs34879941	C	T	0.493	0.036	13.662	3.8E-42	intronic	*PNPLA3*	.	.	.
22	44335331	rs16991175	T	C	0.493	0.036	13.659	4.0E-42	intronic	*PNPLA3*	.	.	.
22	44335406	rs35621602	C	A	0.493	0.036	13.659	4.0E-42	intronic	*PNPLA3*	.	.	.
22	44335416	rs34352134	C	T	0.493	0.036	13.659	4.0E-42	intronic	*PNPLA3*	.	.	.
22	44335453	rs34376930	G	T	0.493	0.036	13.659	4.0E-42	intronic	*PNPLA3*	.	.	.
22	44334486	rs4823177	T	C	0.493	0.036	13.658	4.0E-42	intronic	*PNPLA3*	.	.	.
22	44334529	rs4823178	T	C	0.493	0.036	13.658	4.0E-42	intronic	*PNPLA3*	.	.	.
22	44336098	rs1010023	T	C	0.493	0.036	13.658	4.0E-42	intronic	*PNPLA3*	.	.	.
22	44336310	rs1010022	A	G	0.493	0.036	13.658	4.0E-42	intronic	*PNPLA3*	.	.	.
22	44336496	rs8142145	T	C	0.493	0.036	13.658	4.0E-42	intronic	*PNPLA3*	.	.	.
22	44335744	rs2073081	T	C	0.493	0.036	13.657	4.1E-42	intronic	*PNPLA3*	.	.	.
22	44334476	rs4823176	T	C	0.493	0.036	13.657	4.1E-42	intronic	*PNPLA3*	.	.	.
22	44333172	rs2072906	A	G	0.493	0.036	13.656	4.1E-42	intronic	*PNPLA3*	.	.	.
22	44333479	rs2072905	C	G	0.493	0.036	13.656	4.1E-42	intronic	*PNPLA3*	.	.	.
22	44333694	rs2896019	T	G	0.493	0.036	13.656	4.1E-42	intronic	*PNPLA3*	.	.	.
22	44333945	rs2401512	C	G	0.493	0.036	13.656	4.1E-42	intronic	*PNPLA3*	.	.	.
22	44333968	rs2896020	T	C	0.493	0.036	13.656	4.1E-42	intronic	*PNPLA3*	.	.	.
19	19477877	rs56255430	A	C	0.671	0.049	13.654	4.3E-42	intergenic	*MAU2;GATAD2A*	dist=8314;dist=18775	.	.
22	44339526	rs13056555	C	G	0.492	0.036	13.631	5.8E-42	intronic	*PNPLA3*	.	.	.
22	44340086	rs36069781	C	T	0.492	0.036	13.631	5.8E-42	intronic	*PNPLA3*	.	.	.
22	44340922	rs2294916	T	G	0.492	0.036	13.627	6.1E-42	intronic	*PNPLA3*	.	.	.
22	44341298	rs4823180	G	A	0.492	0.036	13.627	6.1E-42	intronic	*PNPLA3*	.	.	.
22	44341606	rs4823181	T	C	0.492	0.036	13.627	6.1E-42	intronic	*PNPLA3*	.	.	.
22	44331943	rs1883349	G	A	0.493	0.036	13.621	6.6E-42	intronic	*PNPLA3*	.	.	.
22	44337533	rs926633	G	A	0.491	0.036	13.612	7.5E-42	intronic	*PNPLA3*	.	.	.
22	44331513	rs1997693	C	G	0.493	0.036	13.600	8.9E-42	intronic	*PNPLA3*	.	.	.
19	19702384	rs17217098	G	A	0.727	0.054	13.575	1.2E-41	intronic	*PBX4*	.	.	.
22	44342969	rs2008451	T	C	0.489	0.036	13.555	1.6E-41	UTR3	*PNPLA3*	NM_025225:c.*707T>C	.	.
22	44343151	rs1810508	A	G	0.489	0.036	13.555	1.6E-41	UTR3	*PNPLA3*	NM_025225:c.*889A>G	.	.
22	44334842	rs2281293	T	C	0.489	0.036	13.546	1.8E-41	intronic	*PNPLA3*	.	.	.
22	44333370	rs2076207	A	G	0.489	0.036	13.543	1.9E-41	intronic	*PNPLA3*	.	.	.
22	44332653	rs2072907	C	G	0.488	0.036	13.540	2.0E-41	intronic	*PNPLA3*	.	.	.
22	44332477	rs2281138	T	C	0.488	0.036	13.538	2.0E-41	intronic	*PNPLA3*	.	.	.

Table continues on next page

CHROM	POS	ID	REF	ALT	BETA	SE	T_STAT	P	Func.refGene	Gene.refGene	GeneDetail.refGene	ExonicFunc.refGene	AAChange.refGene
22	44345771	rs13054885	G	A	0.486	0.036	13.536	2.1E-41	intergenic	*PNPLA3;SAMM50*	dist=2309;dist=5551	.	.
22	44331778	rs13056638	C	G	0.490	0.036	13.536	2.1E-41	intronic	*PNPLA3*	.	.	.
22	44332493	rs2281137	T	C	0.488	0.036	13.535	2.1E-41	intronic	*PNPLA3*	.	.	.
22	44331815	rs1883348	C	G	0.490	0.036	13.533	2.2E-41	intronic	*PNPLA3*	.	.	.
22	44341666	rs13055900	A	G	0.488	0.036	13.514	2.8E-41	intronic	*PNPLA3*	.	.	.
22	44341672	rs13055874	T	C	0.488	0.036	13.514	2.8E-41	intronic	*PNPLA3*	.	.	.
22	44329275	rs2294433	G	A	0.496	0.037	13.471	5.0E-41	intronic	*PNPLA3*	.	.	.
22	44330031	rs1977080	C	T	0.494	0.037	13.448	6.7E-41	intronic	*PNPLA3*	.	.	.
22	44328730	rs4823173	G	A	0.495	0.037	13.433	8.2E-41	intronic	*PNPLA3*	.	.	.
22	44329078	rs2076211	C	T	0.495	0.037	13.433	8.2E-41	intronic	*PNPLA3*	.	.	.
22	44327179	rs16991158	G	A	0.494	0.037	13.421	9.6E-41	intronic	*PNPLA3*	.	.	.
22	44327192	rs36055245	A	G	0.494	0.037	13.418	1.0E-40	intronic	*PNPLA3*	.	.	.
19	19717056	rs73004967	A	G	0.718	0.054	13.416	1.0E-40	intronic	*PBX4*	.	.	.
22	44330128	rs1977081	T	C	0.491	0.037	13.356	2.3E-40	intronic	*PNPLA3*	.	.	.
19	19699398	19:19699398_GA_G	GA	G	0.719	0.054	13.332	3.1E-40	.	.	.	.	.
22	44327273	rs12484700	A	G	0.490	0.037	13.318	3.8E-40	intronic	*PNPLA3*	.	.	.
22	44347251	rs2092501	G	A	0.491	0.037	13.242	1.0E-39	intergenic	*PNPLA3;SAMM50*	dist=3789;dist=4071	.	.
22	44348446	rs34912062	G	T	0.489	0.037	13.207	1.6E-39	intergenic	*PNPLA3;SAMM50*	dist=4984;dist=2876	.	.
22	44349236	rs1474745	T	C	0.484	0.037	13.129	4.4E-39	intergenic	*PNPLA3;SAMM50*	dist=5774;dist=2086	.	.
22	44356468	rs56373884	G	A	0.486	0.037	13.100	6.5E-39	intronic	*SAMM50*	.	.	.
19	19610596	rs3794991	C	T	0.642	0.049	13.062	1.1E-38	intronic	*GATAD2A*	.	.	.
19	19578743	rs73002956	A	G	0.642	0.049	13.004	2.2E-38	intronic	*GATAD2A*	.	.	.
22	44361842	rs2294921	C	T	0.480	0.037	12.998	2.4E-38	intronic	*SAMM50*	.	.	.
22	44343626	rs12484795	A	C	0.464	0.036	12.976	3.3E-38	downstream	*PNPLA3*	dist=164	.	.
22	44368122	rs3761472	A	G	0.478	0.037	12.968	3.6E-38	exonic	*SAMM50*	.	nonsynonymous SNV	SAMM50:NM_015380:exon5:c.A329G:p.D110G
19	19662220	rs17216525	C	T	0.648	0.050	12.954	4.3E-38	intergenic	*CILP2;PBX4*	dist=4752;dist=10302	.	.
19	19658472	rs16996148	G	T	0.644	0.050	12.936	5.4E-38	intergenic	*CILP2;PBX4*	dist=1004;dist=14050	.	.
19	19667254	rs143988316	C	T	0.643	0.050	12.909	7.6E-38	intergenic	*CILP2;PBX4*	dist=9786;dist=5268	.	.
19	19664077	rs17216588	C	T	0.638	0.050	12.785	3.7E-37	intergenic	*CILP2;PBX4*	dist=6609;dist=8445	.	.
19	19670610	rs150824230	G	A	0.633	0.050	12.707	9.9E-37	intergenic	*CILP2;PBX4*	dist=13142;dist=1912	.	.
19	19671266	rs73004926	C	T	0.635	0.050	12.703	1.0E-36	intergenic	*CILP2;PBX4*	dist=13798;dist=1256	.	.
19	19675696	rs73004933	C	T	0.634	0.050	12.699	1.1E-36	intronic	*PBX4*	.	.	.
19	19685470	rs141756246	G	GT	0.631	0.050	12.616	3.1E-36	intronic	*PBX4*	.	.	.
19	19713069	rs73004962	A	T	0.619	0.049	12.535	8.5E-36	intronic	*PBX4*	.	.	.
19	19329924	rs2228603	C	T	0.645	0.051	12.529	9.1E-36	exonic	*NCAN*	.	nonsynonymous SNV	NCAN:NM_004386:exon3:c.C274T:p.P92S
19	19721722	rs12610185	G	A	0.616	0.049	12.487	1.5E-35	intronic	*PBX4*	.	.	.
19	19721976	rs12610191	C	T	0.616	0.049	12.487	1.5E-35	intronic	*PBX4*	.	.	.
19	19695228	rs73004951	C	T	0.618	0.049	12.478	1.7E-35	intronic	*PBX4*	.	.	.
19	19726022	rs58847337	G	A	0.614	0.049	12.462	2.1E-35	intronic	*PBX4*	.	.	.
19	19720399	rs57504626	C	T	0.614	0.049	12.460	2.1E-35	intronic	*PBX4*	.	.	.
19	19720788	rs16996185	T	G	0.614	0.049	12.460	2.1E-35	intronic	*PBX4*	.	.	.
19	19723215	rs10500212	C	T	0.614	0.049	12.460	2.1E-35	intronic	*PBX4*	.	.	.
19	19716558	rs73004966	C	T	0.614	0.049	12.460	2.2E-35	intronic	*PBX4*	.	.	.
19	19700552	rs12608729	C	T	0.614	0.049	12.456	2.3E-35	intronic	*PBX4*	.	.	.
19	19711139	rs73004959	C	T	0.616	0.049	12.451	2.4E-35	intronic	*PBX4*	.	.	.
19	19727152	rs73004975	A	G	0.611	0.049	12.406	4.2E-35	intronic	*PBX4*	.	.	.
22	44381482	rs4823109	C	T	0.461	0.039	11.894	2.0E-32	intronic	*SAMM50*	.	.	.
22	44378672	rs4823183	C	A	0.460	0.039	11.889	2.1E-32	intronic	*SAMM50*	.	.	.
22	44378809	rs2235777	C	T	0.460	0.039	11.889	2.1E-32	intronic	*SAMM50*	.	.	.
22	44381340	rs4823108	T	C	0.461	0.039	11.883	2.3E-32	intronic	*SAMM50*	.	.	.
22	44371406	rs61473277	A	G	0.459	0.039	11.880	2.4E-32	intronic	*SAMM50*	.	.	.
22	44377999	rs2235776	C	T	0.459	0.039	11.868	2.8E-32	intronic	*SAMM50*	.	.	.
22	44380170	rs71313378	G	GCTTC	0.460	0.039	11.867	2.8E-32	intronic	*SAMM50*	.	.	.
22	44380767	rs12167845	T	C	0.459	0.039	11.864	2.9E-32	intronic	*SAMM50*	.	.	.
22	44379740	rs2294923	C	A	0.458	0.039	11.853	3.3E-32	intronic	*SAMM50*	.	.	.
22	44380009	rs9626079	A	G	0.458	0.039	11.853	3.3E-32	intronic	*SAMM50*	.	.	.
19	19746151	rs2304128	G	T	0.597	0.051	11.675	2.7E-31	intronic	*GMIP*	.	.	.
19	19425025	rs57962361	C	T	0.462	0.043	10.726	1.1E-26	intronic	*SUGP1*	.	.	.
19	19436229	rs11234557	C	G	0.460	0.043	10.683	1.6E-26	intronic	*MAU2*	.	.	.
19	19426181	rs11668104	G	A	0.457	0.043	10.633	2.8E-26	intronic	*SUGP1*	.	.	.

Table continues next page

CHROM	POS	ID	REF	ALT	BETA	SE	T_STAT	P	Func.refGene	Gene.refGene	GeneDetail.refGene	ExonicFunc.refGene	AAChange.refGene
19	19462702	rs11672355	G	C	0.457	0.043	10.625	3.1E-26	intronic	*MAU2*	.	.	.
19	19440064	rs11411903	T	TA	0.456	0.043	10.605	3.8E-26	intronic	*MAU2*	.	.	.
19	19756073	19:19756073_AGCC_A	AGCC	A	0.569	0.055	10.404	3.1E-25	.	.	.	.	.
19	19406869	rs12979148	T	C	0.422	0.041	10.375	4.2E-25	intronic	*SUGP1*	.	.	.
19	19418916	rs2240117	C	T	0.418	0.041	10.264	1.3E-24	intronic	*SUGP1*	.	.	.
19	19450254	19:19450254_CA_C	CA	C	0.429	0.043	10.041	1.3E-23	.	.	.	.	.
19	19793545	rs56408111	T	C	0.499	0.050	10.009	1.8E-23	UTR3	*ZNF101*	NM_001300949:c.*2436T>C;NM_033204:c.*2436T>C	.	.
19	19786099	rs58434384	A	G	0.481	0.048	9.927	4.0E-23	intronic	*ZNF101*	.	.	.
19	19789528	rs2304130	A	G	0.479	0.048	9.902	5.1E-23	intronic	*ZNF101*	.	.	.
19	19485105	rs12052117	C	T	0.409	0.042	9.774	1.8E-22	intergenic	*MAU2;GATAD2A*	dist=15542;dist=11547	.	.
19	19582992	rs73002960	C	T	0.407	0.042	9.708	3.4E-22	intronic	*GATAD2A*	.	.	.
19	19325963	rs3761077	G	T	0.423	0.044	9.701	3.7E-22	intronic	*NCAN*	.	.	.
19	19572220	rs28720066	G	T	0.406	0.042	9.686	4.2E-22	intronic	*GATAD2A*	.	.	.
19	19467545	rs2285626	C	T	0.399	0.041	9.671	4.9E-22	UTR3	*MAU2*	NM_015329:c.*954C>T	.	.
19	19484008	rs59148799	A	G	0.404	0.042	9.668	5.0E-22	intergenic	*MAU2;GATAD2A*	dist=14445;dist=12644	.	.
19	19621004	rs56273306	T	C	0.404	0.042	9.664	5.2E-22	intergenic	*GATAD2A;TSSK6*	dist=1263;dist=4024	.	.
19	19506092	rs56241616	C	T	0.404	0.042	9.658	5.6E-22	intronic	*GATAD2A*	.	.	.
19	19539891	rs8182472	T	C	0.404	0.042	9.658	5.6E-22	intronic	*GATAD2A*	.	.	.
19	19503573	rs10408875	T	C	0.404	0.042	9.656	5.7E-22	intronic	*GATAD2A*	.	.	.
19	19512657	rs10408596	A	T	0.404	0.042	9.655	5.7E-22	intronic	*GATAD2A*	.	.	.
19	19505087	rs10415849	C	T	0.403	0.042	9.645	6.3E-22	intronic	*GATAD2A*	.	.	.
19	19513568	rs34324111	T	G	0.401	0.042	9.597	1.0E-21	intronic	*GATAD2A*	.	.	.
19	19513572	rs35629458	T	G	0.401	0.042	9.597	1.0E-21	intronic	*GATAD2A*	.	.	.
19	19513580	rs113460678	T	G	0.401	0.042	9.597	1.0E-21	intronic	*GATAD2A*	.	.	.
19	19665581	rs34755166	G	A	0.394	0.041	9.577	1.2E-21	intergenic	*CILP2;PBX4*	dist=8113;dist=6941	.	.
19	19666574	rs17216693	T	C	0.392	0.041	9.560	1.4E-21	intergenic	*CILP2;PBX4*	dist=9106;dist=5948	.	.
22	44391234	rs2281298	G	A	0.336	0.035	9.559	1.4E-21	intronic	*SAMM50*	.	.	.
22	44391686	rs2143571	G	A	0.336	0.035	9.557	1.5E-21	intronic	*SAMM50*	.	.	.
19	19642795	rs56397647	C	T	0.403	0.042	9.495	2.7E-21	intronic	*YJEFN3*	.	.	.
22	44394019	rs2401514	T	A	0.331	0.035	9.442	4.4E-21	intergenic	*SAMM50;PARVB*	dist=1610;dist=1072	.	.
22	44385594	rs2073079	A	G	0.330	0.035	9.429	5.0E-21	intronic	*SAMM50*	.	.	.
22	44394402	rs2073080	C	T	0.330	0.035	9.418	5.5E-21	upstream	*PARVB*	dist=689	.	.
22	44388817	rs3827385	T	C	0.328	0.035	9.401	6.5E-21	intronic	*SAMM50*	.	.	.
19	19336608	rs2238675	C	T	0.379	0.041	9.260	2.4E-20	intronic	*NCAN*	.	.	.
19	19531910	rs11668386	A	G	0.376	0.041	9.131	8.0E-20	intronic	*GATAD2A*	.	.	.
19	19393677	rs35431065	A	G	0.340	0.037	9.119	8.9E-20	intronic	*SUGP1*	.	.	.
19	19508013	rs10424702	A	G	0.371	0.041	9.057	1.6E-19	intronic	*GATAD2A*	.	.	.
19	19548643	rs79954596	T	G	0.370	0.041	9.034	1.9E-19	intronic	*GATAD2A*	.	.	.
19	19517169	rs188552254	A	G	0.370	0.041	9.031	2.0E-19	intronic	*GATAD2A*	.	.	.
19	19668338	19:19668338_GC_G	GC	G	0.372	0.041	9.029	2.0E-19	.	.	.	.	.
19	19621197	rs113365218	G	A	0.379	0.042	9.025	2.1E-19	intergenic	*GATAD2A;TSSK6*	dist=1456;dist=3831	.	.
22	44376335	rs67450864	C	T	0.260	0.029	8.958	3.8E-19	intronic	*SAMM50*	.	.	.
22	44377442	rs4823182	A	G	0.259	0.029	8.951	4.1E-19	intronic	*SAMM50*	.	.	.
19	19613622	rs57009615	A	G	0.356	0.040	8.825	1.3E-18	intronic	*GATAD2A*	.	.	.
22	44361713	rs12165526	T	A	0.410	0.047	8.810	1.4E-18	intronic	*SAMM50*	.	.	.
22	44387932	rs10656207	C	CTA	0.245	0.028	8.767	2.1E-18	intronic	*SAMM50*	.	.	.
22	44335670	22:44335670_TGG_T	TGG	T	0.239	0.027	8.730	2.9E-18	.	.	.	.	.
22	44395451	rs1007863	T	C	0.243	0.028	8.708	3.5E-18	exonic	*PARVB*	.	nonsynonymous SNV	PARVB:NM_001003828:exon2:c.T109C:p.W37R
22	44383400	rs6006602	C	T	0.242	0.028	8.657	5.5E-18	intronic	*SAMM50*	.	.	.
22	44383432	rs6006468	G	C	0.242	0.028	8.657	5.5E-18	intronic	*SAMM50*	.	.	.
22	44382684	rs2294927	T	C	0.243	0.028	8.652	5.8E-18	intronic	*SAMM50*	.	.	.
22	44389514	rs2235778	T	C	0.242	0.028	8.647	6.0E-18	intronic	*SAMM50*	.	.	.
22	44393075	rs6006473	C	T	0.241	0.028	8.644	6.2E-18	downstream	*SAMM50*	dist=666	.	.
22	44383617	rs6006469	C	G	0.241	0.028	8.628	7.1E-18	intronic	*SAMM50*	.	.	.
22	44395389	rs2281292	A	C	0.240	0.028	8.626	7.2E-18	intronic	*PARVB*	.	.	.
22	44388417	rs3788604	A	G	0.240	0.028	8.611	8.2E-18	intronic	*SAMM50*	.	.	.
22	44387108	rs1986095	A	G	0.240	0.028	8.606	8.6E-18	intronic	*SAMM50*	.	.	.
22	44358997	rs16991236	A	G	0.446	0.052	8.542	1.5E-17	intronic	*SAMM50*	.	.	.
22	44328043	rs1883350	T	C	0.254	0.030	8.366	6.7E-17	intronic	*PNPLA3*	.	.	.
19	19155672	rs73008942	G	A	0.484	0.062	7.870	3.9E-15	intronic	*ARMC6*	.	.	.

Table continues on next page

CHROM	POS	ID	REF	ALT	BETA	SE	T_STAT	P	Func.refGene	Gene.refGene	GeneDetail.refGene	ExonicFunc.refGene	AAChange.refGene
19	19110422	rs73006914	C	T	0.487	0.062	7.848	4.6E-15	intronic	*SUGP2*	.	.	.
19	19207229	rs80341032	G	A	0.461	0.060	7.740	1.1E-14	intronic	*SLC25A42*	.	.	.
19	19434042	rs58833986	T	TCACCA	0.276	0.036	7.640	2.4E-14	intronic	*MAU2*	.	.	.
19	19103986	rs3810444	T	A	0.449	0.059	7.633	2.5E-14	UTR3	*SUGP2*	NM_001321699:c.*564A>T;NM_001352071:c.*564A>T;NM_001017392:c.*564A>T	.	.
19	19374061	rs8105094	C	T	0.281	0.037	7.630	2.5E-14	upstream	*HAPLN4*	dist=448	.	.
19	19374068	rs8105984	T	C	0.281	0.037	7.630	2.5E-14	upstream	*HAPLN4*	dist=455	.	.
22	44327075	rs9626056	C	T	0.434	0.057	7.564	4.2E-14	intronic	*PNPLA3*	.	.	.
22	44328075	rs9306471	A	G	0.433	0.057	7.526	5.6E-14	intronic	*PNPLA3*	.	.	.
19	19405480	rs2023883	G	A	0.269	0.036	7.482	7.9E-14	intronic	*SUGP1*	.	.	.
19	19407171	rs11666553	C	A	0.269	0.036	7.475	8.3E-14	intronic	*SUGP1*	.	.	.
19	19422152	19:19422152_CA_C	CA	C	0.270	0.036	7.475	8.3E-14	.	.	.	.	.
19	19425141	rs12459676	A	T	0.268	0.036	7.465	8.9E-14	intronic	*SUGP1*	.	.	.
19	19651577	rs12981405	C	T	0.276	0.037	7.464	9.1E-14	intronic	*CILP2*	.	.	.
19	19381715	rs2074301	G	A	0.269	0.036	7.464	9.1E-14	intronic	*TM6SF2*	.	.	.
19	19467996	rs2285628	T	A	0.268	0.036	7.443	1.1E-13	UTR3	*MAU2*	NM_015329:c.*1405T>A	.	.
19	19560756	rs775175628	TATCTTATATTAA	T	0.272	0.037	7.440	1.1E-13	.	.	.	.	.
19	19380996	rs2074300	G	T	0.268	0.036	7.434	1.1E-13	exonic	*TM6SF2*	.	synonymous SNV	TM6SF2:NM_001001524:exon4:c.C387A:p.G129G
19	19471241	rs10419672	T	C	0.266	0.036	7.414	1.3E-13	intergenic	*MAU2;GATAD2A*	dist=1678;dist=25411	.	.
22	44329719	rs111393709	C	T	0.426	0.057	7.414	1.3E-13	intronic	*PNPLA3*	.	.	.
19	19419810	rs12983137	G	A	0.265	0.036	7.407	1.4E-13	intronic	*SUGP1*	.	.	.
19	19422187	rs1859287	T	C	0.265	0.036	7.407	1.4E-13	intronic	*SUGP1*	.	.	.
19	19428805	rs7259434	A	T	0.265	0.036	7.407	1.4E-13	intronic	*SUGP1*	.	.	.
19	19432959	rs10402661	A	G	0.265	0.036	7.407	1.4E-13	intronic	*MAU2*	.	.	.
19	19429220	rs12976025	C	T	0.265	0.036	7.407	1.4E-13	intronic	*SUGP1*	.	.	.
19	19435680	rs4808194	T	G	0.265	0.036	7.407	1.4E-13	intronic	*MAU2*	.	.	.
19	19469296	rs10403731	G	A	0.266	0.036	7.402	1.4E-13	UTR3	*MAU2*	NM_015329:c.*2705G>A	.	.
19	19462606	rs11085261	G	A	0.265	0.036	7.394	1.5E-13	intronic	*MAU2*	.	.	.
19	19374546	rs563530889	A	AAG	0.267	0.036	7.393	1.5E-13	upstream;downstream	*HAPLN4;TM6SF2*	dist=933;dist=630	.	.
19	19445856	rs11085259	C	T	0.265	0.036	7.387	1.6E-13	intronic	*MAU2*	.	.	.
19	19427623	rs8108647	A	G	0.264	0.036	7.386	1.6E-13	intronic	*SUGP1*	.	.	.
19	19448301	rs757000	A	G	0.265	0.036	7.386	1.6E-13	intronic	*MAU2*	.	.	.
19	19448808	rs757001	G	A	0.265	0.036	7.386	1.6E-13	intronic	*MAU2*	.	.	.
19	19452249	rs2301668	G	A	0.265	0.036	7.386	1.6E-13	intronic	*MAU2*	.	.	.
19	19459800	rs12982276	T	C	0.264	0.036	7.376	1.8E-13	intronic	*MAU2*	.	.	.
19	19423083	rs756264087	CAAT	C	0.264	0.036	7.370	1.8E-13	.	.	.	.	.
19	19459554	rs10421505	C	T	0.264	0.036	7.368	1.9E-13	intronic	*MAU2*	.	.	.
19	19475469	rs7258508	T	C	0.263	0.036	7.354	2.1E-13	intergenic	*MAU2;GATAD2A*	dist=5906;dist=21183	.	.
19	19459215	rs968525	C	T	0.263	0.036	7.343	2.2E-13	intronic	*MAU2*	.	.	.
22	44324558	rs55768287	C	T	0.430	0.059	7.341	2.3E-13	intronic	*PNPLA3*	.	.	.
22	44341986	rs2294917	T	C	-0.211	0.029	-7.324	2.6E-13	intronic	*PNPLA3*	.	.	.
19	19431420	19:19431420_GT_G	GT	G	0.285	0.039	7.320	2.7E-13	.	.	.	.	.
19	19465529	rs9304960	G	A	0.261	0.036	7.279	3.6E-13	intronic	*MAU2*	.	.	.
19	19466269	rs2301671	C	T	0.260	0.036	7.272	3.8E-13	intronic	*MAU2*	.	.	.
19	19393106	19:19393106_CAAGA_C	CAAGA	C	0.263	0.036	7.264	4.0E-13	.	.	.	.	.
19	19476520	rs4808196	G	A	0.259	0.036	7.223	5.4E-13	intergenic	*MAU2;GATAD2A*	dist=6957;dist=20132	.	.
22	44383070	rs12166587	T	C	0.383	0.053	7.189	6.9E-13	intronic	*SAMM50*	.	.	.
22	44383502	rs9625970	T	C	0.383	0.053	7.188	7.0E-13	intronic	*SAMM50*	.	.	.
22	44385583	rs112902984	T	C	0.383	0.053	7.182	7.3E-13	intronic	*SAMM50*	.	.	.
22	44387298	rs117472787	C	T	0.383	0.053	7.182	7.3E-13	intronic	*SAMM50*	.	.	.
22	44354111	rs738491	C	T	0.218	0.030	7.166	8.2E-13	intronic	*SAMM50*	.	.	.
19	19397789	rs10418051	C	T	0.252	0.035	7.155	8.9E-13	intronic	*SUGP1*	.	.	.
19	19579241	rs11404084	T	TA	0.255	0.036	7.139	1.0E-12	intronic	*GATAD2A*	.	.	.
22	44393476	rs8141950	C	T	0.381	0.053	7.119	1.2E-12	intergenic	*SAMM50;PARVB*	dist=1067;dist=1615	.	.
19	19588546	rs1465695	A	C	0.253	0.036	7.095	1.4E-12	intronic	*GATAD2A*	.	.	.
19	19595014	rs10404728	C	T	0.253	0.036	7.095	1.4E-12	intronic	*GATAD2A*	.	.	.
19	19602821	rs751858	G	C	0.253	0.036	7.094	1.4E-12	intronic	*GATAD2A*	.	.	.
19	19504167	rs17288409	T	C	0.253	0.036	7.093	1.4E-12	intronic	*GATAD2A*	.	.	.
19	19591066	rs10401193	A	G	0.253	0.036	7.093	1.4E-12	intronic	*GATAD2A*	.	.	.
22	44338049	rs9625965	T	C	0.388	0.055	7.091	1.4E-12	intronic	*PNPLA3*	.	.	.
19	19578890	rs754255	T	C	0.253	0.036	7.085	1.5E-12	intronic	*GATAD2A*	.	.	.

Table continues on next page

CHROM	POS	ID	REF	ALT	BETA	SE	T_STAT	P	Func.refGene	Gene.refGene	GeneDetail.refGene	ExonicFunc.refGene	AAChange.refGene
19	19557353	rs60003758	A	G	0.253	0.036	7.083	1.5E-12	intronic	*GATAD2A*	.	.	.
22	44338105	rs9625966	C	T	0.387	0.055	7.083	1.5E-12	intronic	*PNPLA3*	.	.	.
22	44337610	rs9625964	G	A	0.387	0.055	7.081	1.5E-12	intronic	*PNPLA3*	.	.	.
19	19562349	rs12972397	G	A	0.252	0.036	7.076	1.6E-12	intronic	*GATAD2A*	.	.	.
22	44339791	rs9626057	C	G	0.385	0.055	7.055	1.8E-12	intronic	*PNPLA3*	.	.	.
19	19499598	rs60321073	A	G	0.252	0.036	7.044	2.0E-12	intronic	*GATAD2A*	.	.	.
19	19605963	rs2099333	C	T	0.252	0.036	7.040	2.0E-12	intronic	*GATAD2A*	.	.	.
22	44357894	rs56219234	G	T	0.215	0.031	7.036	2.1E-12	intronic	*SAMM50*	.	.	.
19	19283268	rs61061000	C	T	0.305	0.043	7.031	2.2E-12	intronic	*BORCS8-MEF2B*	.	.	.
19	19568244	rs12977524	A	G	0.251	0.036	7.026	2.2E-12	intronic	*GATAD2A*	.	.	.
19	19545099	rs4808199	G	A	0.251	0.036	7.025	2.3E-12	intronic	*GATAD2A*	.	.	.
22	44339055	rs117772800	A	G	0.383	0.055	7.024	2.3E-12	intronic	*PNPLA3*	.	.	.
19	19571100	rs7250658	A	G	0.251	0.036	7.020	2.4E-12	intronic	*GATAD2A*	.	.	.
19	19582651	rs6511036	A	G	0.251	0.036	7.014	2.5E-12	intronic	*GATAD2A*	.	.	.
19	19628037	rs7252888	G	A	0.250	0.036	7.013	2.5E-12	intronic	*NDUFA13*	.	.	.
19	19575945	rs2163805	G	A	0.251	0.036	7.011	2.5E-12	intronic	*GATAD2A*	.	.	.
19	19574277	rs4808960	G	C	0.251	0.036	7.009	2.5E-12	intronic	*GATAD2A*	.	.	.
19	19575965	rs2163804	G	A	0.251	0.036	7.008	2.6E-12	intronic	*GATAD2A*	.	.	.
19	19510831	19:19510831_AT_A	AT	A	0.250	0.036	6.979	3.2E-12	.	.	.	.	.
22	44370439	rs738494	A	C	0.375	0.054	6.924	4.6E-12	intronic	*SAMM50*	.	.	.
22	44344872	rs9626061	C	T	0.378	0.055	6.915	4.9E-12	intergenic	*PNPLA3;SAMM50*	dist=1410;dist=6450	.	.
22	44359938	rs9626071	C	A	0.373	0.054	6.898	5.6E-12	intronic	*SAMM50*	.	.	.
22	44397144	rs8141994	A	G	0.371	0.054	6.897	5.6E-12	intronic	*PARVB*	.	.	.
22	44359651	rs12168138	T	C	0.373	0.054	6.892	5.8E-12	intronic	*SAMM50*	.	.	.
22	44358819	rs28754570	G	C	0.373	0.054	6.891	5.8E-12	intronic	*SAMM50*	.	.	.
22	44359729	rs12170274	C	T	0.373	0.054	6.891	5.8E-12	intronic	*SAMM50*	.	.	.
22	44356562	rs28421169	T	C	0.372	0.054	6.885	6.1E-12	intronic	*SAMM50*	.	.	.
22	44343352	rs9626058	A	G	0.375	0.054	6.877	6.4E-12	UTR3	*PNPLA3*	NM_025225:c.*1090A>G	.	.
22	44382004	rs6006599	C	A	0.209	0.031	6.857	7.4E-12	intronic	*SAMM50*	.	.	.
22	44368584	rs9626076	G	A	0.371	0.054	6.843	8.1E-12	intronic	*SAMM50*	.	.	.
22	44366135	rs12168183	G	A	0.371	0.054	6.842	8.3E-12	intronic	*SAMM50*	.	.	.
22	44366874	rs9626075	G	A	0.371	0.054	6.837	8.5E-12	intronic	*SAMM50*	.	.	.
22	44353447	rs28733632	C	T	0.371	0.054	6.836	8.6E-12	intronic	*SAMM50*	.	.	.
22	44348284	rs117369516	C	T	0.372	0.054	6.834	8.7E-12	intergenic	*PNPLA3;SAMM50*	dist=4822;dist=3038	.	.
22	44354598	rs9626064	C	T	0.371	0.054	6.830	8.9E-12	intronic	*SAMM50*	.	.	.
22	44363736	rs9626074	C	T	0.371	0.054	6.829	9.0E-12	intronic	*SAMM50*	.	.	.
22	44369927	rs12330016	C	T	0.370	0.054	6.827	9.1E-12	intronic	*SAMM50*	.	.	.
22	44371030	rs117130990	G	A	0.370	0.054	6.827	9.1E-12	intronic	*SAMM50*	.	.	.
22	44362178	rs9626073	G	A	0.370	0.054	6.826	9.2E-12	intronic	*SAMM50*	.	.	.
22	44370955	rs73434655	A	G	0.370	0.054	6.826	9.2E-12	intronic	*SAMM50*	.	.	.
22	44344011	rs12167852	A	G	0.372	0.055	6.820	9.6E-12	downstream	*PNPLA3*	dist=549	.	.
22	44342691	rs41278873	T	C	0.364	0.054	6.809	1.0E-11	UTR3	*PNPLA3*	NM_025225:c.*429T>C	.	.
22	44373947	rs9626078	G	A	0.369	0.054	6.808	1.0E-11	intronic	*SAMM50*	.	.	.
19	19531175	rs28478453	C	G	0.241	0.035	6.802	1.1E-11	intronic	*GATAD2A*	.	.	.
22	44357928	rs9626065	A	C	0.369	0.054	6.800	1.1E-11	intronic	*SAMM50*	.	.	.
22	44357940	rs9626066	T	A	0.368	0.054	6.798	1.1E-11	intronic	*SAMM50*	.	.	.
22	44358030	rs9626067	C	T	0.368	0.054	6.797	1.1E-11	intronic	*SAMM50*	.	.	.
22	44377221	rs75439392	G	T	0.368	0.054	6.793	1.2E-11	intronic	*SAMM50*	.	.	.
22	44358360	rs9626068	C	G	0.368	0.054	6.793	1.2E-11	intronic	*SAMM50*	.	.	.
22	44348116	rs11912828	G	A	-0.211	0.031	-6.789	1.2E-11	intergenic	*PNPLA3;SAMM50*	dist=4654;dist=3206	.	.
22	44342325	rs2294919	C	T	-0.213	0.031	-6.782	1.2E-11	UTR3	*PNPLA3*	NM_025225:c.*63C>T	.	.
19	19461416	rs565756573	C	CA	0.258	0.038	6.777	1.3E-11	intronic	*MAU2*	.	.	.
19	19294392	rs113954889	T	A	0.300	0.044	6.772	1.3E-11	intronic	*BORCS8;BORCS8-MEF2B*	.	.	.
19	19657500	rs10402308	G	A	0.244	0.036	6.769	1.4E-11	downstream	*CILP2*	dist=32	.	.
22	44347504	rs12170782	C	A	0.368	0.055	6.738	1.7E-11	intergenic	*PNPLA3;SAMM50*	dist=4042;dist=3818	.	.
19	19532682	rs11669516	G	A	0.237	0.035	6.717	2.0E-11	intronic	*GATAD2A*	.	.	.
22	44327012	rs139052	A	C	-0.214	0.032	-6.706	2.1E-11	intronic	*PNPLA3*	.	.	.
19	19548239	rs34647936	T	G	0.236	0.035	6.691	2.3E-11	intronic	*GATAD2A*	.	.	.
19	19516431	rs12983940	G	A	0.237	0.035	6.689	2.4E-11	intronic	*GATAD2A*	.	.	.
19	19488718	rs12973258	T	C	0.235	0.035	6.659	2.9E-11	intergenic	*MAU2;GATAD2A*	dist=19155;dist=7934	.	.

Table continues on next page

CHROM	POS	ID	REF	ALT	BETA	SE	T_STAT	P	Func.refGene	Gene.refGene	GeneDetail.refGene	ExonicFunc.refGene	AAChange.refGene
19	19518889	rs4808950	A	G	0.234	0.035	6.623	3.7E-11	intronic	*GATAD2A*	.	.	.
19	19314526	rs8100140	G	A	0.186	0.028	6.548	6.1E-11	upstream	*NR2C2AP*	dist=303	.	.
22	44381944	22:44381944_ATGGAGTCTTGCTC_A	ATGGAGTCTTGCTC	A	0.200	0.031	6.466	1.1E-10	.	.	.	.	.
22	44349215	rs1474744	C	T	-0.174	0.027	-6.433	1.3E-10	intergenic	*PNPLA3;SAMM50*	dist=5753;dist=2107	.	.
19	19513570	rs111901094	G	T	0.234	0.036	6.427	1.4E-10	intronic	*GATAD2A*	.	.	.
19	19525792	rs2965185	T	C	0.182	0.030	6.138	8.7E-10	intronic	*GATAD2A*	.	.	.
22	44409993	rs12484530	G	A	0.292	0.048	6.039	1.6E-09	intronic	*PARVB*	.	.	.
19	54671421	rs60204587	G	A	0.167	0.028	5.979	2.3E-09	intronic	*TMC4*	.	.	.
22	44365232	rs6006594	C	G	-0.163	0.027	-5.936	3.0E-09	intronic	*SAMM50*	.	.	.
19	19260760	rs28451834	G	C	0.245	0.042	5.904	3.7E-09	intronic	*BORCS8-MEF2B;MEF2B*	.	.	.
22	44372331	rs2073086	C	T	0.183	0.031	5.899	3.8E-09	intronic	*SAMM50*	.	.	.
19	19743098	rs12609436	C	T	0.161	0.027	5.892	3.9E-09	intronic	*GMIP*	.	.	.
19	54674742	rs4806498	C	T	0.163	0.028	5.887	4.0E-09	intronic	*TMC4*	.	.	.
22	44350417	rs1474746	G	C	-0.161	0.027	-5.873	4.4E-09	upstream	*SAMM50*	dist=905	.	.
19	19738554	rs873870	G	A	0.159	0.027	5.868	4.6E-09	intronic	*LPAR2*	.	.	.
22	44324676	rs139051	G	A	0.168	0.029	5.867	4.6E-09	intronic	*PNPLA3*	.	.	.
22	44343239	rs3083314	CAA	C	-0.160	0.027	-5.807	6.5E-09	.	.	.	.	.
22	44355569	rs2401513	C	T	-0.185	0.032	-5.791	7.2E-09	intronic	*SAMM50*	.	.	.
19	19480521	rs2872878	C	G	0.167	0.029	5.780	7.7E-09	intergenic	*MAU2;GATAD2A*	dist=10958;dist=16131	.	.
22	44372069	rs2073084	G	A	0.179	0.031	5.763	8.5E-09	intronic	*SAMM50*	.	.	.
22	44345926	rs28550680	C	T	-0.185	0.032	-5.761	8.6E-09	intergenic	*PNPLA3;SAMM50*	dist=2464;dist=5396	.	.
19	54676763	rs641738	C	T	0.159	0.028	5.748	9.3E-09	exonic	*TMC4*	.	nonsynonymous SNV	TMC4:NM_001145303:exon1:c.G50A:p.G17E,TMC4:NM_144686:exon1:c.G50A:p.G17E
22	44346128	rs11704562	C	T	-0.184	0.032	-5.748	9.3E-09	intergenic	*PNPLA3;SAMM50*	dist=2666;dist=5194	.	.
22	44346639	rs7289329	T	G	-0.184	0.032	-5.747	9.3E-09	intergenic	*PNPLA3;SAMM50*	dist=3177;dist=4683	.	.
22	44372190	rs2073085	C	T	0.178	0.031	5.742	9.6E-09	intronic	*SAMM50*	.	.	.
22	44354865	rs11705218	A	G	-0.183	0.032	-5.739	9.8E-09	intronic	*SAMM50*	.	.	.
19	19564489	rs36010983	A	AT	0.212	0.037	5.739	9.8E-09	intronic	*GATAD2A*	.	.	.
22	44347433	rs9614293	G	T	-0.184	0.032	-5.739	9.8E-09	intergenic	*PNPLA3;SAMM50*	dist=3971;dist=3889	.	.
19	19377716	rs2074298	C	G	0.166	0.029	5.736	9.9E-09	intronic	*TM6SF2*	.	.	.
22	44372632	rs14315	C	T	0.178	0.031	5.735	1.0E-08	exonic	*SAMM50*	.	synonymous SNV	SAMM50:NM_015380:exon9:c.C780T:p.H260H
19	19670688	rs7249692	C	T	0.166	0.029	5.734	1.0E-08	intergenic	*CILP2;PBX4*	dist=13220;dist=1834	.	.
22	44375742	rs11090620	C	T	0.178	0.031	5.723	1.1E-08	intronic	*SAMM50*	.	.	.
22	44346965	rs5764043	G	A	-0.183	0.032	-5.722	1.1E-08	intergenic	*PNPLA3;SAMM50*	dist=3503;dist=4357	.	.
22	44347250	rs5764045	C	T	-0.183	0.032	-5.722	1.1E-08	intergenic	*PNPLA3;SAMM50*	dist=3788;dist=4072	.	.
19	54677001	rs626283	G	C	0.158	0.028	5.721	1.1E-08	upstream;downstream	*TMC4;MBOAT7*	dist=147;dist=105	.	.
22	44375275	rs2235775	C	A	0.178	0.031	5.720	1.1E-08	intronic	*SAMM50*	.	.	.
22	44347137	rs5764044	C	G	-0.183	0.032	-5.720	1.1E-08	intergenic	*PNPLA3;SAMM50*	dist=3675;dist=4185	.	.
19	19476365	rs2965200	A	G	0.162	0.028	5.717	1.1E-08	intergenic	*MAU2;GATAD2A*	dist=6802;dist=20287	.	.
19	19438110	19:19438110_GTATT_G	GTATT	G	0.186	0.032	5.715	1.1E-08	.	.	.	.	.
19	19679461	rs8103250	G	A	0.165	0.029	5.700	1.2E-08	intronic	*PBX4*	.	.	.
19	19679992	rs9304962	C	G	0.165	0.029	5.700	1.2E-08	intronic	*PBX4*	.	.	.
19	19744452	rs7248200	C	T	0.155	0.027	5.697	1.3E-08	intronic	*GMIP*	.	.	.
19	19392401	rs2269873	T	C	0.165	0.029	5.694	1.3E-08	intronic	*SUGP1*	.	.	.
19	19423817	rs2315024	T	A	0.164	0.029	5.686	1.3E-08	intronic	*SUGP1*	.	.	.
19	19394396	rs10407952	C	T	0.164	0.029	5.683	1.4E-08	intronic	*SUGP1*	.	.	.
19	19426609	rs2315025	C	T	0.163	0.029	5.678	1.4E-08	intronic	*SUGP1*	.	.	.
19	19385411	rs735273	T	C	0.164	0.029	5.674	1.4E-08	intergenic	*TM6SF2;SUGP1*	dist=1301;dist=1431	.	.
19	19386015	rs8103496	A	G	0.164	0.029	5.674	1.4E-08	downstream	*SUGP1*	dist=827	.	.
19	19387149	rs2017964	T	C	0.164	0.029	5.674	1.4E-08	UTR3	*SUGP1*	NM_172231:c.*308A>G	.	.
19	19390497	rs8101938	G	A	0.164	0.029	5.674	1.4E-08	intronic	*SUGP1*	.	.	.
19	19390749	rs2301784	G	A	0.164	0.029	5.674	1.4E-08	intronic	*SUGP1*	.	.	.
19	19391402	rs6511026	C	T	0.164	0.029	5.674	1.4E-08	intronic	*SUGP1*	.	.	.
19	19381755	rs2074303	C	T	0.164	0.029	5.673	1.4E-08	intronic	*TM6SF2*	.	.	.
19	19383755	rs10419245	G	A	0.164	0.029	5.673	1.4E-08	intronic	*TM6SF2*	.	.	.
19	19395967	rs6511028	T	C	0.163	0.029	5.666	1.5E-08	intronic	*SUGP1*	.	.	.
19	19397479	rs7254748	A	C	0.163	0.029	5.664	1.5E-08	intronic	*SUGP1*	.	.	.
19	19398005	rs4808937	A	G	0.163	0.029	5.664	1.5E-08	intronic	*SUGP1*	.	.	.
19	19403410	rs10409234	A	G	0.163	0.029	5.663	1.5E-08	intronic	*SUGP1*	.	.	.
19	19380646	rs2074299	T	C	0.164	0.029	5.663	1.5E-08	intronic	*TM6SF2*	.	.	.
22	44375970	rs56077346	A	G	0.177	0.031	5.663	1.5E-08	intronic	*SAMM50*	.	.	.

Table continues on next page

Table S5. Overlap between the case-control and quantitative GWAS results.

SNP info			Case-control GWAS		Quantitative GWAS		Annotation				
ID	CHROM	POS	OR	P	BETA	P	Func.refGene	Gene.refGene	GeneDetail.refGene	ExonicFunc.refGene	AAChange.refGene
rs738408	22	44324730	1.918	5.4E-30	0.532	4.31E-60	exonic	*PNPLA3*	.	synonymous SNV	PNPLA3:NM_025225:exon3:c.C447T:p.P149P
rs738409	22	44324727	1.917	6.6E-30	0.531	5.29E-60	exonic	*PNPLA3*	.	nonsynonymous SNV	PNPLA3:NM_025225:exon3:c.C444G:p.I148M
rs3747207	22	44324855	1.917	6.9E-30	0.533	7.39E-60	intronic	*PNPLA3*	.	.	.
rs2294915	22	44340904	1.872	1.2E-28	0.510	7.18E-58	intronic	*PNPLA3*	.	.	.
rs200210321	19	19393890	2.386	1.5E-27	0.766	8.92E-49	intronic	*SUGP1*	.	.	.
rs58542926	19	19379549	2.368	1.5E-27	0.752	2.20E-48	exonic	*TM6SF2*	.	nonsynonymous SNV	TM6SF2:NM_001001524:exon6:c.G499A:p.E167K
rs10401969	19	19407718	2.330	8.4E-27	0.748	2.97E-48	intronic	*SUGP1*	.	.	.
rs8107974	19	19388500	2.352	2.5E-27	0.743	1.50E-47	intronic	*SUGP1*	.	.	.
rs756350040	19	19370340	2.393	3.8E-27	0.761	2.46E-47	.	.	.	.	.
rs2294922	22	44379565	1.831	4.8E-25	0.478	7.58E-47	intronic	*SAMM50*	.	.	.
19:19432290_AG_A	19	19432290	2.343	1.0E-24	0.769	8.28E-47	.	.	.	.	.
rs73001065	19	19460541	2.299	5.3E-24	0.755	5.04E-46	intronic	*MAU2*	.	.	.
rs150268548	19	19494483	2.303	2.0E-23	0.768	5.91E-46	intergenic	*MAU2;GATAD2A*	dist=24920;dist=2169	.	.
rs739846	19	19419071	2.276	1.2E-24	0.707	3.98E-44	intronic	*SUGP1*	.	.	.
rs58489806	19	19456917	2.197	4.5E-25	0.667	4.30E-43	intronic	*MAU2*	.	.	.
rs12484809	22	44325631	1.758	5.2E-19	0.507	1.06E-42	intronic	*PNPLA3*	.	.	.
rs12485100	22	44325516	1.758	5.2E-19	0.507	1.06E-42	intronic	*PNPLA3*	.	.	.
rs12484801	22	44325565	1.758	5.3E-19	0.507	1.11E-42	intronic	*PNPLA3*	.	.	.
rs9625962	22	44326272	1.755	6.2E-19	0.504	2.25E-42	intronic	*PNPLA3*	.	.	.
rs12483959	22	44325996	1.755	6.2E-19	0.504	2.25E-42	intronic	*PNPLA3*	.	.	.
rs11090617	22	44326700	1.755	6.1E-19	0.504	2.43E-42	intronic	*PNPLA3*	.	.	.
rs72999033	19	19366632	2.298	5.1E-22	0.760	3.14E-42	UTR3	*HAPLN4*	NM_023002:c.*1994G>A	.	.
rs36038527	22	44332888	1.742	4.7E-19	0.493	3.42E-42	intronic	*PNPLA3*	.	.	.
rs73176497	22	44336957	1.737	7.4E-19	0.493	3.44E-42	intronic	*PNPLA3*	.	.	.
rs2281135	22	44332570	1.736	8.0E-19	0.493	3.45E-42	intronic	*PNPLA3*	.	.	.
rs4823179	22	44341193	1.739	7.0E-19	0.493	3.75E-42	intronic	*PNPLA3*	.	.	.
rs34879941	22	44332878	1.735	8.7E-19	0.493	3.83E-42	intronic	*PNPLA3*	.	.	.
rs16991175	22	44335331	1.735	8.8E-19	0.493	3.96E-42	intronic	*PNPLA3*	.	.	.
rs34352134	22	44335416	1.735	8.8E-19	0.493	3.96E-42	intronic	*PNPLA3*	.	.	.
rs34376930	22	44335453	1.735	8.8E-19	0.493	3.96E-42	intronic	*PNPLA3*	.	.	.
rs35621602	22	44335406	1.735	8.8E-19	0.493	3.96E-42	intronic	*PNPLA3*	.	.	.
rs4823177	22	44334486	1.735	8.6E-19	0.493	3.99E-42	intronic	*PNPLA3*	.	.	.
rs4823178	22	44334529	1.735	8.6E-19	0.493	3.99E-42	intronic	*PNPLA3*	.	.	.
rs1010022	22	44336310	1.734	9.3E-19	0.493	4.00E-42	intronic	*PNPLA3*	.	.	.
rs1010023	22	44336098	1.734	9.3E-19	0.493	4.00E-42	intronic	*PNPLA3*	.	.	.
rs8142145	22	44336496	1.734	9.3E-19	0.493	4.00E-42	intronic	*PNPLA3*	.	.	.
rs2073081	22	44335744	1.735	9.0E-19	0.493	4.07E-42	intronic	*PNPLA3*	.	.	.
rs4823176	22	44334476	1.735	8.7E-19	0.493	4.08E-42	intronic	*PNPLA3*	.	.	.
rs2072905	22	44333479	1.735	8.7E-19	0.493	4.12E-42	intronic	*PNPLA3*	.	.	.
rs2072906	22	44333172	1.735	8.5E-19	0.493	4.12E-42	intronic	*PNPLA3*	.	.	.
rs2401512	22	44333945	1.735	8.7E-19	0.493	4.12E-42	intronic	*PNPLA3*	.	.	.
rs2896019	22	44333694	1.735	8.7E-19	0.493	4.12E-42	intronic	*PNPLA3*	.	.	.
rs2896020	22	44333968	1.735	8.7E-19	0.493	4.12E-42	intronic	*PNPLA3*	.	.	.
rs56255430	19	19477877	2.150	1.6E-22	0.671	4.25E-42	intergenic	*MAU2;GATAD2A*	dist=8314;dist=18775	.	.
rs13056555	22	44339526	1.737	8.4E-19	0.492	5.76E-42	intronic	*PNPLA3*	.	.	.
rs36069781	22	44340086	1.737	8.4E-19	0.492	5.76E-42	intronic	*PNPLA3*	.	.	.
rs2294916	22	44340922	1.736	8.7E-19	0.492	6.07E-42	intronic	*PNPLA3*	.	.	.
rs4823180	22	44341298	1.736	8.7E-19	0.492	6.07E-42	intronic	*PNPLA3*	.	.	.
rs4823181	22	44341606	1.736	8.5E-19	0.492	6.07E-42	intronic	*PNPLA3*	.	.	.
rs1883349	22	44331943	1.735	1.1E-18	0.493	6.64E-42	intronic	*PNPLA3*	.	.	.
rs926633	22	44337533	1.731	1.3E-18	0.491	7.49E-42	intronic	*PNPLA3*	.	.	.
rs1997693	22	44331513	1.740	8.3E-19	0.493	8.87E-42	intronic	*PNPLA3*	.	.	.
rs17217098	19	19702384	2.222	1.2E-21	0.727	1.24E-41	intronic	*PBX4*	.	.	.
rs1810508	22	44343151	1.728	1.9E-18	0.489	1.60E-41	UTR3	*PNPLA3*	NM_025225:c.*889A>G	.	.
rs2008451	22	44342969	1.728	1.9E-18	0.489	1.60E-41	UTR3	*PNPLA3*	NM_025225:c.*707T>C	.	.
rs2281293	22	44334842	1.730	1.4E-18	0.489	1.83E-41	intronic	*PNPLA3*	.	.	.
rs2076207	22	44333370	1.730	1.4E-18	0.489	1.90E-41	intronic	*PNPLA3*	.	.	.
rs2072907	22	44332653	1.730	1.4E-18	0.488	1.96E-41	intronic	*PNPLA3*	.	.	.
rs2281138	22	44332477	1.730	1.4E-18	0.488	2.03E-41	intronic	*PNPLA3*	.	.	.
rs13054885	22	44345771	1.727	2.4E-18	0.486	2.07E-41	intergenic	*PNPLA3;SAMM50*	dist=2309;dist=5551	.	.
rs13056638	22	44331778	1.734	1.3E-18	0.490	2.08E-41	intronic	*PNPLA3*	.	.	.
rs2281137	22	44332493	1.730	1.4E-18	0.488	2.10E-41	intronic	*PNPLA3*	.	.	.
rs1883348	22	44331815	1.732	1.5E-18	0.490	2.17E-41	intronic	*PNPLA3*	.	.	.
rs13055874	22	44341672	1.728	2.0E-18	0.488	2.78E-41	intronic	*PNPLA3*	.	.	.
rs13055900	22	44341666	1.728	2.0E-18	0.488	2.78E-41	intronic	*PNPLA3*	.	.	.
rs2294433	22	44329275	1.752	1.2E-18	0.496	4.96E-41	intronic	*PNPLA3*	.	.	.
rs1977080	22	44330031	1.746	1.5E-18	0.494	6.74E-41	intronic	*PNPLA3*	.	.	.
rs4823173	22	44328730	1.743	2.2E-18	0.495	8.18E-41	intronic	*PNPLA3*	.	.	.
rs2076211	22	44329078	1.743	2.2E-18	0.495	8.24E-41	intronic	*PNPLA3*	.	.	.
rs16991158	22	44327179	1.742	2.4E-18	0.494	9.58E-41	intronic	*PNPLA3*	.	.	.
rs36055245	22	44327192	1.741	2.5E-18	0.494	9.97E-41	intronic	*PNPLA3*	.	.	.
rs73004967	19	19717056	2.181	1.5E-20	0.718	1.03E-40	intronic	*PBX4*	.	.	.
rs1977081	22	44330128	1.742	2.5E-18	0.491	2.30E-40	intronic	*PNPLA3*	.	.	.
19:19699398_GA_G	19	19699398	2.214	3.7E-21	0.719	3.12E-40	.	.	.	.	.
rs12484700	22	44327273	1.741	2.6E-18	0.490	3.76E-40	intronic	*PNPLA3*	.	.	.
rs2092501	22	44347251	1.750	2.4E-18	0.491	1.02E-39	intergenic	*PNPLA3;SAMM50*	dist=3789;dist=4071	.	.
rs34912062	22	44348446	1.743	3.7E-18	0.489	1.62E-39	intergenic	*PNPLA3;SAMM50*	dist=4984;dist=2876	.	.
rs1474745	22	44349236	1.745	2.7E-18	0.484	4.44E-39	intergenic	*PNPLA3;SAMM50*	dist=5774;dist=2086	.	.
rs56373884	22	44356468	1.747	2.0E-18	0.486	6.50E-39	intronic	*SAMM50*	.	.	.
rs3794991	19	19610596	2.055	6.0E-20	0.642	1.07E-38	intronic	*GATAD2A*	.	.	.
rs73002956	19	19578743	2.057	6.1E-20	0.642	2.25E-38	intronic	*GATAD2A*	.	.	.
rs2294921	22	44361842	1.739	2.6E-18	0.480	2.42E-38	intronic	*SAMM50*	.	.	.
rs12484795	22	44343626	1.685	5.4E-17	0.464	3.28E-38	downstream	*PNPLA3*	dist=164	.	.
rs3761472	22	44368122	1.734	3.9E-18	0.478	3.55E-38	exonic	*SAMM50*	.	nonsynonymous SNV	SAMM50:NM_015380:exon5:c.A329G:p.D110G
rs17216525	19	19662220	2.039	4.0E-19	0.648	4.26E-38	intergenic	*CILP2;PBX4*	dist=4752;dist=10302	.	.
rs16996148	19	19658472	2.019	9.8E-19	0.644	5.35E-38	intergenic	*CILP2;PBX4*	dist=1004;dist=14050	.	.
rs143988316	19	19667254	2.028	5.8E-19	0.643	7.55E-38	intergenic	*CILP2;PBX4*	dist=9786;dist=5268	.	.
rs17216588	19	19664077	2.020	9.7E-19	0.638	3.70E-37	intergenic	*CILP2;PBX4*	dist=6609;dist=8445	.	.
rs150824230	19	19670610	2.042	2.5E-19	0.633	9.90E-37	intergenic	*CILP2;PBX4*	dist=13142;dist=1912	.	.
rs73004926	19	19671266	2.041	3.7E-19	0.635	1.04E-36	intergenic	*CILP2;PBX4*	dist=13798;dist=1256	.	.

Table continues on next page.

SNP info			Case-control GWAS		Quantitative GWAS		Annotation				
ID	CHROM	POS	OR	P	BETA	P	Func.refGene	Gene.refGene	GeneDetail.refGene	ExonicFunc.refGene	AAChange.refGene
rs73004933	19	19675696	2.040	3.8E-19	0.634	1.09E-36	intronic	PBX4	.	.	.
rs141756246	19	19685470	2.038	4.9E-19	0.631	3.10E-36	intronic	PBX4	.	.	.
rs73004962	19	19713069	2.005	1.3E-18	0.619	8.46E-36	intronic	PBX4	.	.	.
rs2228603	19	19329924	2.119	1.0E-20	0.645	9.10E-36	exonic	NCAN	.	nonsynonymous SNV	NCAN:NM_004386:exon3:c.C274T:p.P92S
rs12610185	19	19721722	1.994	2.7E-18	0.616	1.54E-35	intronic	PBX4	.	.	.
rs12610191	19	19721976	1.994	2.7E-18	0.616	1.54E-35	intronic	PBX4	.	.	.
rs73004951	19	19695228	2.003	1.8E-18	0.618	1.71E-35	intronic	PBX4	.	.	.
rs58847337	19	19726022	1.992	3.0E-18	0.614	2.10E-35	intronic	PBX4	.	.	.
rs10500212	19	19723215	1.991	3.1E-18	0.614	2.15E-35	intronic	PBX4	.	.	.
rs16996185	19	19720788	1.991	3.1E-18	0.614	2.15E-35	intronic	PBX4	.	.	.
rs57504626	19	19720399	1.991	3.1E-18	0.614	2.15E-35	intronic	PBX4	.	.	.
rs73004966	19	19716558	1.989	3.5E-18	0.614	2.15E-35	intronic	PBX4	.	.	.
rs12608729	19	19700552	2.001	1.5E-18	0.614	2.27E-35	intronic	PBX4	.	.	.
rs73004959	19	19711139	1.989	4.5E-18	0.616	2.41E-35	intronic	PBX4	.	.	.
rs73004975	19	19727152	1.986	4.3E-18	0.611	4.19E-35	intronic	PBX4	.	.	.
rs4823109	22	44381482	1.713	4.5E-16	0.461	2.02E-32	intronic	SAMM50	.	.	.
rs2235777	22	44378809	1.709	5.2E-16	0.460	2.14E-32	intronic	SAMM50	.	.	.
rs4823183	22	44378672	1.709	5.2E-16	0.460	2.14E-32	intronic	SAMM50	.	.	.
rs4823108	22	44381340	1.711	5.0E-16	0.461	2.30E-32	intronic	SAMM50	.	.	.
rs61473277	22	44371406	1.706	6.6E-16	0.459	2.38E-32	intronic	SAMM50	.	.	.
rs2235776	22	44377999	1.709	5.2E-16	0.459	2.75E-32	intronic	SAMM50	.	.	.
rs71313378	22	44380170	1.708	5.6E-16	0.460	2.79E-32	intronic	SAMM50	.	.	.
rs12167845	22	44380767	1.707	6.4E-16	0.459	2.88E-32	intronic	SAMM50	.	.	.
rs2294923	22	44379740	1.706	6.9E-16	0.458	3.29E-32	intronic	SAMM50	.	.	.
rs9626079	22	44380009	1.706	6.9E-16	0.458	3.29E-32	intronic	SAMM50	.	.	.
rs2304128	19	19746151	2.061	1.2E-18	0.597	2.66E-31	intronic	GMIP	.	.	.
rs57962361	19	19425025	1.793	4.5E-16	0.462	1.05E-26	intronic	SUGP1	.	.	.
rs111234557	19	19436229	1.788	6.5E-16	0.460	1.65E-26	intronic	MAU2	.	.	.
rs11668104	19	19426181	1.782	9.1E-16	0.457	2.80E-26	intronic	SUGP1	.	.	.
rs11672355	19	19462702	1.778	1.1E-15	0.457	3.05E-26	intronic	MAU2	.	.	.
rs11411903	19	19440064	1.777	1.3E-15	0.456	3.78E-26	intronic	MAU2	.	.	.
19:19756073_AGCC_A	19	19756073	1.912	1.1E-13	0.569	3.13E-25	.	.	.	.	.
rs12979148	19	19406869	1.662	1.5E-13	0.422	4.22E-25	intronic	SUGP1	.	.	.
rs2240117	19	19418916	1.640	8.5E-13	0.418	1.32E-24	intronic	SUGP1	.	.	.
19:19450254_CA_C	19	19450254	1.738	1.1E-14	0.429	1.29E-23	.	.	.	.	.
rs56408111	19	19793545	1.819	1.6E-13	0.499	1.76E-23	UTR3	ZNF101	NM_001300949:c.*2436T>C;NM_033204:c.*2436T>C	.	.
rs58434384	19	19786099	1.783	3.1E-13	0.481	3.98E-23	intronic	ZNF101	.	.	.
rs2304130	19	19789528	1.779	3.5E-13	0.479	5.08E-23	intronic	ZNF101	.	.	.
rs12052117	19	19485105	1.667	6.9E-13	0.409	1.80E-22	intergenic	MAU2;GATAD2A	dist=15542;dist=11547	.	.
rs73002960	19	19582992	1.656	1.4E-12	0.407	3.43E-22	intronic	GATAD2A	.	.	.
rs3761077	19	19325963	1.723	9.7E-14	0.423	3.66E-22	intronic	NCAN	.	.	.
rs28720066	19	19572220	1.654	1.6E-12	0.406	4.24E-22	intronic	GATAD2A	.	.	.
rs2285626	19	19467545	1.681	1.2E-13	0.399	4.91E-22	UTR3	MAU2	NM_015329:c.*954C>T	.	.
rs59148799	19	19484008	1.651	2.0E-12	0.404	5.03E-22	intergenic	MAU2;GATAD2A	dist=14445;dist=12644	.	.
rs56273306	19	19621004	1.647	2.3E-12	0.404	5.25E-22	intergenic	GATAD2A;TSSK6	dist=1263;dist=4024	.	.
rs56241616	19	19506092	1.649	2.1E-12	0.404	5.57E-22	intronic	GATAD2A	.	.	.
rs8182472	19	19539891	1.648	2.2E-12	0.404	5.58E-22	intronic	GATAD2A	.	.	.
rs10408875	19	19503573	1.649	2.1E-12	0.404	5.70E-22	intronic	GATAD2A	.	.	.
rs10408596	19	19512657	1.649	2.2E-12	0.404	5.71E-22	intronic	GATAD2A	.	.	.
rs10415849	19	19505087	1.647	2.3E-12	0.403	6.32E-22	intronic	GATAD2A	.	.	.
rs113460678	19	19513580	1.642	3.1E-12	0.401	1.00E-21	intronic	GATAD2A	.	.	.
rs34324111	19	19513568	1.642	3.1E-12	0.401	1.00E-21	intronic	GATAD2A	.	.	.
rs35629458	19	19513572	1.642	3.1E-12	0.401	1.00E-21	intronic	GATAD2A	.	.	.
rs34755166	19	19665581	1.575	9.7E-11	0.394	1.22E-21	intergenic	CILP2;PBX4	dist=8113;dist=6941	.	.
rs17216693	19	19666574	1.572	9.8E-11	0.392	1.43E-21	intergenic	CILP2;PBX4	dist=9106;dist=5948	.	.
rs2281298	22	44391234	1.463	9.7E-10	0.336	1.45E-21	intronic	SAMM50	.	.	.
rs2143571	22	44391686	1.460	1.3E-09	0.336	1.48E-21	intronic	SAMM50	.	.	.
rs56397647	19	19642795	1.619	2.5E-11	0.403	2.65E-21	intronic	YJEFN3	.	.	.
rs2401514	22	44394019	1.457	1.3E-09	0.331	4.39E-21	intergenic	SAMM50;PARVB	dist=1610;dist=1072	.	.
rs2073079	22	44385594	1.457	1.3E-09	0.330	4.98E-21	intronic	SAMM50	.	.	.
rs2073080	22	44394402	1.455	1.5E-09	0.330	5.53E-21	upstream	PARVB	dist=689	.	.
rs3827385	22	44388817	1.456	1.3E-09	0.328	6.47E-21	intronic	SAMM50	.	.	.
rs2238675	19	19336608	1.666	3.3E-13	0.379	2.44E-20	intronic	NCAN	.	.	.
rs11668386	19	19531910	1.589	4.4E-11	0.376	7.99E-20	intronic	GATAD2A	.	.	.
rs35431065	19	19393677	1.547	3.5E-11	0.340	8.93E-20	intronic	SUGP1	.	.	.
rs10424702	19	19508013	1.582	6.5E-11	0.371	1.57E-19	intronic	GATAD2A	.	.	.
rs79954596	19	19548643	1.577	9.0E-11	0.370	1.94E-19	intronic	GATAD2A	.	.	.
rs188552254	19	19517169	1.578	8.3E-11	0.370	1.98E-19	intronic	GATAD2A	.	.	.
19:19668338_GC_G	19	19668338	1.556	3.3E-10	0.372	2.03E-19	.	.	.	.	.
rs113365218	19	19621197	1.607	5.6E-11	0.379	2.09E-19	intergenic	GATAD2A;TSSK6	dist=1456;dist=3831	.	.
rs67450864	22	44376335	1.405	3.4E-10	0.260	3.85E-19	intronic	SAMM50	.	.	.
rs4823182	22	44377442	1.406	3.0E-10	0.259	4.08E-19	intronic	SAMM50	.	.	.
rs57009615	19	19613622	1.567	9.2E-11	0.356	1.27E-18	intronic	GATAD2A	.	.	.
rs12165526	22	44361713	1.736	1.1E-12	0.410	1.44E-18	intronic	SAMM50	.	.	.
rs10656207	22	44387932	1.381	1.1E-09	0.245	2.11E-18	intronic	SAMM50	.	.	.
22:44335670_TGG_T	22	44335670	1.372	2.3E-09	0.239	2.93E-18	.	.	.	.	.
rs1007863	22	44395451	1.370	2.5E-09	0.243	3.54E-18	exonic	PARVB	.	nonsynonymous SNV	PARVB:NM_001003828:exon2:c.T109C:p.W37R
rs6006468	22	44383432	1.375	1.8E-09	0.242	5.53E-18	intronic	SAMM50	.	.	.
rs6006602	22	44383400	1.375	1.8E-09	0.242	5.53E-18	intronic	SAMM50	.	.	.
rs2294927	22	44382684	1.378	1.3E-09	0.243	5.80E-18	intronic	SAMM50	.	.	.
rs2235778	22	44389514	1.372	2.2E-09	0.242	6.03E-18	intronic	SAMM50	.	.	.
rs6006473	22	44393075	1.369	2.9E-09	0.241	6.19E-18	downstream	SAMM50	dist=666	.	.
rs6006469	22	44383617	1.373	2.1E-09	0.241	7.09E-18	intronic	SAMM50	.	.	.
rs2281292	22	44395389	1.365	3.9E-09	0.240	7.22E-18	intronic	PARVB	.	.	.
rs3788604	22	44388417	1.370	2.5E-09	0.240	8.25E-18	intronic	SAMM50	.	.	.
rs1986095	22	44387108	1.369	2.6E-09	0.240	8.64E-18	intronic	SAMM50	.	.	.
rs16991236	22	44358997	.	.	0.446	1.50E-17	intronic	SAMM50	.	.	.
rs1883350	22	44328043	.	.	0.254	6.68E-17	intronic	PNPLA3	.	.	.
rs73008942	19	19155672	1.820	1.9E-09	0.484	3.88E-15	intronic	ARMC6	.	.	.
rs73006914	19	19110422	1.801	5.0E-09	0.487	4.64E-15	intronic	SUGP2	.	.	.

Table continues on next page.

SNP info			Case-control GWAS		Quantitative GWAS		Annotation				
ID	CHROM	POS	OR	P	BETA	P	Func.refGene	Gene.refGene	GeneDetail.refGene	ExonicFunc.refGene	AAChange.refGene
rs80341032	19	19207229	1.805	9.6E-10	0.461	1.08E-14	intronic	*SLC25A42*	.	.	.
rs58833986	19	19434042	1.437	1.2E-08	0.276	2.36E-14	intronic	*MAU2*	.	.	.
rs3810444	19	19103986	1.748	6.5E-09	0.449	2.48E-14	UTR3	*SUGP2*	NM_001321699:c.*564A>T;NM_001352071:c.*564A>T;NM_001017392:c.*564A>T	.	.
rs8105094	19	19374061	1.467	2.7E-09	0.281	2.54E-14	upstream	*HAPLN4*	dist=448	.	.
rs8105984	19	19374068	1.467	2.7E-09	0.281	2.54E-14	upstream	*HAPLN4*	dist=455	.	.
rs9626056	22	44327075	1.790	3.4E-10	0.434	4.23E-14	intronic	*PNPLA3*	.	.	.
rs9306471	22	44328075	1.795	2.8E-10	0.433	5.63E-14	intronic	*PNPLA3*	.	.	.
rs2023883	19	19405480	1.421	3.7E-08	0.269	7.88E-14	intronic	*SUGP1*	.	.	.
rs11666553	19	19407171	1.420	3.9E-08	0.269	8.31E-14	intronic	*SUGP1*	.	.	.
19:19422152_CA_C	19	19422152	1.420	4.3E-08	0.270	8.32E-14	.	.	.	.	.
rs12459676	19	19425141	1.421	3.5E-08	0.268	8.95E-14	intronic	*SUGP1*	.	.	.
rs12981405	19	19651577	.	.	0.276	9.06E-14	intronic	*CILP2*	.	.	.
rs2074301	19	19381715	1.433	1.7E-08	0.269	9.06E-14	intronic	*TM6SF2*	.	.	.
rs2285628	19	19467996	1.427	2.4E-08	0.268	1.06E-13	UTR3	*MAU2*	NM_015329:c.*1405T>A	.	.
rs775175628	19	19560756	.	.	0.272	1.09E-13	.	.	.	.	.
rs2074300	19	19380996	1.432	1.8E-08	0.268	1.13E-13	exonic	*TM6SF2*	.	synonymous SNV	TM6SF2:NM_001001524:exon4:c.C387A:p.G129G
rs10419672	19	19471241	1.423	3.0E-08	0.266	1.32E-13	intergenic	*MAU2;GATAD2A*	dist=1678;dist=25411	.	.
rs111393709	22	44329719	1.791	3.6E-10	0.426	1.32E-13	intronic	*PNPLA3*	.	.	.
rs10402661	19	19432959	1.416	4.7E-08	0.265	1.38E-13	intronic	*MAU2*	.	.	.
rs12983137	19	19419810	1.416	4.7E-08	0.265	1.38E-13	intronic	*SUGP1*	.	.	.
rs1859287	19	19422187	1.416	4.7E-08	0.265	1.38E-13	intronic	*SUGP1*	.	.	.
rs7259434	19	19428805	1.416	4.7E-08	0.265	1.38E-13	intronic	*SUGP1*	.	.	.
rs12976025	19	19429220	1.416	4.5E-08	0.265	1.39E-13	intronic	*SUGP1*	.	.	.
rs4808194	19	19435680	1.416	4.5E-08	0.265	1.39E-13	intronic	*MAU2*	.	.	.
rs10403731	19	19469296	1.417	4.5E-08	0.266	1.44E-13	UTR3	*MAU2*	NM_015329:c.*2705G>A	.	.
rs11085261	19	19462606	.	.	0.265	1.53E-13	intronic	*MAU2*	.	.	.
rs563530889	19	19374546	1.427	2.6E-08	0.267	1.54E-13	upstream;downstream	*HAPLN4;TM6SF2*	dist=933;dist=630	.	.
rs11085259	19	19445856	.	.	0.265	1.61E-13	intronic	*MAU2*	.	.	.
rs8108647	19	19427623	.	.	0.264	1.62E-13	intronic	*SUGP1*	.	.	.
rs2301668	19	19452249	1.415	5.0E-08	0.265	1.62E-13	intronic	*MAU2*	.	.	.
rs757000	19	19448301	1.415	5.0E-08	0.265	1.62E-13	intronic	*MAU2*	.	.	.
rs757001	19	19448808	1.415	5.0E-08	0.265	1.62E-13	intronic	*MAU2*	.	.	.
rs12982276	19	19459800	.	.	0.264	1.75E-13	intronic	*MAU2*	.	.	.
rs756264087	19	19423083	.	.	0.264	1.83E-13	.	.	.	.	.
rs10421505	19	19459554	.	.	0.264	1.86E-13	intronic	*MAU2*	.	.	.
rs7258508	19	19475469	.	.	0.263	2.06E-13	intergenic	*MAU2;GATAD2A*	dist=5906;dist=21183	.	.
rs968525	19	19459215	.	.	0.263	2.24E-13	intronic	*MAU2*	.	.	.
rs55768287	22	44324558	1.765	2.2E-09	0.430	2.27E-13	intronic	*PNPLA3*	.	.	.
rs2294917	22	44341986	0.724	3.7E-08	-0.211	2.58E-13	intronic	*PNPLA3*	.	.	.
19:19431420_GT_G	19	19431420	.	.	0.285	2.66E-13	.	.	.	.	.
rs9304960	19	19465529	.	.	0.261	3.60E-13	intronic	*MAU2*	.	.	.
rs2301671	19	19466269	.	.	0.260	3.79E-13	intronic	*MAU2*	.	.	.
19:19393106_CAAGA_C	19	19393106	1.424	3.5E-08	0.263	4.01E-13	.	.	.	.	.
rs4808196	19	19476520	.	.	0.259	5.42E-13	intergenic	*MAU2;GATAD2A*	dist=6957;dist=20132	.	.
rs12166587	22	44383070	1.672	6.0E-09	0.383	6.94E-13	intronic	*SAMM50*	.	.	.
rs9625970	22	44383502	1.671	6.0E-09	0.383	7.01E-13	intronic	*SAMM50*	.	.	.
rs112902984	22	44385583	1.670	6.3E-09	0.383	7.31E-13	intronic	*SAMM50*	.	.	.
rs117472787	22	44387298	1.670	6.3E-09	0.383	7.31E-13	intronic	*SAMM50*	.	.	.
rs738491	22	44354111	.	.	0.218	8.22E-13	intronic	*SAMM50*	.	.	.
rs10418051	19	19397789	.	.	0.252	8.93E-13	intronic	*SUGP1*	.	.	.
rs11404084	19	19579241	.	.	0.255	9.99E-13	intronic	*GATAD2A*	.	.	.
rs8141950	22	44393476	1.662	1.0E-08	0.381	1.16E-12	intergenic	*SAMM50;PARVB*	dist=1067;dist=1615	.	.
rs10404728	19	19595014	.	.	0.253	1.37E-12	intronic	*GATAD2A*	.	.	.
rs1465695	19	19588546	.	.	0.253	1.37E-12	intronic	*GATAD2A*	.	.	.
rs751858	19	19602821	.	.	0.253	1.38E-12	intronic	*GATAD2A*	.	.	.
rs17288409	19	19504167	.	.	0.253	1.39E-12	intronic	*GATAD2A*	.	.	.
rs10401193	19	19591066	.	.	0.253	1.39E-12	intronic	*GATAD2A*	.	.	.
rs9625965	22	44338049	1.691	4.5E-09	0.388	1.41E-12	intronic	*PNPLA3*	.	.	.
rs754255	19	19578890	.	.	0.253	1.48E-12	intronic	*GATAD2A*	.	.	.
rs60003758	19	19557353	.	.	0.253	1.50E-12	intronic	*GATAD2A*	.	.	.
rs9625966	22	44338105	1.691	4.4E-09	0.387	1.50E-12	intronic	*PNPLA3*	.	.	.
rs9625964	22	44337610	1.691	4.4E-09	0.387	1.52E-12	intronic	*PNPLA3*	.	.	.
rs12972397	19	19562349	.	.	0.252	1.58E-12	intronic	*GATAD2A*	.	.	.
rs9626057	22	44339791	1.687	5.2E-09	0.385	1.83E-12	intronic	*PNPLA3*	.	.	.
rs60321073	19	19499598	.	.	0.252	1.98E-12	intronic	*GATAD2A*	.	.	.
rs2099333	19	19605963	.	.	0.252	2.04E-12	intronic	*GATAD2A*	.	.	.
rs56219234	22	44357894	.	.	0.215	2.10E-12	intronic	*SAMM50*	.	.	.
rs61061000	19	19283268	.	.	0.305	2.17E-12	intronic	*BORCS8-MEF2B*	.	.	.
rs12977524	19	19568244	.	.	0.251	2.25E-12	intronic	*GATAD2A*	.	.	.
rs4808199	19	19545099	.	.	0.251	2.27E-12	intronic	*GATAD2A*	.	.	.
rs117772800	22	44339055	1.685	5.7E-09	0.383	2.29E-12	intronic	*PNPLA3*	.	.	.
rs7250658	19	19571100	.	.	0.251	2.36E-12	intronic	*GATAD2A*	.	.	.
rs6511036	19	19582651	.	.	0.251	2.45E-12	intronic	*GATAD2A*	.	.	.
rs7252888	19	19628037	.	.	0.250	2.48E-12	intronic	*NDUFA13*	.	.	.
rs2163805	19	19575945	.	.	0.251	2.50E-12	intronic	*GATAD2A*	.	.	.
rs4808960	19	19574277	.	.	0.251	2.54E-12	intronic	*GATAD2A*	.	.	.
rs2163804	19	19575965	.	.	0.251	2.56E-12	intronic	*GATAD2A*	.	.	.
19:19510831_AT_A	19	19510831	.	.	0.250	3.16E-12	.	.	.	.	.
rs738494	22	44370439	1.654	1.9E-08	0.375	4.62E-12	intronic	*SAMM50*	.	.	.
rs9626061	22	44344872	1.645	4.0E-08	0.378	4.95E-12	intergenic	*PNPLA3;SAMM50*	dist=1410;dist=6450	.	.
rs9626071	22	44359938	1.652	2.0E-08	0.373	5.57E-12	intronic	*SAMM50*	.	.	.
rs8141994	22	44397144	1.655	1.7E-08	0.371	5.59E-12	intronic	*PARVB*	.	.	.
rs12168138	22	44359651	1.649	2.2E-08	0.373	5.80E-12	intronic	*SAMM50*	.	.	.
rs28754570	22	44358819	1.650	2.2E-08	0.373	5.83E-12	intronic	*SAMM50*	.	.	.
rs12170274	22	44359729	1.650	2.2E-08	0.373	5.83E-12	intronic	*SAMM50*	.	.	.
rs28421169	22	44356562	1.650	2.1E-08	0.372	6.10E-12	intronic	*SAMM50*	.	.	.
rs9626058	22	44343352	1.645	3.3E-08	0.375	6.43E-12	UTR3	*PNPLA3*	NM_025225:c.*1090A>G	.	.
rs6006599	22	44382004	.	.	0.209	7.42E-12	intronic	*SAMM50*	.	.	.
rs9626076	22	44368584	1.639	3.9E-08	0.371	8.15E-12	intronic	*SAMM50*	.	.	.
rs12168183	22	44366135	1.637	4.2E-08	0.371	8.25E-12	intronic	*SAMM50*	.	.	.

Table continues on next page.

SNP info			Case-control GWAS		Quantitative GWAS		Annotation				
ID	CHROM	POS	OR	P	BETA	P	Func.refGene	Gene.refGene	GeneDetail.refGene	ExonicFunc.refGene	
rs9626075	22	44366874	1.639	3.9E-08	0.371	8.52E-12	intronic	*SAMM50*	.	.	
rs28733632	22	44353447	.	.	0.371	8.59E-12	intronic	*SAMM50*	.	.	
rs117369516	22	44348284	.	.	0.372	8.68E-12	intergenic	*PNPLA3;SAMM50*	dist=4822;dist=3038	.	
rs9626064	22	44354598	.	.	0.371	8.93E-12	intronic	*SAMM50*	.	.	
rs9626074	22	44363736	1.638	4.1E-08	0.371	9.01E-12	intronic	*SAMM50*	.	.	
rs117130990	22	44371030	1.638	4.1E-08	0.370	9.15E-12	intronic	*SAMM50*	.	.	
rs12330016	22	44369927	1.638	4.1E-08	0.370	9.15E-12	intronic	*SAMM50*	.	.	
rs9626073	22	44362178	1.638	4.1E-08	0.370	9.17E-12	intronic	*SAMM50*	.	.	
rs73434655	22	44370955	1.637	4.2E-08	0.370	9.23E-12	intronic	*SAMM50*	.	.	
rs12167852	22	44344011	.	.	0.372	9.59E-12	downstream	*PNPLA3*	dist=549	.	
rs41278873	22	44342691	1.666	7.3E-09	0.364	1.03E-11	UTR3	*PNPLA3*	NM_025225:c.*429T>C	.	
rs9626078	22	44373947	1.633	5.0E-08	0.369	1.04E-11	intronic	*SAMM50*	.	.	
rs28478453	19	19531175	.	.	0.241	1.09E-11	intronic	*GATAD2A*	.	.	
rs9626065	22	44357928	1.633	4.9E-08	0.369	1.10E-11	intronic	*SAMM50*	.	.	
rs9626066	22	44357940	1.634	4.8E-08	0.368	1.12E-11	intronic	*SAMM50*	.	.	
rs9626067	22	44358030	1.634	4.8E-08	0.368	1.12E-11	intronic	*SAMM50*	.	.	
rs75439392	22	44377221	.	.	0.368	1.16E-11	intronic	*SAMM50*	.	.	
rs9626068	22	44358360	1.636	4.5E-08	0.368	1.16E-11	intronic	*SAMM50*	.	.	
rs11912828	22	44348116	.	.	-0.211	1.19E-11	intergenic	*PNPLA3;SAMM50*	dist=4654;dist=3206	.	
rs2294919	22	44342325	.	.	-0.213	1.25E-11	UTR3	*PNPLA3*	NM_025225:c.*63C>T	.	
rs565756573	19	19461416	.	.	0.258	1.29E-11	intronic	*MAU2*	.	.	
rs113954889	19	19294392	.	.	0.300	1.33E-11	intronic	*BORCS8;BORCS8-MEF2B*	.	.	
rs10402308	19	19657500	.	.	0.244	1.36E-11	downstream	*CILP2*	dist=32	.	
rs12170782	22	44347504	.	.	0.368	1.69E-11	intergenic	*PNPLA3;SAMM50*	dist=4042;dist=3818	.	
rs11669516	19	19532682	.	.	0.237	1.95E-11	intronic	*GATAD2A*	.	.	
rs139052	22	44327012	.	.	-0.214	2.10E-11	intronic	*PNPLA3*	.	.	
rs34647936	19	19548239	.	.	0.236	2.33E-11	intronic	*GATAD2A*	.	.	
rs12983940	19	19516431	.	.	0.237	2.35E-11	intronic	*GATAD2A*	.	.	
rs12973258	19	19488718	.	.	0.235	2.89E-11	intergenic	*MAU2;GATAD2A*	dist=19155;dist=7934	.	
rs4808950	19	19518889	.	.	0.234	3.69E-11	intronic	*GATAD2A*	.	.	
rs8100140	19	19314526	.	.	0.186	6.10E-11	upstream	*NR2C2AP*	dist=303	.	
22:44381944_ATGGAGTCTTGCTC_A	22	44381944	.	.	0.200	1.05E-10	.	.	.	.	
rs1474744	22	44349215	.	.	-0.174	1.30E-10	intergenic	*PNPLA3;SAMM50*	dist=5753;dist=2107	.	
rs111901094	19	19513570	.	.	0.234	1.36E-10	intronic	*GATAD2A*	.	.	
rs2965185	19	19525792	.	.	0.182	8.65E-10	intronic	*GATAD2A*	.	.	
rs12484530	22	44409993	.	.	0.292	1.61E-09	intronic	*PARVB*	.	.	
rs60204587	19	54671421	.	.	0.167	2.32E-09	intronic	*TMC4*	.	.	
rs6006594	22	44365232	.	.	-0.163	3.02E-09	intronic	*SAMM50*	.	.	
rs28451834	19	19260760	.	.	0.245	3.66E-09	intronic	*BORCS8-MEF2B;MEF2B*	.	.	
rs2073086	22	44372331	.	.	0.183	3.76E-09	intronic	*SAMM50*	.	.	
rs12609436	19	19743098	.	.	0.161	3.93E-09	intronic	*GMIP*	.	.	
rs4806498	19	54674742	.	.	0.163	4.04E-09	intronic	*TMC4*	.	.	
rs1474746	22	44350417	.	.	-0.161	4.41E-09	upstream	*SAMM50*	dist=905	.	
rs873870	19	19738554	.	.	0.159	4.55E-09	intronic	*LPAR2*	.	.	
rs139051	22	44324676	.	.	0.168	4.56E-09	intronic	*PNPLA3*	.	.	
rs3083314	22	44343239	.	.	-0.160	6.53E-09	.	.	.	.	
rs2401513	22	44355569	.	.	-0.185	7.18E-09	intronic	*SAMM50*	.	.	
rs2872878	19	19480521	.	.	0.167	7.66E-09	intergenic	*MAU2;GATAD2A*	dist=10958;dist=16131	.	
rs2073084	22	44372069	.	.	0.179	8.48E-09	intronic	*SAMM50*	.	.	
rs28550680	22	44345926	.	.	-0.185	8.57E-09	intergenic	*PNPLA3;SAMM50*	dist=2464;dist=5396	.	
rs641738	19	54676763	.	.	0.159	9.27E-09	exonic	*TMC4*	.	nonsynonymous SNV	TMC4:NM_00114530[illegible]
rs11704562	22	44346128	.	.	-0.184	9.27E-09	intergenic	*PNPLA3;SAMM50*	dist=2666;dist=5194	.	
rs7289329	22	44346639	.	.	-0.184	9.32E-09	intergenic	*PNPLA3;SAMM50*	dist=3177;dist=4683	.	
rs2073085	22	44372190	.	.	0.178	9.63E-09	intronic	*SAMM50*	.	.	
rs11705218	22	44354865	.	.	-0.183	9.77E-09	intronic	*SAMM50*	.	.	

Table continues on next page.

SNP info			Case-control GWAS		Quantitative GWAS		Annotation				
ID	CHROM	POS	OR	P	BETA	P	Func.refGene	Gene.refGene	GeneDetail.refGene	ExonicFunc.refGene	AAChange.refGene
rs7254927	19	19404786	.	.	0.163	1.56E-08	intronic	*SUGP1*	.	.	.
rs4808938	19	19401218	.	.	0.163	1.56E-08	intronic	*SUGP1*	.	.	.
22:44345952_AAAAAAAT_A	22	44345952	.	.	-0.182	1.57E-08	.	.	.	.	.
rs7252981	19	19692579	.	.	0.164	1.59E-08	intronic	*PBX4*	.	.	.
rs8108705	19	19675367	.	.	0.164	1.60E-08	intronic	*PBX4*	.	.	.
19:19394249_GTT_G	19	19394249	.	.	0.163	1.67E-08	.	.	.	.	.
rs2294918	22	44342116	.	.	-0.156	1.68E-08	exonic	*PNPLA3*	.	synonymous SNV	PNPLA3:NM_025225:exon9:c.A1300A:p.K434K
rs35534408	19	19409021	.	.	0.163	1.71E-08	intronic	*SUGP1*	.	.	.
rs2301786	19	19413947	.	.	0.162	1.72E-08	intronic	*SUGP1*	.	.	.
rs2315022	19	19413381	.	.	0.162	1.72E-08	intronic	*SUGP1*	.	.	.
rs2074296	19	19373689	.	.	0.163	1.74E-08	upstream	*HAPLN4*	dist=76	.	.
rs9614294	22	44356566	.	.	-0.182	1.76E-08	intronic	*SAMM50*	.	.	.
rs2235772	22	44369329	.	.	0.175	1.76E-08	intronic	*SAMM50*	.	.	.
rs1010207	19	19416045	.	.	0.162	1.78E-08	intronic	*SUGP1*	.	.	.
rs2064361	22	44356349	.	.	-0.182	1.79E-08	intronic	*SAMM50*	.	.	.
rs10407283	19	19744358	.	.	0.153	1.85E-08	intronic	*GMIP*	.	.	.
rs2285859	19	19422485	.	.	0.162	1.90E-08	intronic	*SUGP1*	.	.	.
rs12977937	19	19429975	.	.	0.162	1.91E-08	intronic	*SUGP1*	.	.	.
rs4808942	19	19420581	.	.	0.162	1.91E-08	intronic	*SUGP1*	.	.	.
rs10426780	19	19375883	.	.	0.163	1.93E-08	intronic	*TM6SF2*	.	.	.
rs8736	19	54677189	.	.	0.156	1.96E-08	UTR3	*MBOAT7*	NM_001146083:c.*549G>A;NM_001146056:c.*549G>A;NM_024298:c.*549G>A	.	.
rs34564463	19	54676814	.	.	0.156	1.96E-08	UTR5	*TMC4*	NM_001145303:c.-2C>GC;NM_144686:c.-2C>GC	.	.
rs5764047	22	44358812	.	.	-0.181	1.97E-08	intronic	*SAMM50*	.	.	.
rs2076208	22	44331060	.	.	-0.182	2.15E-08	intronic	*PNPLA3*	.	.	.
19:19404387_CA_C	19	19404387	.	.	0.162	2.15E-08	.	.	.	.	.
rs2073082	22	44360007	.	.	-0.180	2.29E-08	intronic	*SAMM50*	.	.	.
rs7245672	19	19699963	.	.	0.162	2.37E-08	intronic	*PBX4*	.	.	.
rs11668882	19	54675097	.	.	0.155	2.37E-08	intronic	*TMC4*	.	.	.
rs2073083	22	44360010	.	.	-0.179	2.69E-08	intronic	*SAMM50*	.	.	.
rs1009136	19	19440428	.	.	0.159	2.94E-08	intronic	*MAU2*	.	.	.
rs67720221	19	19440864	.	.	0.158	3.10E-08	intronic	*MAU2*	.	.	.
rs3764567	19	19440066	.	.	0.158	3.15E-08	intronic	*MAU2*	.	.	.
rs2074091	19	19450080	.	.	0.158	3.37E-08	intronic	*MAU2*	.	.	.
rs12460764	19	19431963	.	.	0.158	3.41E-08	intronic	*MAU2*	.	.	.
rs62135552	19	19436854	.	.	0.158	3.41E-08	intronic	*MAU2*	.	.	.
rs7246748	19	19433105	.	.	0.158	3.41E-08	intronic	*MAU2*	.	.	.
rs7247309	19	19439631	.	.	0.158	3.41E-08	intronic	*MAU2*	.	.	.
rs7254230	19	19434350	.	.	0.158	3.41E-08	intronic	*MAU2*	.	.	.
rs2905427	19	19478023	.	.	0.158	3.52E-08	intergenic	*MAU2;GATAD2A*	dist=8460;dist=18629	.	.
rs13964	19	19468710	.	.	0.158	3.73E-08	UTR3	*MAU2*	NM_015329:c.*2119G>C	.	.
rs15622	19	19468734	.	.	0.158	3.73E-08	UTR3	*MAU2*	NM_015329:c.*2143A>G	.	.
rs10623726	19	19382307	.	.	0.159	3.82E-08	intronic	*TM6SF2*	.	.	.
rs10640109	19	19443406	.	.	0.157	3.83E-08	.	.	.	.	.
rs2965198	19	19473030	.	.	0.157	4.09E-08	intergenic	*MAU2;GATAD2A*	dist=3467;dist=23622	.	.
rs2073088	22	44373579	.	.	-0.177	4.42E-08	intronic	*SAMM50*	.	.	.
rs2905424	19	19473445	.	.	0.157	4.50E-08	intergenic	*MAU2;GATAD2A*	dist=3882;dist=23207	.	.
rs2965175	19	19481606	.	.	0.156	4.53E-08	intergenic	*MAU2;GATAD2A*	dist=12043;dist=15046	.	.
rs34538000	19	19481379	.	.	0.156	4.53E-08	intergenic	*MAU2;GATAD2A*	dist=11816;dist=15273	.	.
rs12985655	19	19442434	.	.	0.156	4.61E-08	intronic	*MAU2*	.	.	.
rs2074090	19	19449686	.	.	0.156	4.61E-08	exonic	*MAU2*	.	synonymous SNV	MAU2:NM_015329:exon5:c.G489G:p.S163S
rs2301669	19	19453560	.	.	0.156	4.61E-08	intronic	*MAU2*	.	.	.
rs2965191	19	19453521	.	.	0.156	4.61E-08	intronic	*MAU2*	.	.	.
rs8103197	19	19443466	.	.	0.156	4.61E-08	intronic	*MAU2*	.	.	.
rs2965199	19	19475088	.	.	0.157	4.63E-08	intergenic	*MAU2;GATAD2A*	dist=5525;dist=21564	.	.
rs2315281	19	19480099	.	.	0.156	4.77E-08	intergenic	*MAU2;GATAD2A*	dist=10536;dist=16553	.	.
rs9614300	22	44362815	.	.	-0.177	4.77E-08	intronic	*SAMM50*	.	.	.
rs8101499	19	19476984	.	.	0.156	4.85E-08	intergenic	*MAU2;GATAD2A*	dist=7421;dist=19668	.	.
rs12459854	19	19461437	.	.	0.156	4.86E-08	intronic	*MAU2*	.	.	.

Table S6. Sex interaction analysis results in the Genome-wide significant SNPs from the QT GWAS.

P-values were adjusted accounting for the number of independent SNPs (n = 30) according to the LD pattern.

ID	BETA_all_I	P_all_I	ADJP
rs17217098	0.169	0.0018	0.053
rs739846	0.157	0.0018	0.055
19:19756073_AGCC_A	0.168	0.0022	0.067
rs150268548	0.162	0.0028	0.083
rs73004967	0.159	0.0032	0.095
19:19432290_AG_A	0.157	0.0034	0.102
rs58434384	0.140	0.0041	0.124
rs73001065	0.152	0.0043	0.128
rs2304130	0.139	0.0043	0.129
rs56408111	0.136	0.0066	0.198
rs72999033	0.149	0.0074	0.223
rs756350040	0.137	0.0086	0.257
19:19699398_GA_G	0.141	0.0093	0.278
rs10401969	0.124	0.0149	0.448
rs2304128	0.122	0.0167	0.500
rs58542926	0.121	0.0177	0.532
rs56255430	0.112	0.0226	0.677
rs200210321	0.115	0.0261	0.784
rs9626056	0.126	0.0271	0.814
rs8107974	0.111	0.0288	0.865
rs111393709	0.125	0.0291	0.873
rs9306471	0.123	0.0308	0.925
rs73002956	0.106	0.0315	0.946

Table continues on next page.

ID	BETA_all_I	P_all_I	ADJP
rs73004951	0.106	0.0334	1.000
rs73004962	0.104	0.0352	1.000
rs16996148	0.104	0.0363	1.000
rs58847337	0.103	0.0372	1.000
rs10500212	0.103	0.0373	1.000
rs16996185	0.103	0.0373	1.000
rs57504626	0.103	0.0373	1.000
rs73004966	0.103	0.0374	1.000
rs73004959	0.103	0.0374	1.000
rs143988316	0.104	0.0375	1.000
rs3794991	0.102	0.0385	1.000
rs12610185	0.102	0.0392	1.000
rs12610191	0.102	0.0392	1.000
rs73004975	0.101	0.0410	1.000
rs73004933	0.102	0.0418	1.000
rs73004926	0.102	0.0420	1.000
rs9626058	0.110	0.0421	1.000
rs9626061	0.111	0.0426	1.000
rs9625964	0.110	0.0431	1.000
rs9625966	0.110	0.0433	1.000
rs9626057	0.110	0.0437	1.000
rs12608729	0.100	0.0438	1.000
rs117772800	0.109	0.0448	1.000
rs12165526	0.093	0.0450	1.000
rs738494	0.108	0.0453	1.000
rs9625965	0.109	0.0460	1.000
rs9626064	0.107	0.0473	1.000
rs12167852	0.108	0.0476	1.000
rs28733632	0.107	0.0478	1.000

Table continues on next page.

ID	BETA_all_I	P_all_I	ADJP
rs28421169	0.107	0.0481	1.000
rs17216525	0.099	0.0487	1.000
rs9626071	0.106	0.0487	1.000
rs58489806	0.094	0.0491	1.000
rs873870	0.053	0.0508	1.000
rs28754570	0.105	0.0510	1.000
rs117130990	0.106	0.0512	1.000
rs12330016	0.106	0.0512	1.000
rs73434655	0.106	0.0513	1.000
rs12170274	0.105	0.0516	1.000
rs12168138	0.105	0.0517	1.000
rs9626075	0.105	0.0532	1.000
rs12168183	0.105	0.0533	1.000
rs9626076	0.104	0.0540	1.000
rs738409	0.062	0.0542	1.000
rs9626074	0.104	0.0546	1.000
rs9626073	0.104	0.0550	1.000
rs117369516	0.104	0.0550	1.000
rs738408	0.062	0.0552	1.000
rs75439392	0.103	0.0558	1.000
rs9626078	0.103	0.0564	1.000
rs9626068	0.103	0.0570	1.000
rs9626067	0.103	0.0571	1.000
rs41278873	0.101	0.0574	1.000
rs150824230	0.095	0.0578	1.000
rs9626066	0.103	0.0579	1.000
rs9626065	0.103	0.0579	1.000
rs7248200	0.051	0.0587	1.000
rs17216588	0.094	0.0595	1.000
rs12609436	0.051	0.0605	1.000

Table continues on next page.

ID	BETA_all_l	P_all_l	ADJP
rs141756246	0.094	0.0610	1.000
rs12170782	0.102	0.0618	1.000
rs10407283	0.050	0.0624	1.000
rs8141994	0.097	0.0719	1.000
rs3747207	0.057	0.0759	1.000
rs8100140	0.050	0.0818	1.000
rs2294915	0.055	0.0838	1.000
rs112902984	0.089	0.0946	1.000
rs117472787	0.089	0.0946	1.000
rs55768287	0.097	0.0951	1.000
rs9625970	0.089	0.0954	1.000
rs12166587	0.088	0.0980	1.000
rs73006914	0.101	0.1001	1.000
rs8141950	0.085	0.1105	1.000
rs2228603	0.080	0.1199	1.000
rs36010983	-0.055	0.1294	1.000
rs2073080	-0.052	0.1318	1.000
rs73008942	0.091	0.1356	1.000
rs16991236	-0.077	0.1404	1.000
rs2401514	-0.051	0.1440	1.000
rs2281298	-0.051	0.1444	1.000
rs2143571	-0.051	0.1458	1.000
rs2073079	-0.050	0.1527	1.000
rs3810444	0.082	0.1570	1.000
rs2235772	-0.043	0.1652	1.000
rs12484530	-0.066	0.1662	1.000
22:44381944_ATGGAGTCTTGCTC_A	-0.041	0.1814	1.000
rs2073084	-0.041	0.1855	1.000
rs14315	-0.039	0.2018	1.000
rs56077346	-0.040	0.2025	1.000

Table continues on next page.

ID	BETA_all_I	P_all_I	ADJP
rs2235775	-0.039	0.2038	1.000
rs2073085	-0.038	0.2115	1.000
rs80341032	0.074	0.2125	1.000
rs11090620	-0.038	0.2135	1.000
rs2073086	-0.037	0.2272	1.000
rs2294917	-0.033	0.2533	1.000
rs3827385	-0.039	0.2643	1.000
rs60204587	0.031	0.2658	1.000
rs35431065	0.041	0.2693	1.000
rs28451834	0.046	0.2695	1.000
rs4808950	-0.038	0.2774	1.000
rs12973258	-0.038	0.2830	1.000
rs12983940	-0.037	0.2930	1.000
rs565756573	-0.039	0.2958	1.000
rs34564463	0.028	0.3025	1.000
19:19510831_AT_A	-0.036	0.3053	1.000
rs1977081	0.037	0.3107	1.000
rs2301671	-0.036	0.3123	1.000
rs9304960	-0.036	0.3133	1.000
rs7252888	-0.035	0.3201	1.000
rs7250658	-0.035	0.3293	1.000
19:19431420_GT_G	-0.038	0.3307	1.000
rs2163805	-0.034	0.3318	1.000
rs2099333	-0.034	0.3324	1.000
rs2163804	-0.034	0.3331	1.000
rs6511036	-0.034	0.3339	1.000
rs641738	0.027	0.3348	1.000
19:19393106_CAAGA_C	-0.035	0.3356	1.000
rs60321073	-0.034	0.3361	1.000
rs4808960	-0.034	0.3363	1.000

Table continues on next page.

ID	BETA_all_I	P_all_I	ADJP
rs563530889	-0.034	0.3372	1.000
22:44335670_TGG_T	0.026	0.3386	1.000
rs10418051	-0.033	0.3417	1.000
rs34647936	-0.033	0.3421	1.000
rs113954889	0.042	0.3429	1.000
rs12977524	-0.034	0.3436	1.000
rs1977080	0.035	0.3443	1.000
rs4808199	-0.033	0.3475	1.000
rs11669516	-0.033	0.3478	1.000
rs751858	-0.033	0.3529	1.000
rs8105094	-0.034	0.3554	1.000
rs8105984	-0.034	0.3554	1.000
rs2074301	-0.033	0.3596	1.000
rs8108647	-0.033	0.3603	1.000
rs28478453	-0.032	0.3616	1.000
rs2074300	-0.033	0.3640	1.000
rs10421505	-0.032	0.3658	1.000
rs12982276	-0.032	0.3694	1.000
rs61061000	0.039	0.3755	1.000
rs2023883	-0.031	0.3780	1.000
rs11666553	-0.031	0.3808	1.000
rs12459676	-0.031	0.3829	1.000
rs738491	-0.026	0.3832	1.000
rs626283	0.024	0.3907	1.000
rs754255	-0.030	0.3918	1.000
rs12981405	-0.031	0.3928	1.000
rs12484700	0.031	0.3929	1.000
rs58833986	-0.031	0.3944	1.000
rs10404728	-0.030	0.3983	1.000
rs1465695	-0.030	0.3983	1.000

Table continues on next page.

ID	BETA_all_I	P_all_I	ADJP
rs10401193	-0.030	0.3989	1.000
rs10402661	-0.030	0.4000	1.000
rs12983137	-0.030	0.4000	1.000
rs1859287	-0.030	0.4000	1.000
rs7259434	-0.030	0.4000	1.000
rs12976025	-0.030	0.4005	1.000
rs4808194	-0.030	0.4005	1.000
rs17288409	-0.030	0.4008	1.000
rs756264087	-0.030	0.4026	1.000
rs775175628	-0.030	0.4049	1.000
rs11085259	-0.029	0.4071	1.000
rs2301668	-0.029	0.4081	1.000
rs757000	-0.029	0.4081	1.000
rs757001	-0.029	0.4081	1.000
rs4806498	0.023	0.4083	1.000
rs11085261	-0.029	0.4113	1.000
rs2281292	-0.023	0.4122	1.000
rs2238675	0.033	0.4135	1.000
rs10419672	-0.029	0.4135	1.000
rs6006599	-0.025	0.4144	1.000
rs60003758	-0.029	0.4162	1.000
rs139052	-0.026	0.4182	1.000
rs12972397	-0.029	0.4198	1.000
rs968525	-0.028	0.4291	1.000
rs111901094	-0.028	0.4313	1.000
rs11404084	-0.028	0.4325	1.000
rs11668882	0.022	0.4346	1.000
rs56219234	-0.024	0.4365	1.000
rs10403731	-0.028	0.4372	1.000
rs2294922	0.025	0.4395	1.000

Table continues on next page.

ID	BETA_all_I	P_all_I	ADJP
rs8736	0.021	0.4448	1.000
19:19422152_CA_C	-0.027	0.4478	1.000
rs2294919	-0.024	0.4510	1.000
rs1007863	-0.021	0.4523	1.000
rs17216693	0.031	0.4532	1.000
rs16991158	0.027	0.4546	1.000
rs4823173	0.027	0.4555	1.000
rs2076211	0.027	0.4558	1.000
rs2294433	0.027	0.4560	1.000
rs36055245	0.027	0.4570	1.000
rs2235778	-0.021	0.4577	1.000
rs10656207	-0.021	0.4587	1.000
rs4808196	-0.026	0.4603	1.000
rs1997693	0.027	0.4606	1.000
rs3788604	-0.020	0.4707	1.000
rs34755166	0.029	0.4726	1.000
rs10402308	-0.026	0.4730	1.000
rs6006473	-0.020	0.4764	1.000
rs7258508	-0.025	0.4794	1.000
rs1986095	-0.020	0.4803	1.000
rs2076208	-0.023	0.4849	1.000
rs11090617	0.025	0.4922	1.000
rs9625962	0.025	0.4926	1.000
rs12484809	0.025	0.4987	1.000
rs12485100	0.025	0.4987	1.000
rs12484801	0.025	0.5015	1.000
rs6006468	-0.018	0.5042	1.000
rs6006602	-0.018	0.5042	1.000
rs12483959	0.024	0.5069	1.000
rs11411903	0.028	0.5076	1.000

Table continues on next page.

ID	BETA_all_I	P_all_I	ADJP
rs11912828	-0.020	0.5114	1.000
rs6006469	-0.018	0.5140	1.000
rs57962361	0.028	0.5151	1.000
rs2072907	0.023	0.5226	1.000
rs2281137	0.023	0.5246	1.000
rs71313378	-0.025	0.5248	1.000
rs2281138	0.023	0.5254	1.000
rs61473277	-0.024	0.5265	1.000
rs2285628	-0.023	0.5272	1.000
rs13056638	0.023	0.5291	1.000
rs2076207	0.022	0.5323	1.000
rs2281293	0.022	0.5345	1.000
rs1883348	0.022	0.5383	1.000
rs2294927	-0.017	0.5384	1.000
rs12979148	0.025	0.5385	1.000
rs11672355	0.026	0.5407	1.000
rs2294923	-0.023	0.5450	1.000
rs9626079	-0.023	0.5450	1.000
rs11668104	0.025	0.5592	1.000
rs111234557	0.025	0.5593	1.000
rs13055874	0.021	0.5610	1.000
rs13055900	0.021	0.5610	1.000
rs2235777	-0.022	0.5668	1.000
rs4823183	-0.022	0.5668	1.000
19:19450254_CA_C	0.024	0.5731	1.000
rs12484795	0.020	0.5742	1.000
rs13054885	0.020	0.5746	1.000
rs926633	0.020	0.5782	1.000
rs2235776	-0.021	0.5787	1.000
rs2240117	0.022	0.5963	1.000

Table continues on next page.

ID	BETA_all_I	P_all_I	ADJP
rs2281135	0.019	0.6059	1.000
rs34879941	0.018	0.6066	1.000
rs2072905	0.018	0.6096	1.000
rs2072906	0.018	0.6096	1.000
rs2401512	0.018	0.6096	1.000
rs2896019	0.018	0.6096	1.000
rs2896020	0.018	0.6096	1.000
rs12167845	-0.020	0.6097	1.000
rs1010022	0.018	0.6100	1.000
rs1010023	0.018	0.6100	1.000
rs8142145	0.018	0.6100	1.000
rs73176497	0.018	0.6100	1.000
rs2073081	0.018	0.6105	1.000
rs4823176	0.018	0.6118	1.000
rs4823177	0.018	0.6118	1.000
rs4823178	0.018	0.6118	1.000
rs16991175	0.018	0.6120	1.000
rs34352134	0.018	0.6120	1.000
rs34376930	0.018	0.6120	1.000
rs35621602	0.018	0.6120	1.000
rs28550680	-0.016	0.6157	1.000
rs13056555	0.018	0.6176	1.000
rs36069781	0.018	0.6176	1.000
rs4823109	-0.019	0.6202	1.000
rs2294916	0.018	0.6204	1.000
rs4823180	0.018	0.6204	1.000
rs4823181	0.018	0.6204	1.000
rs1883349	0.018	0.6250	1.000
rs7245672	0.014	0.6256	1.000
22:44345952_AAAAAAAT_A	-0.015	0.6273	1.000

Table continues on next page.

ID	BETA_all_I	P_all_I	ADJP
rs4823108	-0.019	0.6294	1.000
rs11704562	-0.015	0.6319	1.000
rs4823179	0.017	0.6353	1.000
rs9614300	-0.015	0.6412	1.000
rs11705218	-0.015	0.6426	1.000
rs36038527	0.017	0.6443	1.000
rs7289329	-0.014	0.6524	1.000
rs9614293	-0.014	0.6548	1.000
rs2872878	-0.013	0.6559	1.000
rs5764044	-0.014	0.6570	1.000
rs2073088	-0.014	0.6574	1.000
rs5764043	-0.014	0.6574	1.000
rs5764045	-0.014	0.6574	1.000
rs1810508	0.016	0.6603	1.000
rs2008451	0.016	0.6603	1.000
rs7252981	0.012	0.6644	1.000
rs2064361	-0.014	0.6683	1.000
rs8103250	0.012	0.6708	1.000
rs9304962	0.012	0.6708	1.000
rs2401513	-0.013	0.6753	1.000
rs8108705	0.012	0.6849	1.000
rs3761077	0.018	0.6864	1.000
rs5764047	-0.012	0.7066	1.000
rs7249692	0.011	0.7088	1.000
rs9614294	-0.012	0.7124	1.000
rs113365218	-0.015	0.7151	1.000
rs3083314	-0.009	0.7358	1.000
rs2285626	0.014	0.7366	1.000
rs4823182	-0.009	0.7496	1.000
19:19438110_GTATT_G	0.010	0.7505	1.000

Table continues on next page.

ID	BETA_all_I	P_all_I	ADJP
rs2073083	-0.010	0.7615	1.000
rs2965185	0.008	0.7743	1.000
rs2073082	-0.009	0.7747	1.000
rs1474746	-0.008	0.7758	1.000
19:19404387_CA_C	-0.008	0.7804	1.000
rs67450864	-0.007	0.7950	1.000
rs2294918	-0.006	0.8232	1.000
rs2965175	-0.006	0.8240	1.000
rs34538000	-0.006	0.8240	1.000
rs8101499	-0.006	0.8310	1.000
rs1009136	-0.006	0.8338	1.000
rs2965199	-0.006	0.8355	1.000
rs2315281	-0.006	0.8398	1.000
rs35534408	-0.006	0.8432	1.000
rs2965200	0.005	0.8485	1.000
rs12052117	0.008	0.8504	1.000
rs6511028	-0.005	0.8566	1.000
rs4808937	-0.005	0.8579	1.000
rs7254748	-0.005	0.8579	1.000
rs10409234	-0.005	0.8583	1.000
rs6006594	0.005	0.8588	1.000
rs2023882	-0.005	0.8611	1.000
rs7254927	-0.005	0.8611	1.000
rs4808938	-0.005	0.8613	1.000
rs10623726	-0.005	0.8630	1.000
rs1010207	-0.005	0.8714	1.000
rs10407952	-0.005	0.8724	1.000
rs13964	-0.004	0.8754	1.000
rs15622	-0.004	0.8754	1.000
rs2301786	-0.004	0.8761	1.000

Table continues on next page.

ID	BETA_all_I	P_all_I	ADJP
rs2315022	-0.004	0.8761	1.000
rs12459854	-0.004	0.8789	1.000
rs12460764	-0.004	0.8809	1.000
rs62135552	-0.004	0.8809	1.000
rs7246748	-0.004	0.8809	1.000
rs7247309	-0.004	0.8809	1.000
rs7254230	-0.004	0.8809	1.000
rs8182472	0.006	0.8827	1.000
rs12985655	-0.004	0.8848	1.000
rs2074090	-0.004	0.8848	1.000
rs2301669	-0.004	0.8848	1.000
rs2965191	-0.004	0.8848	1.000
rs8103197	-0.004	0.8848	1.000
19:19394249_GTT_G	-0.004	0.8872	1.000
rs67720221	-0.004	0.8955	1.000
rs113460678	0.005	0.8972	1.000
rs34324111	0.005	0.8972	1.000
rs35629458	0.005	0.8972	1.000
rs28720066	0.005	0.8994	1.000
rs2285859	-0.004	0.9020	1.000
rs57009615	-0.005	0.9029	1.000
rs12977937	-0.003	0.9030	1.000
rs4808942	-0.003	0.9030	1.000
rs10415849	0.005	0.9041	1.000
19:19668338_GC_G	0.005	0.9044	1.000
rs3764567	-0.003	0.9114	1.000
rs10408875	0.005	0.9123	1.000
rs10408596	0.005	0.9134	1.000
rs56241616	0.004	0.9140	1.000
rs59148799	0.004	0.9151	1.000

Table continues on next page.

ID	BETA_all_l	P_all_l	ADJP
rs139051	-0.003	0.9171	1.000
rs2017964	-0.003	0.9199	1.000
rs2301784	-0.003	0.9199	1.000
rs6511026	-0.003	0.9199	1.000
rs735273	-0.003	0.9199	1.000
rs8101938	-0.003	0.9199	1.000
rs8103496	-0.003	0.9199	1.000
rs56397647	0.004	0.9208	1.000
rs10419245	-0.003	0.9222	1.000
rs2074303	-0.003	0.9222	1.000
rs2315025	-0.003	0.9232	1.000
rs2269873	-0.003	0.9272	1.000
rs2074299	-0.003	0.9278	1.000
rs2315024	-0.003	0.9299	1.000
rs1883350	-0.003	0.9315	1.000
rs10426780	-0.002	0.9326	1.000
rs56273306	0.004	0.9326	1.000
rs2074296	-0.002	0.9330	1.000
rs73002960	0.003	0.9343	1.000
rs2965198	-0.002	0.9351	1.000
rs2905424	-0.002	0.9362	1.000
rs2074091	-0.002	0.9396	1.000
rs2074298	-0.002	0.9532	1.000
rs1474744	-0.001	0.9621	1.000
rs2092501	-0.001	0.9677	1.000
rs2905427	-0.001	0.9682	1.000
rs10640109	-0.001	0.9688	1.000
rs34912062	-0.001	0.9695	1.000
rs11668386	-0.001	0.9719	1.000
rs10424702	-0.001	0.9765	1.000

Table continues on next page.

ID	BETA_all_I	P_all_I	ADJP
rs56373884	-0.001	0.9807	1.000
rs3761472	0.001	0.9816	1.000
rs1474745	0.000	0.9903	1.000
rs188552254	0.000	0.9908	1.000
rs79954596	0.000	0.9961	1.000
rs2294921	0.000	0.9967	1.000

Table S7. GWAS Fine mapping analysis. Only variants with PIP > 0.5 are included

Analysis	ID	CHR	BP	Gene	Ref	Alt	MAF	Beta	SE	Z	PIP	log10bf	mean	sd	mean_incl	sd_incl
CC	rs57009615	19	19613622	*GATAD2A*	G	A	0.130	0.449	0.044	10.156	1.000	11.818	-0.831	0.055	-0.831	0.055
CC	rs2240117	19	19418916	*SUGP1*	T	C	0.127	0.495	0.042	11.732	1.000	11.818	-0.921	0.067	-0.921	0.067
CC	rs2285628	19	19467996	*MAU2*	A	T	0.173	0.355	0.045	7.956	1.000	7.297	0.673	0.059	0.673	0.059
QT	rs200210321	19	19393890	*SUGP1*	AG	A	0.073	0.766	0.052	14.751	0.523	2.196	0.197	0.190	0.377	0.039
CC	rs8107974	19	19388500	*SUGP1*	T	A	0.076	0.855	0.034	25.463	0.512	1.839	0.823	0.805	1.607	0.058

Table S8. Significant results from the MAGMA analysis.

A: Case control analysis

Symbol	Entrez ID	CHR	START	STOP	NSNPS	ZSTAT	P	adjp
PBX4	80714	19	19662516	19739725	94	7.2574	2.0E-13	3.6E-09
PNPLA3	80339	22	44309619	44353451	123	6.1094	5.0E-10	9.0E-06
SAMM50	25813	22	44341261	44402412	188	6.1094	5.0E-10	9.0E-06
LPAR2	9170	19	19724464	19749039	25	5.8246	2.9E-09	5.2E-05
SUGP1	57794	19	19377320	19441321	96	5.6705	7.1E-09	1.3E-04
MAU2	23383	19	19421496	19479563	101	5.602	1.1E-08	1.9E-04
GATAD2A	54815	19	19486642	19629741	156	4.8311	6.8E-07	1.2E-02
TM6SF2	53345	19	19364841	19394074	49	4.6655	1.5E-06	2.8E-02

B: Quantitative analysis

Symbol	Entrez ID	CHR	START	STOP	NSNPS	ZSTAT	P	adjp
LPAR2	9170	19	19724464	19749039	25	7.665	8.9E-15	1.6E-10
MAU2	23383	19	19421496	19479563	101	7.623	1.2E-14	2.2E-10
SUGP1	57794	19	19377320	19441321	96	7.594	1.6E-14	2.8E-10
PNPLA3	80339	22	44309619	44353451	123	7.393	7.2E-14	1.3E-09
GATAD2A	54815	19	19486642	19629741	156	7.076	7.4E-13	1.3E-08
CILP2	148113	19	19639057	19667468	41	6.638	1.6E-11	2.9E-07
TM6SF2	53345	19	19364841	19394074	49	6.315	1.4E-10	2.4E-06
PBX4	80714	19	19662516	19739725	94	6.109	5.0E-10	9.0E-06
SAMM50	25813	22	44341261	44402412	188	6.109	5.0E-10	9.0E-06
PARVB	29780	22	44385091	44575113	468	5.837	2.7E-09	4.8E-05
NCAN	1463	19	19312773	19373061	64	5.382	3.7E-08	6.6E-04
MBOAT7	79143	19	54667106	54703733	57	5.234	8.3E-08	1.5E-03
GMIP	51291	19	19730285	19764457	32	5.180	1.1E-07	2.0E-03
RFXANK	8625	19	19293008	19322678	47	5.111	1.6E-07	2.9E-03
TMC4	147798	19	54653833	54686944	53	5.098	1.7E-07	3.1E-03
HAPLN4	404037	19	19356450	19383596	44	4.774	9.1E-07	1.6E-02
MEF2B	4207	19	19246376	19313400	172	4.677	1.5E-06	2.6E-02
TSSK6	83983	19	19615028	19636469	29	4.663	1.6E-06	2.8E-02
MEF2B	100271849	19	19246376	19291098	132	4.580	2.3E-06	4.2E-02

Table S9. Top 15 GO functional classes obtained from the MAGMA Gene-Set enrichment analysis for the Case-Control (A) and Quantitative GWAS (B).

A: Case control MAGMA analysis

FULL_NAME	NGENES	BETA	SE	P	BH
SRP DEPENDENT COTRANSLATIONAL PROTEIN TARGETING TO MEMBRANE	17	0.915	0.212	7.92E-06	0.083
ATP BINDING CASSETTE ABC TRANSPORTER COMPLEX	5	1.762	0.412	9.31E-06	0.097
SRP DEPENDENT COTRANSLATIONAL PROTEIN TARGETING TO MEMBRANE TRANSLOCATION	8	1.190	0.286	1.60E-05	0.167
INTRACILIARY TRANSPORT INVOLVED IN CILIUM ASSEMBLY	7	1.293	0.342	7.77E-05	0.811
PROTEIN TRANSMEMBRANE TRANSPORT	63	0.393	0.110	1.70E-04	1.000
POSITIVE REGULATION OF EPIDERMAL GROWTH FACTOR ACTIVATED RECEPTOR ACTIVITY	5	1.032	0.298	2.66E-04	1.000
COTRANSLATIONAL PROTEIN TARGETING TO MEMBRANE	22	0.630	0.191	4.78E-04	1.000
PEPTIDE ANTIGEN BINDING	34	0.550	0.169	5.74E-04	1.000
INTRACELLULAR PROTEIN TRANSMEMBRANE TRANSPORT	51	0.388	0.120	6.26E-04	1.000
REGULATION OF ESTABLISHMENT OR MAINTENANCE OF CELL POLARITY	27	0.519	0.163	7.39E-04	1.000
NEGATIVE REGULATION OF PROTEIN TARGETING TO MEMBRANE	4	1.365	0.432	7.95E-04	1.000
CHITOSOME	19	0.594	0.188	7.98E-04	1.000
SULFUR COMPOUND TRANSPORT	52	0.360	0.114	8.00E-04	1.000
ESTABLISHMENT OF PROTEIN LOCALIZATION TO ENDOPLASMIC RETICULUM	40	0.420	0.135	9.62E-04	1.000
NEGATIVE REGULATION OF ACTIN NUCLEATION	9	0.849	0.281	1.27E-03	1.000

B: Quantitative MAGMA analysis

FULL_NAME	NGENES	BETA	SE	P	BH
INTRACILIARY TRANSPORT INVOLVED IN CILIUM ASSEMBLY	7	1.288	0.343	8.86E-05	0.926
VERY LOW DENSITY LIPOPROTEIN PARTICLE ASSEMBLY	12	1.057	0.285	1.06E-04	1.000
MOLYBDOPTERIN COFACTOR BINDING	6	1.296	0.356	1.39E-04	1.000
SECONDARY METABOLIC PROCESS	54	0.395	0.112	2.03E-04	1.000
DOUBLE STRANDED TELOMERIC DNA BINDING	9	0.892	0.271	4.92E-04	1.000
TRANSMEMBRANE TRANSPORT	1441	0.080	0.024	4.98E-04	1.000
O ACYLTRANSFERASE ACTIVITY	50	0.379	0.120	7.74E-04	1.000
INSULIN RECEPTOR SUBSTRATE BINDING	13	0.705	0.227	9.78E-04	1.000
LOW DENSITY LIPOPROTEIN RECEPTOR PARTICLE METABOLIC PROCESS	5	1.341	0.442	1.22E-03	1.000
NEGATIVE REGULATION OF ACTIN NUCLEATION	9	0.855	0.282	1.23E-03	1.000
ROUNDABOUT SIGNALING PATHWAY	5	1.489	0.493	1.26E-03	1.000
ACID THIOL LIGASE ACTIVITY	30	0.442	0.148	1.40E-03	1.000
VENTRICULAR SYSTEM DEVELOPMENT	32	0.470	0.164	2.12E-03	1.000

Table S10. Results of the Transcription-Wide Association Study. In bold, genes significant after multiple testing correction.

TWAS fine mapping was conducted only on the regions including significant genes, and considering genome-wide significance SNPs, except for MBOAT7 in the CC-GWAS (p < 1.0E-05).

Gene coordinates are according to Hg38 assembly, derived by the GTEx v8 weights.

A: case-control TWAS

Gene	Chromosome	P0	P1	Tissue reference panel	TWAS.Z	TWAS.P	Adjusted TWAS-P	JOINT-P*	Fine Mapping PIP
MBOAT7	**19**	**54189881**	**54189882**	**GTExv8.EUR.Liver**	**-5.025**	**5.0E-07**	**1.9E-03**	**5.00E-07**	**0.748**
TMC4	19	54173249	54173250	GTExv8.EUR.Liver	-4.188	2.8E-05	1.0E-01	-	
SAMM50	22	43955420	43955421	GTExv8.EUR.Liver	3.700	2.2E-04	8.1E-01	-	
SKAP1	17	48430274	48430275	GTExv8.EUR.Liver	-3.654	2.6E-04	9.6E-01	-	
IGHMBP2	11	68903841	68903842	GTExv8.EUR.Liver	-3.652	2.6E-04	9.7E-01	-	
SLC17A4	6	25754698	25754699	GTExv8.EUR.Liver	-3.623	2.9E-04	1.0E+00	-	
BDH2	4	103099882	103099883	GTExv8.EUR.Liver	3.613	3.0E-04	1.0E+00	-	
FAM216A	12	110468375	110468376	GTExv8.EUR.Liver	3.479	5.0E-04	1.0E+00	-	
UBAP2L	1	154220178	154220179	GTExv8.EUR.Liver	3.458	5.5E-04	1.0E+00	-	
THBS3	1	155207980	155207981	GTExv8.EUR.Liver	3.417	6.3E-04	1.0E+00	-	

B: quantitative TWAS

Gene	CHR	P0	P1	Tissue reference panel	TWAS.Z	TWAS.P	Adjusted TWAS-P	JOINT.P*	Fine Mapping PIP
MBOAT7	**19**	**54189881**	**54189882**	**GTExv8.EUR.Liver**	**-5.869**	**4.4E-09**	**1.6E-05**	**4.40E-09**	**0.395**
SAMM50	**22**	**43955420**	**43955421**	**GTExv8.EUR.Liver**	**4.946**	**7.6E-07**	**2.8E-03**	**7.60E-07**	**1.000**
IGHMBP2	11	68903841	68903842	GTExv8.EUR.Liver	-4.311	1.6E-05	6.0E-02	1.60E-05	
ENSG00000224251	10	4995487	4995488	GTExv8.EUR.Liver	-4.021	5.8E-05	2.2E-01	-	
MRPL21	11	68903834	68903835	GTExv8.EUR.Liver	4.007	6.2E-05	2.3E-01	-	
TMC4	19	54173249	54173250	GTExv8.EUR.Liver	-4.000	6.3E-05	2.4E-01	-	
LINC02701	11	68870663	68870664	GTExv8.EUR.Liver	3.899	9.7E-05	3.6E-01	-	
LIG3	17	34980493	34980494	GTExv8.EUR.Liver	-3.670	2.4E-04	9.1E-01	-	
ALOX5	10	45374175	45374176	GTExv8.EUR.Liver	3.611	3.1E-04	1.1E+00	-	
RAB8A	19	16111628	16111629	GTExv8.EUR.Liver	3.578	3.5E-04	1.0E+00	-	

*Joint conditional testing has been only conducted on Bonferroni adjusted suggestive genes (adj-p < 0.10)

Table S11. Gene Set Enrichment Analysis conducted using the TWAS results.

A: Case-control GWAS

ONTOLOGY	ID	Description	enrichmentScore	NES	pvalue	p.adjust
MF	GO:0019903	protein phosphatase binding	-0.649	-2.051	2.34E-04	0.250
CC	GO:0000315	organellar large ribosomal subunit	0.761	1.984	2.85E-04	0.250
CC	GO:0005762	mitochondrial large ribosomal subunit	0.761	1.984	2.85E-04	0.250
BP	GO:0006886	intracellular protein transport	0.348	1.605	1.08E-03	0.553
BP	GO:0090150	establishment of protein localization to membrane	0.520	1.819	1.45E-03	0.553
CC	GO:0005694	chromosome	0.316	1.535	1.51E-03	0.553
BP	GO:0045184	establishment of protein localization	0.291	1.457	1.79E-03	0.553
BP	GO:0002819	regulation of adaptive immune response	-0.562	-1.847	1.99E-03	0.553
BP	GO:0034440	lipid oxidation	0.657	1.865	2.10E-03	0.553
BP	GO:0032388	positive regulation of intracellular transport	0.588	1.794	3.22E-03	0.625
BP	GO:0050864	regulation of B cell activation	-0.621	-1.846	3.32E-03	0.625
BP	GO:0009266	response to temperature stimulus	0.593	1.785	3.71E-03	0.625
BP	GO:0002637	regulation of immunoglobulin production	-0.681	-1.778	4.19E-03	0.625
CC	GO:0097729	9+2 motile cilium	-0.508	-1.741	4.49E-03	0.625
BP	GO:0008654	phospholipid biosynthetic process	-0.461	-1.699	5.01E-03	0.625

B: quantitative GWAS

ONTOLOGY	ID	Description	enrichmentScore	NES	pvalue	p.adjust
MF	GO:0019903	protein phosphatase binding	-0.621	-1.931	1.45E-03	0.999
BP	GO:0009266	response to temperature stimulus	0.628	1.872	2.07E-03	0.999
BP	GO:0032436	ation of proteasomal ubiquitin-dependent protein cat	0.704	1.820	2.09E-03	0.999
MF	GO:0015399	primary active transmembrane transporter activity	-0.684	-1.819	2.67E-03	0.999
BP	GO:0002637	regulation of immunoglobulin production	-0.701	-1.825	2.96E-03	0.999
BP	GO:0006403	RNA localization	-0.504	-1.730	3.39E-03	0.999
MF	GO:0016835	carbon-oxygen lyase activity	-0.625	-1.779	3.53E-03	0.999
BP	GO:0002250	adaptive immune response	-0.428	-1.678	3.62E-03	0.999
MF	GO:0016758	hexosyltransferase activity	0.477	1.693	3.87E-03	0.999
CC	GO:0000315	organellar large ribosomal subunit	0.671	1.736	4.55E-03	0.999
CC	GO:0005762	mitochondrial large ribosomal subunit	0.671	1.736	4.55E-03	0.999
BP	GO:0048193	Golgi vesicle transport	0.422	1.621	5.15E-03	0.999
BP	GO:1901214	regulation of neuron death	0.534	1.701	5.46E-03	0.999
BP	GO:0034440	lipid oxidation	0.616	1.710	6.52E-03	0.999
BP	GO:0090150	establishment of protein localization to membrane	0.486	1.681	6.97E-03	0.999

Table S12. Overlap between our CC-GWAS and the finding identified in Sun et al. (p < 5.0E-08). Variants were matched by chromosome and position.

ID	Chr	POS	REF	ALT	Func.refGene	Gene.refGene	GeneDetail.refGene	ExonicFunc.refGene	AAChange.refGene	P (current study)	P (Sun et al)
rs738408	22	44324730	C	T	exonic	PNPLA3	.	synonymous SNV	PNPLA3:NM_025225:exon3:c.C447T:p.P149P	5.4E-30	8.4E-13
rs738409	22	44324727	C	G	exonic	PNPLA3	.	nonsynonymous SNV	PNPLA3:NM_025225:exon3:c.C444G:p.I148M	6.6E-30	8.6E-13
rs3747207	22	44324855	G	A	intronic	PNPLA3	.	.	.	6.9E-30	1.2E-12
rs2294915	22	44340904	C	T	intronic	PNPLA3	.	.	.	1.2E-28	9.1E-12
rs58542926	19	19379549	C	T	exonic	TM6SF2	.	nonsynonymous SNV	TM6SF2:NM_001001524:exon6:c.G499A:p.E167K	1.5E-27	4.7E-14
rs200210321	19	19393890	A	AG	intronic	SUGP1	.	.	.	1.5E-27	3.3E-14
rs8107974	19	19388500	A	T	intronic	SUGP1	.	.	.	2.5E-27	1.4E-13
rs756350040	19	19370340	TGACA	T	.	.	.	.	.	3.8E-27	1.3E-13
rs10401969	19	19407718	T	C	intronic	SUGP1	.	.	.	8.4E-27	5.4E-14
rs58489806	19	19456917	C	T	intronic	MAU2	.	.	.	4.5E-25	5.4E-15
rs2294922	22	44379565	G	C	intronic	SAMM50	.	.	.	4.8E-25	3.8E-08
19:19432290_AG_A	19	19432290	AG	A	.	.	.	.	.	1.0E-24	2.0E-13
rs739846	19	19419071	G	A	intronic	SUGP1	.	.	.	1.2E-24	5.6E-14
rs73001065	19	19460541	G	C	intronic	MAU2	.	.	.	5.3E-24	4.7E-13
rs150268548	19	19494483	G	A	intergenic	MAU2;GATAD2A	dist=24920;dist=2169	.	.	2.0E-23	1.1E-12
rs56255430	19	19477877	A	C	intergenic	MAU2;GATAD2A	dist=8314;dist=18775	.	.	1.6E-22	2.7E-12
rs72999033	19	19366632	C	T	UTR3	HAPLN4	NM_023002:c.*1994G>A	.	.	5.1E-22	1.6E-11
rs17217098	19	19702384	G	A	intronic	PBX4	.	.	.	1.2E-21	5.6E-12
19:19699398_GA_G	19	19699398	GA	G	.	.	.	.	.	3.7E-21	6.5E-12
rs2228603	19	19329924	C	T	exonic	NCAN	.	nonsynonymous SNV	NCAN:NM_004386:exon3:c.C274T:p.P92S	1.0E-20	2.9E-12
rs73004967	19	19717056	A	G	intronic	PBX4	.	.	.	1.5E-20	6.5E-12
rs3794991	19	19610596	C	T	intronic	GATAD2A	.	.	.	6.0E-20	2.3E-12
rs73002956	19	19578743	A	G	intronic	GATAD2A	.	.	.	6.1E-20	3.9E-12
rs150824230	19	19670610	G	A	intergenic	CILP2;PBX4	dist=13142;dist=1912	.	.	2.5E-19	2.5E-11
rs73004926	19	19671266	C	T	intergenic	CILP2;PBX4	dist=13798;dist=1256	.	.	3.7E-19	2.1E-11
rs73004933	19	19675696	C	T	intronic	PBX4	.	.	.	3.8E-19	2.2E-11
rs17216525	19	19662220	C	T	intergenic	CILP2;PBX4	dist=4752;dist=10302	.	.	4.0E-19	3.4E-10
rs36038527	22	44332888	T	TC	intronic	PNPLA3	.	.	.	4.7E-19	5.0E-11
rs141756246	19	19685470	G	GT	intronic	PBX4	.	.	.	4.9E-19	1.9E-11
rs12485100	22	44325516	G	T	intronic	PNPLA3	.	.	.	5.2E-19	5.2E-11
rs12484809	22	44325631	C	T	intronic	PNPLA3	.	.	.	5.2E-19	5.4E-11
rs12484801	22	44325565	C	T	intronic	PNPLA3	.	.	.	5.3E-19	5.2E-11
rs143988316	19	19667254	C	T	intergenic	CILP2;PBX4	dist=9786;dist=5268	.	.	5.8E-19	9.3E-11
rs11090617	22	44326700	C	T	intronic	PNPLA3	.	.	.	6.1E-19	5.7E-11
rs9625962	22	44326272	T	C	intronic	PNPLA3	.	.	.	6.2E-19	6.0E-11
rs12483959	22	44325996	G	A	intronic	PNPLA3	.	.	.	6.2E-19	5.6E-11
rs4823179	22	44341193	T	C	intronic	PNPLA3	.	.	.	7.0E-19	5.2E-11
rs73176497	22	44336957	G	A	intronic	PNPLA3	.	.	.	7.4E-19	4.5E-11
rs2281135	22	44332570	G	A	intronic	PNPLA3	.	.	.	8.0E-19	3.4E-11
rs1997693	22	44331513	C	G	intronic	PNPLA3	.	.	.	8.3E-19	1.9E-11
rs13056555	22	44339526	C	G	intronic	PNPLA3	.	.	.	8.4E-19	6.8E-11
rs36069781	22	44340086	C	T	intronic	PNPLA3	.	.	.	8.4E-19	6.9E-11
rs4823181	22	44341606	T	C	intronic	PNPLA3	.	.	.	8.5E-19	5.4E-11
rs2072906	22	44333172	A	G	intronic	PNPLA3	.	.	.	8.5E-19	4.2E-11
rs4823177	22	44334486	T	C	intronic	PNPLA3	.	.	.	8.6E-19	4.9E-11
rs4823178	22	44334529	T	C	intronic	PNPLA3	.	.	.	8.6E-19	4.9E-11
rs4823176	22	44334476	T	C	intronic	PNPLA3	.	.	.	8.7E-19	4.9E-11
rs2294916	22	44340922	T	G	intronic	PNPLA3	.	.	.	8.7E-19	5.2E-11
rs4823180	22	44341298	G	A	intronic	PNPLA3	.	.	.	8.7E-19	5.3E-11
rs2072905	22	44333479	C	G	intronic	PNPLA3	.	.	.	8.7E-19	4.4E-11
rs2896019	22	44333694	T	G	intronic	PNPLA3	.	.	.	8.7E-19	4.6E-11
rs2401512	22	44333945	C	G	intronic	PNPLA3	.	.	.	8.7E-19	4.7E-11
rs2896020	22	44333968	T	C	intronic	PNPLA3	.	.	.	8.7E-19	4.7E-11
rs34879941	22	44332878	C	T	intronic	PNPLA3	.	.	.	8.7E-19	3.9E-11
rs16991175	22	44335331	T	C	intronic	PNPLA3	.	.	.	8.8E-19	5.3E-11
rs35621602	22	44335406	C	A	intronic	PNPLA3	.	.	.	8.8E-19	5.3E-11
rs34352134	22	44335416	C	T	intronic	PNPLA3	.	.	.	8.8E-19	5.3E-11
rs34376930	22	44335453	G	T	intronic	PNPLA3	.	.	.	8.8E-19	5.3E-11
rs2073081	22	44335744	T	C	intronic	PNPLA3	.	.	.	9.0E-19	5.5E-11
rs1010023	22	44336098	T	C	intronic	PNPLA3	.	.	.	9.3E-19	5.3E-11

Table continues on next page.

ID	Chr	POS	REF	ALT	Func.refGene	Gene.refGene	GeneDetail.refGene	ExonicFunc.refGene	AAChange.refGene	P (current study)	P (Sun et al)
rs1010022	22	44336310	A	G	intronic	PNPLA3	.	.	.	9.3E-19	5.4E-11
rs8142145	22	44336496	T	C	intronic	PNPLA3	.	.	.	9.3E-19	5.3E-11
rs17216588	19	19664077	C	T	intergenic	CILP2;PBX4	dist=6609;dist=8445	.	.	9.7E-19	1.3E-10
rs16996148	19	19658472	G	T	intergenic	CILP2;PBX4	dist=1004;dist=14050	.	.	9.8E-19	1.2E-10
rs1883349	22	44331943	G	A	intronic	PNPLA3	.	.	.	1.1E-18	3.4E-11
rs2294433	22	44329275	G	A	intronic	PNPLA3	.	.	.	1.2E-18	6.4E-11
rs2304128	19	19746151	G	T	intronic	GMIP	.	.	.	1.2E-18	7.1E-10
rs926633	22	44337533	G	A	intronic	PNPLA3	.	.	.	1.3E-18	6.7E-11
rs13056638	22	44331778	C	G	intronic	PNPLA3	.	.	.	1.3E-18	2.0E-11
rs73004962	19	19713069	A	T	intronic	PBX4	.	.	.	1.3E-18	3.1E-11
rs2072907	22	44332653	C	G	intronic	PNPLA3	.	.	.	1.4E-18	2.2E-11
rs2076207	22	44333370	A	G	intronic	PNPLA3	.	.	.	1.4E-18	2.7E-11
rs2281138	22	44332477	T	C	intronic	PNPLA3	.	.	.	1.4E-18	2.3E-11
rs2281293	22	44334842	T	C	intronic	PNPLA3	.	.	.	1.4E-18	3.1E-11
rs2281137	22	44332493	T	C	intronic	PNPLA3	.	.	.	1.4E-18	2.4E-11
rs12608729	19	19700552	C	T	intronic	PBX4	.	.	.	1.5E-18	2.9E-11
rs1883348	22	44331815	C	G	intronic	PNPLA3	.	.	.	1.5E-18	2.1E-11
rs1977080	22	44330031	C	T	intronic	PNPLA3	.	.	.	1.5E-18	5.2E-11
rs73004951	19	19695228	C	T	intronic	PBX4	.	.	.	1.8E-18	2.0E-11
rs2008451	22	44342969	T	C	UTR3	PNPLA3	NM_025225:c.*707T>C	.	.	1.9E-18	5.9E-11
rs1810508	22	44343151	A	G	UTR3	PNPLA3	NM_025225:c.*889A>G	.	.	1.9E-18	6.0E-11
rs13055900	22	44341666	A	G	intronic	PNPLA3	.	.	.	2.0E-18	5.9E-11
rs13055874	22	44341672	T	C	intronic	PNPLA3	.	.	.	2.0E-18	6.0E-11
rs56373884	22	44356468	G	A	intronic	SAMM50	.	.	.	2.0E-18	7.0E-10
rs4823173	22	44328730	G	A	intronic	PNPLA3	.	.	.	2.2E-18	5.9E-11
rs2076211	22	44329078	C	T	intronic	PNPLA3	.	.	.	2.2E-18	6.1E-11
rs13054885	22	44345771	G	A	intergenic	PNPLA3;SAMM50	dist=2309;dist=5551	.	.	2.4E-18	6.3E-11
rs2092501	22	44347251	G	A	intergenic	PNPLA3;SAMM50	dist=3789;dist=4071	.	.	2.4E-18	5.6E-10
rs16991158	22	44327179	G	A	intronic	PNPLA3	.	.	.	2.4E-18	5.8E-11
rs36055245	22	44327192	A	G	intronic	PNPLA3	.	.	.	2.5E-18	5.8E-11
rs1977081	22	44330128	T	C	intronic	PNPLA3	.	.	.	2.5E-18	2.7E-11
rs12484700	22	44327273	A	G	intronic	PNPLA3	.	.	.	2.6E-18	3.3E-11
rs2294921	22	44361842	C	T	intronic	SAMM50	.	.	.	2.6E-18	1.1E-09
rs1474745	22	44349236	T	C	intergenic	PNPLA3;SAMM50	dist=5774;dist=2086	.	.	2.7E-18	8.2E-10
rs12610185	19	19721722	G	A	intronic	PBX4	.	.	.	2.7E-18	4.6E-11
rs12610191	19	19721976	C	T	intronic	PBX4	.	.	.	2.7E-18	4.6E-11
rs58847337	19	19726022	G	A	intronic	PBX4	.	.	.	3.0E-18	4.6E-11
rs57504626	19	19720399	C	T	intronic	PBX4	.	.	.	3.1E-18	5.0E-11
rs16996185	19	19720788	T	G	intronic	PBX4	.	.	.	3.1E-18	5.0E-11
rs10500212	19	19723215	C	T	intronic	PBX4	.	.	.	3.1E-18	4.8E-11
rs73004966	19	19716558	C	T	intronic	PBX4	.	.	.	3.5E-18	3.7E-11
rs34912062	22	44348446	G	T	intergenic	PNPLA3;SAMM50	dist=4984;dist=2876	.	.	3.7E-18	8.7E-10
rs3761472	22	44368122	A	G	exonic	SAMM50	.	nonsynonymous SNV	SAMM50:NM_015380:exon5:c.A329G:p.D110G	3.9E-18	1.7E-09
rs73004975	19	19727152	A	G	intronic	PBX4	.	.	.	4.3E-18	4.2E-11
rs73004959	19	19711139	C	T	intronic	PBX4	.	.	.	4.5E-18	2.3E-11
rs12484795	22	44343626	A	C	downstream	PNPLA3	dist=164	.	.	5.4E-17	1.6E-10
rs57962361	19	19425025	C	T	intronic	SUGP1	.	.	.	4.5E-16	3.4E-11
rs111234557	19	19436229	C	G	intronic	MAU2	.	.	.	6.5E-16	3.7E-11
rs11668104	19	19426181	G	A	intronic	SUGP1	.	.	.	9.1E-16	4.8E-11
rs11672355	19	19462702	G	C	intronic	MAU2	.	.	.	1.1E-15	6.0E-11
rs11411903	19	19440064	T	TA	intronic	MAU2	.	.	.	1.3E-15	5.5E-11
19:19450254_CA_C	19	19450254	CA	C	.	.	.	.	.	1.1E-14	3.7E-11
19:19756073_AGCC_A	19	19756073	AGCC	A	.	.	.	.	.	1.1E-13	3.8E-09
rs2285626	19	19467545	C	T	UTR3	MAU2	NM_015329:c.*954C>T	.	.	1.2E-13	1.4E-10
rs56408111	19	19793545	T	C	UTR3	ZNF101	NM_001300949:c.*2436T>C;NM_033204:c.*2436T>C	.	.	1.6E-13	1.0E-10
rs58434384	19	19786099	A	G	intronic	ZNF101	.	.	.	3.1E-13	1.1E-10
rs2304130	19	19789528	A	G	intronic	ZNF101	.	.	.	3.5E-13	1.1E-10
rs12052117	19	19485105	C	T	intergenic	MAU2;GATAD2A	dist=15542;dist=11547	.	.	6.9E-13	3.5E-10
rs73002960	19	19582992	C	T	intronic	GATAD2A	.	.	.	1.4E-12	2.4E-10

Table continues on next page.

ID	Chr	POS	REF	ALT	Func.refGene	Gene.refGene	GeneDetail.refGene	ExonicFunc.refGene	AAChange.refGene	P (current study)	P (Sun et al)
rs28720066	19	19572220	G	T	intronic	GATAD2A	.	.	.	1.6E-12	2.7E-10
rs59148799	19	19484008	A	G	intergenic	MAU2;GATAD2A	dist=14445;dist=12644	.	.	2.0E-12	3.4E-10
rs56241616	19	19506092	C	T	intronic	GATAD2A	.	.	.	2.1E-12	4.4E-10
rs10408875	19	19503573	T	C	intronic	GATAD2A	.	.	.	2.1E-12	4.4E-10
rs10408596	19	19512657	A	T	intronic	GATAD2A	.	.	.	2.2E-12	4.4E-10
rs8182472	19	19539891	T	C	intronic	GATAD2A	.	.	.	2.2E-12	2.3E-10
rs10415849	19	19505087	C	T	intronic	GATAD2A	.	.	.	2.3E-12	4.3E-10
rs56273306	19	19621004	T	C	intergenic	GATAD2A;TSSK6	dist=1263;dist=4024	.	.	2.3E-12	2.0E-10
rs34324111	19	19513568	T	G	intronic	GATAD2A	.	.	.	3.1E-12	3.1E-10
rs35629458	19	19513572	T	G	intronic	GATAD2A	.	.	.	3.1E-12	3.1E-10
rs113460678	19	19513580	T	G	intronic	GATAD2A	.	.	.	3.1E-12	3.1E-10
rs56397647	19	19642795	C	T	intronic	YJEFN3	.	.	.	2.5E-11	9.3E-10
rs11668386	19	19531910	A	G	intronic	GATAD2A	.	.	.	4.4E-11	1.2E-09
rs113365218	19	19621197	G	A	intergenic	GATAD2A;TSSK6	dist=1456;dist=3831	.	.	5.6E-11	2.9E-10
rs10424702	19	19508013	A	G	intronic	GATAD2A	.	.	.	6.5E-11	1.5E-09
rs188552254	19	19517169	A	G	intronic	GATAD2A	.	.	.	8.3E-11	1.4E-09
rs79954596	19	19548643	T	G	intronic	GATAD2A	.	.	.	9.0E-11	1.3E-09
rs57009615	19	19613622	A	G	intronic	GATAD2A	.	.	.	9.2E-11	5.9E-10

Table S13. Overlap between our QT-GWAS and the finding identified in Sun et al. (p < 5.0E-08). Variants were matched by chromosome and position.

ID	Chr	POS	REF	ALT	Func.refGene	Gene.refGene	GeneDetail.refGene	ExonicFunc.refGene	AAChange.refGene	P (current study)	P (Sun et al)
rs738408	22	44324730	C	T	exonic	PNPLA3	.	synonymous SNV	PNPLA3:NM_025225:exon3:c.C447T:p.P149P	4.3E-60	8.4E-13
rs738409	22	44324727	C	G	exonic	PNPLA3	.	nonsynonymous SNV	PNPLA3:NM_025225:exon3:c.C444G:p.I148M	5.3E-60	8.6E-13
rs3747207	22	44324855	G	A	intronic	PNPLA3	.	.	.	7.4E-60	1.2E-12
rs2294915	22	44340904	C	T	intronic	PNPLA3	.	.	.	7.2E-58	9.1E-12
rs200210321	19	19393890	A	AG	intronic	SUGP1	.	.	.	8.9E-49	3.3E-14
rs58542926	19	19379549	C	T	exonic	TM6SF2	.	nonsynonymous SNV	TM6SF2:NM_001001524:exon6:c.G499A:p.E167K	2.2E-48	4.7E-14
rs10401969	19	19407718	T	C	intronic	SUGP1	.	.	.	3.0E-48	5.4E-14
rs8107974	19	19388500	A	T	intronic	SUGP1	.	.	.	1.5E-47	1.4E-13
rs756350040	19	19370340	TGACA	T	.	.	.	.	.	2.5E-47	1.3E-13
rs2294922	22	44379565	G	C	intronic	SAMM50	.	.	.	7.6E-47	3.8E-08
19:19432290_AG_A	19	19432290	AG	A	.	.	.	.	.	8.3E-47	2.0E-13
rs73001065	19	19460541	G	C	intronic	MAU2	.	.	.	5.0E-46	4.7E-13
rs150268548	19	19494483	G	A	intergenic	MAU2;GATAD2A	dist=24920;dist=2169	.	.	5.9E-46	1.1E-12
rs739846	19	19419071	G	A	intronic	SUGP1	.	.	.	4.0E-44	5.6E-14
rs58489806	19	19456917	C	T	intronic	MAU2	.	.	.	4.3E-43	5.4E-15
rs12485100	22	44325516	G	T	intronic	PNPLA3	.	.	.	1.1E-42	5.2E-11
rs12484809	22	44325631	C	T	intronic	PNPLA3	.	.	.	1.1E-42	5.4E-11
rs12484801	22	44325565	C	T	intronic	PNPLA3	.	.	.	1.1E-42	5.2E-11
rs9625962	22	44326272	T	C	intronic	PNPLA3	.	.	.	2.3E-42	6.0E-11
rs12483959	22	44325996	G	A	intronic	PNPLA3	.	.	.	2.3E-42	5.6E-11
rs11090617	22	44326700	C	T	intronic	PNPLA3	.	.	.	2.4E-42	5.7E-11
rs72999033	19	19366632	C	T	UTR3	HAPLN4	NM_023002:c.*1994G>A	.	.	3.1E-42	1.6E-11
rs36038527	22	44332888	T	TC	intronic	PNPLA3	.	.	.	3.4E-42	5.0E-11
rs73176497	22	44336957	G	A	intronic	PNPLA3	.	.	.	3.4E-42	4.5E-11
rs2281135	22	44332570	G	A	intronic	PNPLA3	.	.	.	3.4E-42	3.4E-11
rs4823179	22	44341193	T	C	intronic	PNPLA3	.	.	.	3.7E-42	5.2E-11
rs34879941	22	44332878	C	T	intronic	PNPLA3	.	.	.	3.8E-42	3.9E-11
rs16991175	22	44335331	T	C	intronic	PNPLA3	.	.	.	4.0E-42	5.3E-11
rs35621602	22	44335406	C	A	intronic	PNPLA3	.	.	.	4.0E-42	5.3E-11
rs34352134	22	44335416	C	T	intronic	PNPLA3	.	.	.	4.0E-42	5.3E-11
rs34376930	22	44335453	G	T	intronic	PNPLA3	.	.	.	4.0E-42	5.3E-11
rs4823177	22	44334486	T	C	intronic	PNPLA3	.	.	.	4.0E-42	4.9E-11
rs4823178	22	44334529	T	C	intronic	PNPLA3	.	.	.	4.0E-42	4.9E-11
rs1010023	22	44336098	T	C	intronic	PNPLA3	.	.	.	4.0E-42	5.3E-11
rs1010022	22	44336310	A	G	intronic	PNPLA3	.	.	.	4.0E-42	5.4E-11
rs8142145	22	44336496	T	C	intronic	PNPLA3	.	.	.	4.0E-42	5.3E-11
rs2073081	22	44335744	T	C	intronic	PNPLA3	.	.	.	4.1E-42	5.5E-11
rs4823176	22	44334476	T	C	intronic	PNPLA3	.	.	.	4.1E-42	4.9E-11
rs2072906	22	44333172	A	G	intronic	PNPLA3	.	.	.	4.1E-42	4.2E-11
rs2072905	22	44333479	C	G	intronic	PNPLA3	.	.	.	4.1E-42	4.4E-11
rs2896019	22	44333694	T	G	intronic	PNPLA3	.	.	.	4.1E-42	4.6E-11
rs2401512	22	44333945	C	G	intronic	PNPLA3	.	.	.	4.1E-42	4.7E-11
rs2896020	22	44333968	T	C	intronic	PNPLA3	.	.	.	4.1E-42	4.7E-11
rs56255430	19	19477877	A	C	intergenic	MAU2;GATAD2A	dist=8314;dist=18775	.	.	4.3E-42	2.7E-12
rs13056555	22	44339526	C	G	intronic	PNPLA3	.	.	.	5.8E-42	6.8E-11
rs36069781	22	44340086	C	T	intronic	PNPLA3	.	.	.	5.8E-42	6.9E-11
rs2294916	22	44340922	T	G	intronic	PNPLA3	.	.	.	6.1E-42	5.2E-11
rs4823180	22	44341298	G	A	intronic	PNPLA3	.	.	.	6.1E-42	5.3E-11
rs4823181	22	44341606	T	C	intronic	PNPLA3	.	.	.	6.1E-42	5.4E-11
rs1883349	22	44331943	G	A	intronic	PNPLA3	.	.	.	6.6E-42	3.4E-11
rs926633	22	44337533	G	A	intronic	PNPLA3	.	.	.	7.5E-42	6.7E-11
rs1997693	22	44331513	C	G	intronic	PNPLA3	.	.	.	8.9E-42	1.9E-11
rs17217098	19	19702384	G	A	intronic	PBX4	.	.	.	1.2E-41	5.6E-12
rs2008451	22	44342969	T	C	UTR3	PNPLA3	NM_025225:c.*707T>C	.	.	1.6E-41	5.9E-11
rs1810508	22	44343151	A	G	UTR3	PNPLA3	NM_025225:c.*889A>G	.	.	1.6E-41	6.0E-11
rs2281293	22	44334842	T	C	intronic	PNPLA3	.	.	.	1.8E-41	3.1E-11
rs2076207	22	44333370	A	G	intronic	PNPLA3	.	.	.	1.9E-41	2.7E-11
rs2072907	22	44332653	C	G	intronic	PNPLA3	.	.	.	2.0E-41	2.2E-11
rs2281138	22	44332477	T	C	intronic	PNPLA3	.	.	.	2.0E-41	2.3E-11

Table continues on next page.

ID	Chr	POS	REF	ALT	Func.refGene	Gene.refGene	GeneDetail.refGene	ExonicFunc.refGene	AAChange.refGene	P (current study)	P (Sun et al)
rs13054885	22	44345771	G	A	intergenic	PNPLA3;SAMM50	dist=2309;dist=5551	.	.	2.1E-41	6.3E-11
rs13056638	22	44331778	C	G	intronic	PNPLA3	.	.	.	2.1E-41	2.0E-11
rs2281137	22	44332493	T	C	intronic	PNPLA3	.	.	.	2.1E-41	2.4E-11
rs1883348	22	44331815	C	G	intronic	PNPLA3	.	.	.	2.2E-41	2.1E-11
rs13055900	22	44341666	A	G	intronic	PNPLA3	.	.	.	2.8E-41	5.9E-11
rs13055874	22	44341672	T	C	intronic	PNPLA3	.	.	.	2.8E-41	6.0E-11
rs2294433	22	44329275	G	A	intronic	PNPLA3	.	.	.	5.0E-41	6.4E-11
rs1977080	22	44330031	C	T	intronic	PNPLA3	.	.	.	6.7E-41	5.2E-11
rs4823173	22	44328730	G	A	intronic	PNPLA3	.	.	.	8.2E-41	5.9E-11
rs2076211	22	44329078	C	T	intronic	PNPLA3	.	.	.	8.2E-41	6.1E-11
rs16991158	22	44327179	G	A	intronic	PNPLA3	.	.	.	9.6E-41	5.8E-11
rs36055245	22	44327192	A	G	intronic	PNPLA3	.	.	.	1.0E-40	5.8E-11
rs73004967	19	19717056	A	G	intronic	PBX4	.	.	.	1.0E-40	6.5E-12
rs1977081	22	44330128	T	C	intronic	PNPLA3	.	.	.	2.3E-40	2.7E-11
19:19699398_GA_G	19	19699398	GA	G	.	.	.	.	.	3.1E-40	6.5E-12
rs12484700	22	44327273	A	G	intronic	PNPLA3	.	.	.	3.8E-40	3.3E-11
rs2092501	22	44347251	G	A	intergenic	PNPLA3;SAMM50	dist=3789;dist=4071	.	.	1.0E-39	5.6E-10
rs34912062	22	44348446	G	T	intergenic	PNPLA3;SAMM50	dist=4984;dist=2876	.	.	1.6E-39	8.7E-10
rs1474745	22	44349236	T	C	intergenic	PNPLA3;SAMM50	dist=5774;dist=2086	.	.	4.4E-39	8.2E-10
rs56373884	22	44356468	G	A	intronic	SAMM50	.	.	.	6.5E-39	7.0E-10
rs3794991	19	19610596	C	T	intronic	GATAD2A	.	.	.	1.1E-38	2.3E-12
rs73002956	19	19578743	A	G	intronic	GATAD2A	.	.	.	2.2E-38	3.9E-12
rs2294921	22	44361842	C	T	intronic	SAMM50	.	.	.	2.4E-38	1.1E-09
rs12484795	22	44343626	A	C	downstream	PNPLA3	dist=164	.	.	3.3E-38	1.6E-10
rs3761472	22	44368122	A	G	exonic	SAMM50	.	nonsynonymous SNV	SAMM50:NM_015380:exon5:c.A329G:p.D110G	3.6E-38	1.7E-09
rs17216525	19	19662220	C	T	intergenic	CILP2;PBX4	dist=4752;dist=10302	.	.	4.3E-38	3.4E-10
rs16996148	19	19658472	G	T	intergenic	CILP2;PBX4	dist=1004;dist=14050	.	.	5.4E-38	1.2E-10
rs143988316	19	19667254	C	T	intergenic	CILP2;PBX4	dist=9786;dist=5268	.	.	7.6E-38	9.3E-11
rs17216588	19	19664077	C	T	intergenic	CILP2;PBX4	dist=6609;dist=8445	.	.	3.7E-37	1.3E-10
rs150824230	19	19670610	G	A	intergenic	CILP2;PBX4	dist=13142;dist=1912	.	.	9.9E-37	2.5E-11
rs73004926	19	19671266	C	T	intergenic	CILP2;PBX4	dist=13798;dist=1256	.	.	1.0E-36	2.1E-11
rs73004933	19	19675696	C	T	intronic	PBX4	.	.	.	1.1E-36	2.2E-11
rs141756246	19	19685470	G	GT	intronic	PBX4	.	.	.	3.1E-36	1.9E-11
rs73004962	19	19713069	A	T	intronic	PBX4	.	.	.	8.5E-36	3.1E-11
rs2228603	19	19329924	C	T	exonic	NCAN	.	nonsynonymous SNV	NCAN:NM_004386:exon3:c.C274T:p.P92S	9.1E-36	2.9E-12
rs12610185	19	19721722	G	A	intronic	PBX4	.	.	.	1.5E-35	4.6E-11
rs12610191	19	19721976	C	T	intronic	PBX4	.	.	.	1.5E-35	4.6E-11
rs73004951	19	19695228	C	T	intronic	PBX4	.	.	.	1.7E-35	2.0E-11
rs58847337	19	19726022	G	A	intronic	PBX4	.	.	.	2.1E-35	4.6E-11
rs57504626	19	19720399	C	T	intronic	PBX4	.	.	.	2.1E-35	5.0E-11
rs16996185	19	19720788	T	G	intronic	PBX4	.	.	.	2.1E-35	5.0E-11
rs10500212	19	19723215	C	T	intronic	PBX4	.	.	.	2.1E-35	4.8E-11
rs73004966	19	19716558	C	T	intronic	PBX4	.	.	.	2.2E-35	3.7E-11
rs12608729	19	19700552	C	T	intronic	PBX4	.	.	.	2.3E-35	2.9E-11
rs73004959	19	19711139	C	T	intronic	PBX4	.	.	.	2.4E-35	2.3E-11
rs73004975	19	19727152	A	G	intronic	PBX4	.	.	.	4.2E-35	4.2E-11
rs2304128	19	19746151	G	T	intronic	GMIP	.	.	.	2.7E-31	7.1E-10
rs57962361	19	19425025	C	T	intronic	SUGP1	.	.	.	1.1E-26	3.4E-11
rs111234557	19	19436229	C	G	intronic	MAU2	.	.	.	1.6E-26	3.7E-11
rs11668104	19	19426181	G	A	intronic	SUGP1	.	.	.	2.8E-26	4.8E-11
rs11672355	19	19462702	G	C	intronic	MAU2	.	.	.	3.1E-26	6.0E-11
rs11411903	19	19440064	T	TA	intronic	MAU2	.	.	.	3.8E-26	5.5E-11
19:19756073_AGCC_A	19	19756073	AGCC	A	.	.	.	.	.	3.1E-25	3.8E-09
19:19450254_CA_C	19	19450254	CA	C	.	.	.	.	.	1.3E-23	3.7E-11
rs56408111	19	19793545	T	C	UTR3	ZNF101	NM_001300949:c.*2436T>C;NM_033204:c.*2436T>C	.	.	1.8E-23	1.0E-10
rs58434384	19	19786099	A	G	intronic	ZNF101	.	.	.	4.0E-23	1.1E-10
rs2304130	19	19789528	A	G	intronic	ZNF101	.	.	.	5.1E-23	1.1E-10
rs12052117	19	19485105	C	T	intergenic	MAU2;GATAD2A	dist=15542;dist=11547	.	.	1.8E-22	3.5E-10
rs73002960	19	19582992	C	T	intronic	GATAD2A	.	.	.	3.4E-22	2.4E-10
rs28720066	19	19572220	G	T	intronic	GATAD2A	.	.	.	4.2E-22	2.7E-10

Table continues on next page.

ID	Chr	POS	REF	ALT	Func.refGene	Gene.refGene	GeneDetail.refGene	ExonicFunc.refGene	AAChange.refGene	P (current study)	P (Sun et al)
rs2285626	19	19467545	C	T	UTR3	MAU2	NM_015329:c.*954C>T	.	.	4.9E-22	1.4E-10
rs59148799	19	19484008	A	G	intergenic	MAU2;GATAD2A	dist=14445;dist=12644	.	.	5.0E-22	3.4E-10
rs56273306	19	19621004	T	C	intergenic	GATAD2A;TSSK6	dist=1263;dist=4024	.	.	5.2E-22	2.0E-10
rs56241616	19	19506092	C	T	intronic	GATAD2A	.	.	.	5.6E-22	4.4E-10
rs8182472	19	19539891	T	C	intronic	GATAD2A	.	.	.	5.6E-22	2.3E-10
rs10408875	19	19503573	T	C	intronic	GATAD2A	.	.	.	5.7E-22	4.4E-10
rs10408596	19	19512657	A	T	intronic	GATAD2A	.	.	.	5.7E-22	4.4E-10
rs10415849	19	19505087	C	T	intronic	GATAD2A	.	.	.	6.3E-22	4.3E-10
rs34324111	19	19513568	T	G	intronic	GATAD2A	.	.	.	1.0E-21	3.1E-10
rs35629458	19	19513572	T	G	intronic	GATAD2A	.	.	.	1.0E-21	3.1E-10
rs113460678	19	19513580	T	G	intronic	GATAD2A	.	.	.	1.0E-21	3.1E-10
rs56397647	19	19642795	C	T	intronic	YJEFN3	.	.	.	2.7E-21	9.3E-10
rs11668386	19	19531910	A	G	intronic	GATAD2A	.	.	.	8.0E-20	1.2E-09
rs10424702	19	19508013	A	G	intronic	GATAD2A	.	.	.	1.6E-19	1.5E-09
rs79954596	19	19548643	T	G	intronic	GATAD2A	.	.	.	1.9E-19	1.3E-09
rs188552254	19	19517169	A	G	intronic	GATAD2A	.	.	.	2.0E-19	1.4E-09
rs113365218	19	19621197	G	A	intergenic	GATAD2A;TSSK6	dist=1456;dist=3831	.	.	2.1E-19	2.9E-10
rs57009615	19	19613622	A	G	intronic	GATAD2A	.	.	.	1.3E-18	5.9E-10

Table S14. New associations at the genome-wide level found in our CC-GWAS compared to the GWAS from Sun et al.

ID	Chr	POS	REF	ALT	Func.refGene	Gene.refGene	GeneDetail.refGene	ExonicFunc.refGene	AAChange.refGene	P (current study)	P (Sun et al)
rs4823109	22	44381482	C	T	intronic	SAMM50	.	.	.	4.47E-16	1.70E-07
rs4823108	22	44381340	T	C	intronic	SAMM50	.	.	.	5.04E-16	1.60E-07
rs2235776	22	44377999	C	T	intronic	SAMM50	.	.	.	5.21E-16	1.70E-07
rs4823183	22	44378672	C	A	intronic	SAMM50	.	.	.	5.22E-16	1.50E-07
rs2235777	22	44378809	C	T	intronic	SAMM50	.	.	.	5.22E-16	1.50E-07
rs71313378	22	44380170	G	GCTTC	intronic	SAMM50	.	.	.	5.64E-16	1.90E-07
rs12167845	22	44380767	T	C	intronic	SAMM50	.	.	.	6.40E-16	1.70E-07
rs61473277	22	44371406	A	G	intronic	SAMM50	.	.	.	6.64E-16	1.70E-07
rs2294923	22	44379740	C	A	intronic	SAMM50	.	.	.	6.91E-16	1.70E-07
rs9626079	22	44380009	A	G	intronic	SAMM50	.	.	.	6.91E-16	1.70E-07
rs3761077	19	19325963	G	T	intronic	NCAN	.	.	.	9.70E-14	1.00E-05
rs12979148	19	19406869	T	C	intronic	SUGP1	.	.	.	1.52E-13	1.90E-06
rs2238675	19	19336608	C	T	intronic	NCAN	.	.	.	3.32E-13	2.60E-05
rs2240117	19	19418916	C	T	intronic	SUGP1	.	.	.	8.54E-13	2.20E-06
rs12165526	22	44361713	T	A	intronic	SAMM50	.	.	.	1.11E-12	5.30E-03
rs35431065	19	19393677	A	G	intronic	SUGP1	.	.	.	3.52E-11	9.60E-05
rs34755166	19	19665581	G	A	intergenic	CILP2;PBX4	dist=8113;dist=6941	.	.	9.66E-11	1.90E-05
rs17216693	19	19666574	T	C	intergenic	CILP2;PBX4	dist=9106;dist=5948	.	.	9.81E-11	1.20E-05
rs9306471	22	44328075	A	G	intronic	PNPLA3	.	.	.	2.83E-10	3.00E-02
rs4823182	22	44377442	A	G	intronic	SAMM50	.	.	.	3.01E-10	2.10E-03
19:19668338_GC_G	19	19668338	GC	G	.	.	.	.	.	3.27E-10	1.30E-05
rs67450864	22	44376335	C	T	intronic	SAMM50	.	.	.	3.39E-10	2.00E-03
rs9626056	22	44327075	C	T	intronic	PNPLA3	.	.	.	3.44E-10	3.00E-02
rs111393709	22	44329719	C	T	intronic	PNPLA3	.	.	.	3.60E-10	3.80E-02
rs80341032	19	19207229	G	A	intronic	SLC25A42	.	.	.	9.55E-10	4.00E-06
rs2281298	22	44391234	G	A	intronic	SAMM50	.	.	.	9.71E-10	5.90E-05
rs10656207	22	44387932	C	CTA	intronic	SAMM50	.	.	.	1.08E-09	2.90E-03
rs3827385	22	44388817	T	C	intronic	SAMM50	.	.	.	1.25E-09	2.50E-05
rs2143571	22	44391686	G	A	intronic	SAMM50	.	.	.	1.27E-09	5.20E-05
rs2401514	22	44394019	T	A	intergenic	SAMM50;PARVB	dist=1610;dist=1072	.	.	1.30E-09	5.80E-05
rs2294927	22	44382684	T	C	intronic	SAMM50	.	.	.	1.31E-09	3.20E-03
rs2073079	22	44385594	A	G	intronic	SAMM50	.	.	.	1.32E-09	6.60E-05
rs2073080	22	44394402	C	T	upstream	PARVB	dist=689	.	.	1.52E-09	6.60E-05
rs6006602	22	44383400	C	T	intronic	SAMM50	.	.	.	1.78E-09	3.70E-03
rs6006468	22	44383432	G	C	intronic	SAMM50	.	.	.	1.78E-09	3.70E-03
rs73008942	19	19155672	G	A	intronic	ARMC6	.	.	.	1.93E-09	1.20E-06
rs6006469	22	44383617	C	G	intronic	SAMM50	.	.	.	2.08E-09	3.50E-03
rs55768287	22	44324558	C	T	intronic	PNPLA3	.	.	.	2.20E-09	2.00E-02
rs2235778	22	44389514	T	C	intronic	SAMM50	.	.	.	2.23E-09	4.10E-03
22:44335670_TGG_T	22	44335670	TGG	T	.	.	.	.	.	2.30E-09	3.60E-07
rs3788604	22	44388417	A	G	intronic	SAMM50	.	.	.	2.52E-09	4.40E-03
rs1007863	22	44395451	T	C	exonic	PARVB	.	nonsynonymous SNV	PARVB:NM_001003828:exon2:c.T109C:p.W37R	2.54E-09	4.50E-03
rs1986095	22	44387108	A	G	intronic	SAMM50	.	.	.	2.65E-09	3.80E-03
rs8105094	19	19374061	C	T	upstream	HAPLN4	dist=448	.	.	2.71E-09	4.50E-06
rs8105984	19	19374068	T	C	upstream	HAPLN4	dist=455	.	.	2.71E-09	4.50E-06
rs6006473	22	44393075	C	T	downstream	SAMM50	dist=666	.	.	2.92E-09	3.50E-03
rs2281292	22	44395389	A	C	intronic	PARVB	.	.	.	3.89E-09	3.60E-03
rs9625964	22	44337610	G	A	intronic	PNPLA3	.	.	.	4.37E-09	6.70E-02
rs9625966	22	44338105	C	T	intronic	PNPLA3	.	.	.	4.37E-09	6.90E-02
rs9625965	22	44338049	T	C	intronic	PNPLA3	.	.	.	4.49E-09	7.50E-02
rs73006914	19	19110422	C	T	intronic	SUGP2	.	.	.	4.97E-09	1.00E-06
rs9626057	22	44339791	C	G	intronic	PNPLA3	.	.	.	5.20E-09	7.40E-02
rs117772800	22	44339055	A	G	intronic	PNPLA3	.	.	.	5.67E-09	7.50E-02
rs12166587	22	44383070	T	C	intronic	SAMM50	.	.	.	5.98E-09	1.10E-01
rs9625970	22	44383502	T	C	intronic	SAMM50	.	.	.	6.03E-09	1.10E-01
rs112902984	22	44385583	T	C	intronic	SAMM50	.	.	.	6.25E-09	1.10E-01
rs117472787	22	44387298	C	T	intronic	SAMM50	.	.	.	6.25E-09	1.10E-01
rs3810444	19	19103986	T	A	UTR3	SUGP2	NM_001321699:c.*564A>T;NM_001352071:c.*564A>T;NM_001017392:c.*564A>T	.	.	6.51E-09	3.90E-06
rs41278873	22	44342691	T	C	UTR3	PNPLA3	NM_025225:c.*429T>C	.	.	7.32E-09	1.20E-01

Table continues on next page.

ID	Chr	POS	REF	ALT	Func.refGene	Gene.refGene	GeneDetail.refGene	ExonicFunc.refGene	AAChange.refGene	P (current study)	P (Sun et al)
rs8141950	22	44393476	C	T	intergenic	SAMM50;PARVB	dist=1067;dist=1615	.	.	1.04E-08	1.10E-01
rs58833986	19	19434042	T	TCACCA	intronic	MAU2	.	.	.	1.22E-08	4.70E-06
rs8141994	22	44397144	A	G	intronic	PARVB	.	.	.	1.67E-08	1.10E-01
rs2074301	19	19381715	G	A	intronic	TM6SF2	.	.	.	1.67E-08	8.50E-06
rs2074300	19	19380996	G	T	exonic	TM6SF2	.	synonymous SNV	TM6SF2:NM_001001524:exon4:c.C387A:p.G129G	1.80E-08	8.90E-06
rs738494	22	44370439	A	C	intronic	SAMM50	.	.	.	1.86E-08	1.00E-01
rs9626071	22	44359938	C	A	intronic	SAMM50	.	.	.	2.00E-08	1.00E-01
rs28421169	22	44356562	T	C	intronic	SAMM50	.	.	.	2.15E-08	1.20E-01
rs28754570	22	44358819	G	C	intronic	SAMM50	.	.	.	2.18E-08	1.00E-01
rs12170274	22	44359729	C	T	intronic	SAMM50	.	.	.	2.20E-08	1.00E-01
rs12168138	22	44359651	T	C	intronic	SAMM50	.	.	.	2.22E-08	1.00E-01
rs2285628	19	19467996	T	A	UTR3	MAU2	NM_015329:c.*1405T>A	.	.	2.44E-08	3.50E-06
rs563530889	19	19374546	A	AAG	upstream;downstream	HAPLN4;TM6SF2	dist=933;dist=630	.	.	2.61E-08	8.00E-06
rs10419672	19	19471241	T	C	intergenic	MAU2;GATAD2A	dist=1678;dist=25411	.	.	3.02E-08	4.80E-06
rs9626058	22	44343352	A	G	UTR3	PNPLA3	NM_025225:c.*1090A>G	.	.	3.33E-08	7.60E-02
rs12459676	19	19425141	A	T	intronic	SUGP1	.	.	.	3.49E-08	5.80E-06
19:19393106_CAAGA_C	19	19393106	CAAGA	C	.	.	.	.	.	3.55E-08	1.50E-05
rs2023883	19	19405480	G	A	intronic	SUGP1	.	.	.	3.66E-08	5.20E-06
rs2294917	22	44341986	T	C	intronic	PNPLA3	.	.	.	3.74E-08	6.50E-02
rs11666553	19	19407171	C	A	intronic	SUGP1	.	.	.	3.91E-08	5.50E-06
rs9626076	22	44368584	G	A	intronic	SAMM50	.	.	.	3.93E-08	9.00E-02
rs9626075	22	44366874	G	A	intronic	SAMM50	.	.	.	3.95E-08	8.90E-02
rs9626061	22	44344872	C	T	intergenic	PNPLA3;SAMM50	dist=1410;dist=6450	.	.	4.01E-08	6.00E-02
rs9626074	22	44363736	C	T	intronic	SAMM50	.	.	.	4.08E-08	8.80E-02
rs12330016	22	44369927	C	T	intronic	SAMM50	.	.	.	4.13E-08	9.30E-02
rs117130990	22	44371030	G	A	intronic	SAMM50	.	.	.	4.13E-08	9.40E-02
rs9626073	22	44362178	G	A	intronic	SAMM50	.	.	.	4.13E-08	9.10E-02
rs73434655	22	44370955	A	G	intronic	SAMM50	.	.	.	4.20E-08	9.40E-02
rs12168183	22	44366135	G	A	intronic	SAMM50	.	.	.	4.20E-08	9.00E-02
19:19422152_CA_C	19	19422152	CA	C	.	.	.	.	.	4.27E-08	6.80E-06
rs9626068	22	44358360	C	G	intronic	SAMM50	.	.	.	4.48E-08	9.40E-02
rs10403731	19	19469296	G	A	UTR3	MAU2	NM_015329:c.*2705G>A	.	.	4.49E-08	5.20E-06
rs12976025	19	19429220	C	T	intronic	SUGP1	.	.	.	4.54E-08	5.10E-06
rs4808194	19	19435680	T	G	intronic	MAU2	.	.	.	4.54E-08	5.10E-06
rs12983137	19	19419810	G	A	intronic	SUGP1	.	.	.	4.73E-08	5.50E-06
rs1859287	19	19422187	T	C	intronic	SUGP1	.	.	.	4.73E-08	5.50E-06
rs7259434	19	19428805	A	T	intronic	SUGP1	.	.	.	4.73E-08	5.50E-06
rs10402661	19	19432959	A	G	intronic	MAU2	.	.	.	4.73E-08	5.50E-06
rs9626067	22	44358030	C	T	intronic	SAMM50	.	.	.	4.77E-08	9.40E-02
rs9626066	22	44357940	T	A	intronic	SAMM50	.	.	.	4.84E-08	9.50E-02
rs9626065	22	44357928	A	C	intronic	SAMM50	.	.	.	4.90E-08	9.50E-02
rs757000	19	19448301	A	G	intronic	MAU2	.	.	.	4.97E-08	5.20E-06
rs757001	19	19448808	G	A	intronic	MAU2	.	.	.	4.97E-08	5.20E-06
rs2301668	19	19452249	G	A	intronic	MAU2	.	.	.	4.97E-08	5.20E-06
rs9626078	22	44373947	G	A	intronic	SAMM50	.	.	.	5.00E-08	9.30E-02

Table S15. New variants associations at the genome-wide level found in our QT-GWAS compared to the GWAS from Sun et al.

ID	Chr	POS	REF	ALT	Func.refGene	Gene.refGene	GeneDetail.refGene	ExonicFunc.refGene	AAChange.refGene	P	p_value
rs4823109	22	44381482	C	T	intronic	SAMM50	.	.	.	2.02E-32	1.70E-07
rs4823183	22	44378672	C	A	intronic	SAMM50	.	.	.	2.14E-32	1.50E-07
rs2235777	22	44378809	C	T	intronic	SAMM50	.	.	.	2.14E-32	1.50E-07
rs4823108	22	44381340	T	C	intronic	SAMM50	.	.	.	2.30E-32	1.60E-07
rs61473277	22	44371406	A	G	intronic	SAMM50	.	.	.	2.38E-32	1.70E-07
rs2235776	22	44377999	C	T	intronic	SAMM50	.	.	.	2.75E-32	1.70E-07
rs71313378	22	44380170	G	GCTTC	intronic	SAMM50	.	.	.	2.79E-32	1.90E-07
rs12167845	22	44380767	T	C	intronic	SAMM50	.	.	.	2.88E-32	1.70E-07
rs2294923	22	44379740	C	A	intronic	SAMM50	.	.	.	3.29E-32	1.70E-07
rs9626079	22	44380009	A	G	intronic	SAMM50	.	.	.	3.29E-32	1.70E-07
rs12979148	19	19406869	T	C	intronic	SUGP1	.	.	.	4.22E-25	1.90E-06
rs2240117	19	19418916	C	T	intronic	SUGP1	.	.	.	1.32E-24	2.20E-06
rs3761077	19	19325963	G	T	intronic	NCAN	.	.	.	3.66E-22	1.00E-05
rs34755166	19	19665581	G	A	intergenic	CILP2;PBX4	dist=8113;dist=6941	.	.	1.22E-21	1.90E-05
rs17216693	19	19666574	T	C	intergenic	CILP2;PBX4	dist=9106;dist=5948	.	.	1.43E-21	1.20E-05
rs2281298	22	44391234	G	A	intronic	SAMM50	.	.	.	1.45E-21	5.90E-05
rs2143571	22	44391686	G	A	intronic	SAMM50	.	.	.	1.48E-21	5.20E-05
rs2401514	22	44394019	T	A	intergenic	SAMM50;PARVB	dist=1610;dist=1072	.	.	4.39E-21	5.80E-05
rs2073079	22	44385594	A	G	intronic	SAMM50	.	.	.	4.98E-21	6.60E-05
rs2073080	22	44394402	C	T	upstream	PARVB	dist=689	.	.	5.53E-21	6.60E-05
rs3827385	22	44388817	T	C	intronic	SAMM50	.	.	.	6.47E-21	2.50E-05
rs2238675	19	19336608	C	T	intronic	NCAN	.	.	.	2.44E-20	2.60E-05
rs35431065	19	19393677	A	G	intronic	SUGP1	.	.	.	8.93E-20	9.60E-05
19:19668338_GC_G	19	19668338	GC	G	.	.	.	.	.	2.03E-19	1.30E-05
rs67450864	22	44376335	C	T	intronic	SAMM50	.	.	.	3.85E-19	2.00E-03
rs4823182	22	44377442	A	G	intronic	SAMM50	.	.	.	4.08E-19	2.10E-03
rs12165526	22	44361713	T	A	intronic	SAMM50	.	.	.	1.44E-18	5.30E-03
rs10656207	22	44387932	C	CTA	intronic	SAMM50	.	.	.	2.11E-18	2.90E-03
22:44335670_TGG_T	22	44335670	TGG	T	.	.	.	.	.	2.93E-18	3.60E-07
rs1007863	22	44395451	T	C	exonic	PARVB	.	nonsynonymous SNV	PARVB:NM_001003828:exon2:c.T109C:p.W37R	3.54E-18	4.50E-03
rs6006602	22	44383400	C	T	intronic	SAMM50	.	.	.	5.53E-18	3.70E-03
rs6006468	22	44383432	G	C	intronic	SAMM50	.	.	.	5.53E-18	3.70E-03
rs2294927	22	44382684	T	C	intronic	SAMM50	.	.	.	5.80E-18	3.20E-03
rs2235778	22	44389514	T	C	intronic	SAMM50	.	.	.	6.03E-18	4.10E-03
rs6006473	22	44393075	C	T	downstream	SAMM50	dist=666	.	.	6.19E-18	3.50E-03
rs6006469	22	44383617	C	G	intronic	SAMM50	.	.	.	7.09E-18	3.50E-03
rs2281292	22	44395389	A	C	intronic	PARVB	.	.	.	7.22E-18	3.60E-03
rs3788604	22	44388417	A	G	intronic	SAMM50	.	.	.	8.25E-18	4.40E-03
rs1986095	22	44387108	A	G	intronic	SAMM50	.	.	.	8.64E-18	3.80E-03
rs16991236	22	44358997	A	G	intronic	SAMM50	.	.	.	1.50E-17	7.40E-04
rs1883350	22	44328043	T	C	intronic	PNPLA3	.	.	.	6.68E-17	3.30E-05
rs73008942	19	19155672	G	A	intronic	ARMC6	.	.	.	3.88E-15	1.20E-06
rs73006914	19	19110422	C	T	intronic	SUGP2	.	.	.	4.64E-15	1.00E-06
rs80341032	19	19207229	G	A	intronic	SLC25A42	.	.	.	1.08E-14	4.00E-06
rs58833986	19	19434042	T	TCACCA	intronic	MAU2	.	.	.	2.36E-14	4.70E-06
rs3810444	19	19103986	T	A	UTR3	SUGP2	NM_001321699:c.*564A>T;NM_001352071:c.*564A>T;NM_001017392:c.*564A>T	.	.	2.48E-14	3.90E-06
rs8105094	19	19374061	C	T	upstream	HAPLN4	dist=448	.	.	2.54E-14	4.50E-06
rs8105984	19	19374068	T	C	upstream	HAPLN4	dist=455	.	.	2.54E-14	4.50E-06
rs9626056	22	44327075	C	T	intronic	PNPLA3	.	.	.	4.23E-14	3.00E-02
rs9306471	22	44328075	A	G	intronic	PNPLA3	.	.	.	5.63E-14	3.00E-02
rs2023883	19	19405480	G	A	intronic	SUGP1	.	.	.	7.88E-14	5.20E-06
rs11666553	19	19407171	C	A	intronic	SUGP1	.	.	.	8.31E-14	5.50E-06
19:19422152_CA_C	19	19422152	CA	C	.	.	.	.	.	8.32E-14	6.80E-06
rs12459676	19	19425141	A	T	intronic	SUGP1	.	.	.	8.95E-14	5.80E-06
rs12981405	19	19651577	C	T	intronic	CILP2	.	.	.	9.06E-14	3.20E-05
rs2074301	19	19381715	G	A	intronic	TM6SF2	.	.	.	9.06E-14	8.50E-06
rs2285628	19	19467996	T	A	UTR3	MAU2	NM_015329:c.*1405T>A	.	.	1.06E-13	3.50E-06
rs775175628	19	19560756	TATCTTATATTAA	T	.	.	.	.	.	1.09E-13	8.80E-06
rs2074300	19	19380996	G	T	exonic	TM6SF2	.	synonymous SNV	TM6SF2:NM_001001524:exon4:c.C387A:p.G129G	1.13E-13	8.90E-06
rs10419672	19	19471241	T	C	intergenic	MAU2;GATAD2A	dist=1678;dist=25411	.	.	1.32E-13	4.80E-06
rs111393709	22	44329719	C	T	intronic	PNPLA3	.	.	.	1.32E-13	3.80E-02
rs12983137	19	19419810	G	A	intronic	SUGP1	.	.	.	1.38E-13	5.50E-06
rs1859287	19	19422187	T	C	intronic	SUGP1	.	.	.	1.38E-13	5.50E-06
rs7259434	19	19428805	A	T	intronic	SUGP1	.	.	.	1.38E-13	5.50E-06
rs10402661	19	19432959	A	G	intronic	MAU2	.	.	.	1.38E-13	5.50E-06
rs12976025	19	19429220	C	T	intronic	SUGP1	.	.	.	1.39E-13	5.10E-06
rs4808194	19	19435680	T	G	intronic	MAU2	.	.	.	1.39E-13	5.10E-06
rs10403731	19	19469296	G	A	UTR3	MAU2	NM_015329:c.*2705G>A	.	.	1.44E-13	5.20E-06
rs11085261	19	19462606	G	A	intronic	MAU2	.	.	.	1.53E-13	6.30E-06
rs563530889	19	19374546	A	AAG	upstream;downstream	HAPLN4;TM6SF2	dist=933;dist=630	.	.	1.54E-13	8.00E-06
rs11085259	19	19445856	C	T	intronic	MAU2	.	.	.	1.61E-13	5.40E-06
rs8108647	19	19427623	A	G	intronic	SUGP1	.	.	.	1.62E-13	4.10E-06
rs757000	19	19448301	A	G	intronic	MAU2	.	.	.	1.62E-13	5.20E-06
rs757001	19	19448808	G	A	intronic	MAU2	.	.	.	1.62E-13	5.20E-06
rs2301668	19	19452249	G	A	intronic	MAU2	.	.	.	1.62E-13	5.20E-06
rs12982276	19	19459800	T	C	intronic	MAU2	.	.	.	1.75E-13	5.10E-06
rs756264087	19	19423083	CAAT	C	.	.	.	.	.	1.83E-13	5.80E-06
rs10421505	19	19459554	C	T	intronic	MAU2	.	.	.	1.86E-13	4.60E-06
rs7258508	19	19475469	T	C	intergenic	MAU2;GATAD2A	dist=5906;dist=21183	.	.	2.06E-13	1.40E-05
rs968525	19	19459215	C	T	intronic	MAU2	.	.	.	2.24E-13	5.30E-06
rs55768287	22	44324558	C	T	intronic	PNPLA3	.	.	.	2.27E-13	2.00E-02
rs2294917	22	44341986	T	C	intronic	PNPLA3	.	.	.	2.58E-13	6.50E-02
19:19431420_GT_G	19	19431420	GT	G	.	.	.	.	.	2.66E-13	5.40E-06
rs9304960	19	19465529	G	A	intronic	MAU2	.	.	.	3.60E-13	7.80E-06
rs2301671	19	19466269	C	T	intronic	MAU2	.	.	.	3.79E-13	8.50E-06
19:19393106_CAAGA_C	19	19393106	CAAGA	C	.	.	.	.	.	4.01E-13	1.50E-05
rs4808196	19	19476520	G	A	intergenic	MAU2;GATAD2A	dist=6957;dist=20132	.	.	5.42E-13	1.20E-05
rs12166587	22	44383070	T	C	intronic	SAMM50	.	.	.	6.94E-13	1.10E-01
rs9625970	22	44383502	T	C	intronic	SAMM50	.	.	.	7.01E-13	1.10E-01

Table continues on next page.

ID	Chr	POS	REF	ALT	Func.refGene	Gene.refGene	GeneDetail.refGene	ExonicFunc.refGene	AAChange.refGene	P	p_value
rs112902984	22	44385583	T	C	intronic	SAMM50	.	.	.	7.31E-13	1.10E-01
rs117472787	22	44387298	C	T	intronic	SAMM50	.	.	.	7.31E-13	1.10E-01
rs738491	22	44354111	C	T	intronic	SAMM50	.	.	.	8.22E-13	4.90E-04
rs10418051	19	19397789	C	T	intronic	SUGP1	.	.	.	8.93E-13	9.70E-07
rs11404084	19	19579241	T	TA	intronic	GATAD2A	.	.	.	9.99E-13	7.50E-06
rs8141950	22	44393476	C	T	intergenic	SAMM50;PARVB	dist=1067;dist=1615	.	.	1.16E-12	1.10E-01
rs1465695	19	19588546	A	C	intronic	GATAD2A	.	.	.	1.37E-12	8.60E-06
rs10404728	19	19595014	C	T	intronic	GATAD2A	.	.	.	1.37E-12	8.60E-06
rs751858	19	19602821	G	C	intronic	GATAD2A	.	.	.	1.38E-12	6.30E-06
rs17288409	19	19504167	T	C	intronic	GATAD2A	.	.	.	1.39E-12	1.10E-05
rs10401193	19	19591066	A	G	intronic	GATAD2A	.	.	.	1.39E-12	9.30E-06
rs9625965	22	44338049	T	C	intronic	PNPLA3	.	.	.	1.41E-12	7.50E-02
rs754255	19	19578890	T	C	intronic	GATAD2A	.	.	.	1.48E-12	8.80E-06
rs60003758	19	19557353	A	G	intronic	GATAD2A	.	.	.	1.50E-12	9.00E-06
rs9625966	22	44338105	C	T	intronic	PNPLA3	.	.	.	1.50E-12	6.90E-02
rs9625964	22	44337610	G	A	intronic	PNPLA3	.	.	.	1.52E-12	6.70E-02
rs12972397	19	19562349	G	A	intronic	GATAD2A	.	.	.	1.58E-12	9.00E-06
rs9626057	22	44339791	C	G	intronic	PNPLA3	.	.	.	1.83E-12	7.40E-02
rs60321073	19	19499598	A	G	intronic	GATAD2A	.	.	.	1.98E-12	1.20E-05
rs2099333	19	19605963	C	T	intronic	GATAD2A	.	.	.	2.04E-12	9.70E-06
rs56219234	22	44357894	G	T	intronic	SAMM50	.	.	.	2.10E-12	1.00E-03
rs61061000	19	19283268	C	T	intronic	BORCS8-MEF2B	.	.	.	2.17E-12	9.00E-06
rs12977524	19	19568244	A	G	intronic	GATAD2A	.	.	.	2.25E-12	9.70E-06
rs4808199	19	19545099	G	A	intronic	GATAD2A	.	.	.	2.27E-12	1.00E-05
rs117772800	22	44339055	A	G	intronic	PNPLA3	.	.	.	2.29E-12	7.50E-02
rs7250658	19	19571100	A	G	intronic	GATAD2A	.	.	.	2.36E-12	1.00E-05
rs6511036	19	19582651	A	G	intronic	GATAD2A	.	.	.	2.45E-12	1.00E-05
rs7252888	19	19628037	G	A	intronic	NDUFA13	.	.	.	2.48E-12	7.90E-06
rs2163805	19	19575945	G	A	intronic	GATAD2A	.	.	.	2.50E-12	1.00E-05
rs4808960	19	19574277	G	C	intronic	GATAD2A	.	.	.	2.54E-12	1.00E-05
rs2163804	19	19575965	G	A	intronic	GATAD2A	.	.	.	2.56E-12	1.00E-05
19:19510831_AT_A	19	19510831	AT	A	.	.	.	.	.	3.16E-12	1.90E-05
rs738494	22	44370439	A	C	intronic	SAMM50	.	.	.	4.62E-12	1.00E-01
rs9626061	22	44344872	C	T	intergenic	PNPLA3;SAMM50	dist=1410;dist=6450	.	.	4.95E-12	6.00E-02
rs9626071	22	44359938	C	A	intronic	SAMM50	.	.	.	5.57E-12	1.00E-01
rs8141994	22	44397144	A	G	intronic	PARVB	.	.	.	5.59E-12	1.10E-01
rs12168138	22	44359651	T	C	intronic	SAMM50	.	.	.	5.80E-12	1.00E-01
rs28754570	22	44358819	G	C	intronic	SAMM50	.	.	.	5.83E-12	1.00E-01
rs12170274	22	44359729	C	T	intronic	SAMM50	.	.	.	5.83E-12	1.00E-01
rs28421169	22	44356562	T	C	intronic	SAMM50	.	.	.	6.10E-12	1.20E-01
rs9626058	22	44343352	A	G	UTR3	PNPLA3	NM_025225:c.*1090A>G	.	.	6.43E-12	7.60E-02
rs6006599	22	44382004	C	A	intronic	SAMM50	.	.	.	7.42E-12	2.20E-03
rs9626076	22	44368584	G	A	intronic	SAMM50	.	.	.	8.15E-12	9.00E-02
rs12168183	22	44366135	G	A	intronic	SAMM50	.	.	.	8.25E-12	9.00E-02
rs9626075	22	44366874	G	A	intronic	SAMM50	.	.	.	8.52E-12	8.90E-02
rs28733632	22	44353447	C	T	intronic	SAMM50	.	.	.	8.59E-12	9.80E-02
rs117369516	22	44348284	C	T	intergenic	PNPLA3;SAMM50	dist=4822;dist=3038	.	.	8.68E-12	9.60E-02
rs9626064	22	44354598	C	T	intronic	SAMM50	.	.	.	8.93E-12	1.20E-01
rs9626074	22	44363736	C	T	intronic	SAMM50	.	.	.	9.01E-12	8.80E-02
rs12330016	22	44369927	C	T	intronic	SAMM50	.	.	.	9.15E-12	9.30E-02
rs117130990	22	44371030	G	A	intronic	SAMM50	.	.	.	9.15E-12	9.40E-02
rs9626073	22	44362178	G	A	intronic	SAMM50	.	.	.	9.17E-12	9.10E-02
rs73434655	22	44370955	A	G	intronic	SAMM50	.	.	.	9.23E-12	9.40E-02
rs12167852	22	44344011	A	G	downstream	PNPLA3	dist=549	.	.	9.59E-12	7.60E-02
rs41278873	22	44342691	T	C	UTR3	PNPLA3	NM_025225:c.*429T>C	.	.	1.03E-11	1.20E-01
rs9626078	22	44373947	G	A	intronic	SAMM50	.	.	.	1.04E-11	9.30E-02
rs28478453	19	19531175	C	G	intronic	GATAD2A	.	.	.	1.09E-11	1.50E-05
rs9626065	22	44357928	A	C	intronic	SAMM50	.	.	.	1.10E-11	9.50E-02
rs9626066	22	44357940	T	A	intronic	SAMM50	.	.	.	1.12E-11	9.50E-02
rs9626067	22	44358030	C	T	intronic	SAMM50	.	.	.	1.12E-11	9.40E-02
rs75439392	22	44377221	G	T	intronic	SAMM50	.	.	.	1.16E-11	9.30E-02
rs9626068	22	44358360	C	G	intronic	SAMM50	.	.	.	1.16E-11	9.40E-02
rs11912828	22	44348116	G	A	intergenic	PNPLA3;SAMM50	dist=4654;dist=3206	.	.	1.19E-11	1.00E-01
rs2294919	22	44342325	C	T	UTR3	PNPLA3	NM_025225:c.*63C>T	.	.	1.25E-11	1.30E-01
rs565756573	19	19461416	C	CA	intronic	MAU2	.	.	.	1.29E-11	1.20E-05
rs113954889	19	19294392	T	A	intronic	BORCS8;BORCS8-MEF2B	.	.	.	1.33E-11	1.10E-05
rs10402308	19	19657500	G	A	downstream	CILP2	dist=32	.	.	1.36E-11	3.50E-05
rs12170782	22	44347504	C	A	intergenic	PNPLA3;SAMM50	dist=4042;dist=3818	.	.	1.69E-11	8.80E-02
rs11669516	19	19532682	G	A	intronic	GATAD2A	.	.	.	1.95E-11	1.60E-05
rs139052	22	44327012	A	C	intronic	PNPLA3	.	.	.	2.10E-11	1.30E-01
rs34647936	19	19548239	T	G	intronic	GATAD2A	.	.	.	2.33E-11	1.70E-05
rs12983940	19	19516431	G	A	intronic	GATAD2A	.	.	.	2.35E-11	1.90E-05
rs12973258	19	19488718	T	C	intergenic	MAU2;GATAD2A	dist=19155;dist=7934	.	.	2.89E-11	2.20E-05
rs4808950	19	19518889	A	G	intronic	GATAD2A	.	.	.	3.69E-11	2.30E-05
rs8100140	19	19314526	G	A	upstream	NR2C2AP	dist=303	.	.	6.10E-11	1.50E-02
22:44381944_ATGGAGTCTTGCTC_A	22	44381944	ATGGAGTCTTGCTC	A	.	.	.	.	.	1.05E-10	1.20E-03
rs1474744	22	44349215	C	T	intergenic	PNPLA3;SAMM50	dist=5753;dist=2107	.	.	1.30E-10	4.40E-04
rs111901094	19	19513570	G	T	intronic	GATAD2A	.	.	.	1.36E-10	3.30E-05
rs2965185	19	19525792	T	C	intronic	GATAD2A	.	.	.	8.65E-10	7.80E-06
rs12484530	22	44409993	G	A	intronic	PARVB	.	.	.	1.61E-09	1.90E-04
rs60204587	19	54671421	G	A	intronic	TMC4	.	.	.	2.32E-09	7.70E-02
rs6006594	22	44365232	C	G	intronic	SAMM50	.	.	.	3.02E-09	4.30E-04
rs28451834	19	19260760	G	C	intronic	BORCS8-MEF2B;MEF2B	.	.	.	3.66E-09	5.50E-06
rs2073086	22	44372331	C	T	intronic	SAMM50	.	.	.	3.76E-09	1.50E-02
rs12609436	19	19743098	C	T	intronic	GMIP	.	.	.	3.93E-09	4.00E-04
rs4806498	19	54674742	C	T	intronic	TMC4	.	.	.	4.04E-09	3.70E-02
rs1474746	22	44350417	G	C	upstream	SAMM50	dist=905	.	.	4.41E-09	4.10E-04
rs873870	19	19738554	G	A	intronic	LPAR2	.	.	.	4.55E-09	6.80E-04

Table continues on next page.

ID	Chr	POS	REF	ALT	Func.refGene	Gene.refGene	GeneDetail.refGene	ExonicFunc.refGene	AAChange.refGene	P	p_value
rs139051	22	44324676	G	A	intronic	PNPLA3	.	.	.	4.56E-09	1.40E-05
rs3083314	22	44343239	CAA	C	.	.	.	.	.	6.53E-09	4.90E-04
rs2401513	22	44355569	C	T	intronic	SAMM50	.	.	.	7.18E-09	3.40E-01
rs2872878	19	19480521	C	G	intergenic	MAU2;GATAD2A	dist=10958;dist=16131	.	.	7.66E-09	3.40E-04
rs2073084	22	44372069	G	A	intronic	SAMM50	.	.	.	8.48E-09	1.90E-02
rs28550680	22	44345926	C	T	intergenic	PNPLA3;SAMM50	dist=2464;dist=5396	.	.	8.57E-09	2.70E-01
rs641738	19	54676763	C	T	exonic	TMC4	.	nonsynonymous SNV	TMC4:NM_001145303:exon1:c.G50A:p.G17E,TMC4:NM_144686:exon1:c.G50A:p.G17E	9.27E-09	8.10E-02
rs11704562	22	44346128	C	T	intergenic	PNPLA3;SAMM50	dist=2666;dist=5194	.	.	9.27E-09	2.70E-01
rs7289329	22	44346639	T	G	intergenic	PNPLA3;SAMM50	dist=3177;dist=4683	.	.	9.32E-09	3.00E-01
rs2073085	22	44372190	C	T	intronic	SAMM50	.	.	.	9.63E-09	1.80E-02
rs11705218	22	44354865	A	G	intronic	SAMM50	.	.	.	9.77E-09	3.60E-01
rs36010983	19	19564489	A	AT	intronic	GATAD2A	.	.	.	9.79E-09	8.80E-05
rs9614293	22	44347433	G	T	intergenic	PNPLA3;SAMM50	dist=3971;dist=3889	.	.	9.81E-09	2.90E-01
rs2074298	19	19377716	C	G	intronic	TM6SF2	.	.	.	9.95E-09	4.90E-04
rs14315	22	44372632	C	T	exonic	SAMM50	.	synonymous SNV	SAMM50:NM_015380:exon9:c.C780T:p.H260H	1.00E-08	1.80E-02
rs7249692	19	19670688	C	T	intergenic	CILP2;PBX4	dist=13220;dist=1834	.	.	1.01E-08	2.30E-02
rs11090620	22	44375742	C	T	intronic	SAMM50	.	.	.	1.08E-08	1.80E-02
rs5764043	22	44346965	G	A	intergenic	PNPLA3;SAMM50	dist=3503;dist=4357	.	.	1.08E-08	3.10E-01
rs5764045	22	44347250	C	T	intergenic	PNPLA3;SAMM50	dist=3788;dist=4072	.	.	1.08E-08	3.10E-01
rs626283	19	54677001	G	C	upstream;downstream	TMC4;MBOAT7	dist=147;dist=105	.	.	1.09E-08	7.90E-02
rs2235775	22	44375275	C	A	intronic	SAMM50	.	.	.	1.09E-08	1.90E-02
rs5764044	22	44347137	C	G	intergenic	PNPLA3;SAMM50	dist=3675;dist=4185	.	.	1.10E-08	3.10E-01
rs2965200	19	19476365	A	G	intergenic	MAU2;GATAD2A	dist=6802;dist=20287	.	.	1.11E-08	3.90E-04
19:19438110_GTATT_G	19	19438110	GTATT	G	.	.	.	.	.	1.13E-08	2.30E-03
rs8103250	19	19679461	G	A	intronic	PBX4	.	.	.	1.23E-08	2.00E-02
rs9304962	19	19679992	C	G	intronic	PBX4	.	.	.	1.23E-08	2.00E-02
rs7248200	19	19744452	C	T	intronic	GMIP	.	.	.	1.25E-08	4.00E-04
rs2269873	19	19392401	T	C	intronic	SUGP1	.	.	.	1.27E-08	2.40E-04
rs2315024	19	19423817	T	A	intronic	SUGP1	.	.	.	1.33E-08	2.90E-04
rs10407952	19	19394396	C	T	intronic	SUGP1	.	.	.	1.35E-08	1.90E-04
rs2315025	19	19426609	C	T	intronic	SUGP1	.	.	.	1.40E-08	1.90E-04
rs735273	19	19385411	T	C	intergenic	TM6SF2;SUGP1	dist=1301;dist=1431	.	.	1.43E-08	2.30E-04
rs8103496	19	19386015	A	G	downstream	SUGP1	dist=827	.	.	1.43E-08	2.30E-04
rs2017964	19	19387149	T	C	UTR3	SUGP1	NM_172231:c.*308A>G	.	.	1.43E-08	2.30E-04
rs8101938	19	19390497	G	A	intronic	SUGP1	.	.	.	1.43E-08	2.30E-04
rs2301784	19	19390749	G	A	intronic	SUGP1	.	.	.	1.43E-08	2.30E-04
rs6511026	19	19391402	C	T	intronic	SUGP1	.	.	.	1.43E-08	2.30E-04
rs2074303	19	19381755	C	T	intronic	TM6SF2	.	.	.	1.44E-08	2.30E-04
rs10419245	19	19383755	G	A	intronic	TM6SF2	.	.	.	1.44E-08	2.30E-04
rs6511028	19	19395967	T	C	intronic	SUGP1	.	.	.	1.50E-08	1.90E-04
rs7254748	19	19397479	A	C	intronic	SUGP1	.	.	.	1.51E-08	1.90E-04
rs4808937	19	19398005	A	G	intronic	SUGP1	.	.	.	1.51E-08	1.90E-04
rs10409234	19	19403410	A	G	intronic	SUGP1	.	.	.	1.52E-08	1.90E-04
rs2074299	19	19380646	T	C	intronic	TM6SF2	.	.	.	1.52E-08	2.30E-04
rs56077346	22	44375970	A	G	intronic	SAMM50	.	.	.	1.53E-08	1.70E-02
rs7254927	19	19404786	C	G	intronic	SUGP1	.	.	.	1.56E-08	1.90E-04
rs2023882	19	19405405	T	C	intronic	SUGP1	.	.	.	1.56E-08	1.90E-04
rs4808938	19	19401218	G	A	intronic	SUGP1	.	.	.	1.56E-08	1.90E-04
22:44345952_AAAAAAAT_A	22	44345952	AAAAAAAT	A	.	.	.	.	.	1.57E-08	3.50E-01
rs7252981	19	19692579	C	T	intronic	PBX4	.	.	.	1.59E-08	2.30E-02
rs8108705	19	19675367	T	G	intronic	PBX4	.	.	.	1.60E-08	2.30E-02
19:19394249_GTT_G	19	19394249	GTT	G	.	.	.	.	.	1.67E-08	1.80E-04
rs2294918	22	44342116	G	A	exonic	PNPLA3	.	synonymous SNV	PNPLA3:NM_025225:exon9:c.A1300A:p.K434K	1.68E-08	4.40E-04
rs35534408	19	19409021	C	CAG	intronic	SUGP1	.	.	.	1.71E-08	2.00E-04
rs2315022	19	19413381	A	G	intronic	SUGP1	.	.	.	1.72E-08	1.90E-04
rs2301786	19	19413947	A	G	intronic	SUGP1	.	.	.	1.72E-08	1.90E-04
rs2074296	19	19373689	A	G	upstream	HAPLN4	dist=76	.	.	1.74E-08	1.70E-04
rs9614294	22	44356566	G	A	intronic	SAMM50	.	.	.	1.76E-08	3.30E-01
rs2235772	22	44369329	C	T	intronic	SAMM50	.	.	.	1.76E-08	1.80E-02
rs1010207	19	19416045	T	C	intronic	SUGP1	.	.	.	1.78E-08	1.90E-04
rs2064361	22	44356349	C	G	intronic	SAMM50	.	.	.	1.79E-08	3.50E-01
rs10407283	19	19744358	T	C	intronic	GMIP	.	.	.	1.85E-08	4.20E-04
rs2285859	19	19422485	A	G	intronic	SUGP1	.	.	.	1.90E-08	1.90E-04
rs4808942	19	19420581	C	T	intronic	SUGP1	.	.	.	1.91E-08	1.90E-04
rs12977937	19	19429975	C	G	intronic	SUGP1	.	.	.	1.91E-08	1.90E-04
rs10426780	19	19375883	T	C	intronic	TM6SF2	.	.	.	1.93E-08	2.00E-04
rs8736	19	54677189	C	T	UTR3	MBOAT7	NM_001146083:c.*549G>A;NM_001146056:c.*549G>A;NM_024298:c.*549G>A	.	.	1.96E-08	1.20E-01
rs34564463	19	54676814	G	GC	UTR5	TMC4	NM_001145303:c.-2C>GC;NM_144686:c.-2C>GC	.	.	1.96E-08	1.00E-01
rs5764047	22	44358812	G	A	intronic	SAMM50	.	.	.	1.97E-08	3.70E-01
rs2076208	22	44331060	C	G	intronic	PNPLA3	.	.	.	2.15E-08	2.30E-01
19:19404387_CA_C	19	19404387	CA	C	.	.	.	.	.	2.15E-08	2.20E-04
rs2073082	22	44360007	G	A	intronic	SAMM50	.	.	.	2.29E-08	3.80E-01
rs7245672	19	19699963	C	T	intronic	PBX4	.	.	.	2.37E-08	2.10E-02
rs11668882	19	54675097	T	C	intronic	TMC4	.	.	.	2.37E-08	1.00E-01
rs2073083	22	44360010	C	A	intronic	SAMM50	.	.	.	2.69E-08	3.90E-01
rs1009136	19	19440428	G	A	intronic	MAU2	.	.	.	2.94E-08	1.80E-04
rs67720221	19	19440864	T	C	intronic	MAU2	.	.	.	3.10E-08	1.70E-04
rs3764567	19	19440066	T	C	intronic	MAU2	.	.	.	3.15E-08	2.10E-04
rs2074091	19	19450080	G	A	intronic	MAU2	.	.	.	3.37E-08	1.60E-04
rs12460764	19	19431963	T	G	intronic	MAU2	.	.	.	3.41E-08	1.90E-04
rs7246748	19	19433105	T	C	intronic	MAU2	.	.	.	3.41E-08	1.90E-04
rs7254230	19	19434350	T	C	intronic	MAU2	.	.	.	3.41E-08	1.90E-04
rs62135552	19	19436854	A	G	intronic	MAU2	.	.	.	3.41E-08	1.90E-04
rs7247309	19	19439631	T	A	intronic	MAU2	.	.	.	3.41E-08	1.90E-04
rs2905427	19	19478023	T	G	intergenic	MAU2;GATAD2A	dist=8460;dist=18629	.	.	3.52E-08	3.50E-04
rs13964	19	19468710	G	C	UTR3	MAU2	NM_015329:c.*2119G>C	.	.	3.73E-08	1.80E-04
rs15622	19	19468734	A	G	UTR3	MAU2	NM_015329:c.*2143A>G	.	.	3.73E-08	1.80E-04
rs10623726	19	19382307	C	CTCTT	intronic	TM6SF2	.	.	.	3.82E-08	2.50E-04
rs10640109	19	19443406	CAAG	C	.	.	.	.	.	3.83E-08	1.70E-04

Table continues on next page.

ID	Chr	POS	REF	ALT	Func.refGene	Gene.refGene	GeneDetail.refGene	ExonicFunc.refGene	AAChange.refGene	P	p_value
rs2965198	19	19473030	G	A	intergenic	MAU2;GATAD2A	dist=3467;dist=23622	.	.	4.09E-08	3.90E-04
rs2073088	22	44373579	G	A	intronic	SAMM50	.	.	.	4.42E-08	3.80E-01
rs2905424	19	19473445	C	T	intergenic	MAU2;GATAD2A	dist=3882;dist=23207	.	.	4.50E-08	3.20E-04
rs34538000	19	19481379	T	G	intergenic	MAU2;GATAD2A	dist=11816;dist=15273	.	.	4.53E-08	5.70E-04
rs2965175	19	19481606	A	G	intergenic	MAU2;GATAD2A	dist=12043;dist=15046	.	.	4.53E-08	5.80E-04
rs12985655	19	19442434	A	G	intronic	MAU2	.	.	.	4.61E-08	1.70E-04
rs8103197	19	19443466	T	C	intronic	MAU2	.	.	.	4.61E-08	1.70E-04
rs2074090	19	19449686	T	G	exonic	MAU2	.	synonymous SNV	MAU2:NM_015329:exon5:c.G489G:p.S163S	4.61E-08	1.70E-04
rs2965191	19	19453521	G	T	intronic	MAU2	.	.	.	4.61E-08	1.70E-04
rs2301669	19	19453560	A	C	intronic	MAU2	.	.	.	4.61E-08	1.70E-04
rs2965199	19	19475088	A	G	intergenic	MAU2;GATAD2A	dist=5525;dist=21564	.	.	4.63E-08	4.10E-04
rs2315281	19	19480099	A	G	intergenic	MAU2;GATAD2A	dist=10536;dist=16553	.	.	4.77E-08	5.40E-04
rs9614300	22	44362815	C	T	intronic	SAMM50	.	.	.	4.77E-08	3.60E-01
rs8101499	19	19476984	G	A	intergenic	MAU2;GATAD2A	dist=7421;dist=19668	.	.	4.85E-08	5.20E-04
rs12459854	19	19461437	C	T	intronic	MAU2	.	.	.	4.86E-08	1.70E-04

Table continues on next page.

Supplementary references

1. Bycroft C, Freeman C, Petkova D, et al. The UK Biobank resource with deep phenotyping and genomic data. *Nature*. 2018;562:203-209.
2. Chang CC, Chow CC, Tellier LC, Vattikuti S, Purcell SM, Lee JJ. Second-generation PLINK: rising to the challenge of larger and richer datasets. *Gigascience*. 2015;4:7.
3. Li MX, Yeung JM, Cherny SS, Sham PC. Evaluating the effective numbers of independent tests and significant p-value thresholds in commercial genotyping arrays and public imputation reference datasets. *Hum Genet*. 2012;131:747-756.
