# Supplementary material for: Genetic variants influencing liver fat in normal-weight individuals of European ancestry
Source: JHEP Rep. 2025 May 14;7(8):101453. doi: 10.1016/j.jhepr.2025.101453 (PMC12270618; doi:10.1016/j.jhepr.2025.101453)
Supplement: Multimedia component 2 [file mmc2.docx]

**JHEP Reports**

**CTAT methods**

Tables for a “Complete, Transparent, Accurate and Timely account” (CTAT) are now mandatory for all revised submissions. The aim is to enhance the reproducibility of methods.

- Only include the parts relevant to your study
- Refer to the CTAT in the main text as ‘Supplementary CTAT Table’
- Do not add subheadings
- Add as many rows as needed to include all information
- Only include one item per row

**If the CTAT form is not relevant to your study, please outline the reasons why:**

|  |
| --- |

- 1. **Antibodies**

| **Name** | **Citation** | **Supplier** | **Cat no.** | **Clone no.** |
| --- | --- | --- | --- | --- |
|  |  |  |  |  |

- 1. **Cell lines**

| **Name** | **Citation** | **Supplier** | **Cat no.** | **Passage no.** | **Authentication test method** |
| --- | --- | --- | --- | --- | --- |
|  |  |  |  |  |  |

- 1. **Organisms**

| **Name** | **Citation** | **Supplier** | **Strain** | **Sex** | **Age** | **Overall n number** |
| --- | --- | --- | --- | --- | --- | --- |
|  |  |  |  |  |  |  |

- 1. **Sequence based reagents**

| **Name** | **Sequence** | **Supplier** |
| --- | --- | --- |
|  |  |  |

- 1. **Biological samples**

| **Description** | **Source** | **Identifier** |
| --- | --- | --- |
|  |  |  |

- 1. **Deposited data**

| **Name of repository** | **Identifier** | **Link** |
| --- | --- | --- |
|  |  |  |

- 1. **Software**

| **Software name** | **Manufacturer** | **Version** |
| --- | --- | --- |
| PLINK | Open Source  Shaun Purcell & Christopher Chang | 2.0 |
| MAGMA | Open Source  de Leeuw C, Mooij J, Heskes T, Posthuma D | 1.10 |
| FUSION | Open Source  Gusev Lab (Harvard University & Broad Institute) | - |
| FINEMAP | Open Source  Benner C, Spencer C, Havulinna AS, Salomaa V, Ripatti S, Pirinen M | 1.4.2 |
| FOCUS | Open Source  Mancuso Lab (University of South California) | 0.9 |

- 1. **Other (*e.g*. drugs, proteins, vectors etc.)**

|  |  |  |
| --- | --- | --- |
|  |  |  |

- 1. **Please provide the details of the corresponding methods author for the manuscript:**

| Ignazio Piras, Translational Genomics Research Institute, Phoenix, AZ (ipiras@tgen.org) |
| --- |

**2.0 Please confirm for randomised controlled trials all versions of the clinical protocol are included in the submission. These will be published online as supplementary information.**

|  |
| --- |
